# CRISPRcasIdentifier: Machine learning for accurate identification and classification of CRISPR-Cas systems

**DOI:** 10.1093/gigascience/giaa062

**Published:** 2020-06-17

**Authors:** Victor A Padilha, Omer S Alkhnbashi, Shiraz A Shah, André C P L F de Carvalho, Rolf Backofen

**Affiliations:** 1 Institute of Mathematics and Computer Sciences, University of São Paulo, Av. Trabalhador São Carlense 400, São Carlos, SP, 13566-590, Brazil; 2 Bioinformatics Group, University of Freiburg, Georges-Köhler-Allee 106, 79110 Freiburg, Germany; 3 COPSAC, Copenhagen University Hospitals Herlev and Gentofte, Ledreborg Alle 34, DK-2820 Gentofte, Denmark; 4 Signalling Research Centres BIOSS and CIBSS, University of Freiburg, Schaenzlestr. 18, 79104 Freiburg, Germany

**Keywords:** CRISPR-Cas, machine learning, Cas genes, Cas proteins

## Abstract

**Background:**

CRISPR-Cas genes are extraordinarily diverse and evolve rapidly when compared to other prokaryotic genes. With the rapid increase in newly sequenced archaeal and bacterial genomes, manual identification of CRISPR-Cas systems is no longer viable. Thus, an automated approach is required for advancing our understanding of the evolution and diversity of these systems and for finding new candidates for genome engineering in eukaryotic models.

**Results:**

We introduce CRISPRcasIdentifier, a new machine learning–based tool that combines regression and classification models for the prediction of potentially missing proteins in instances of CRISPR-Cas systems and the prediction of their respective subtypes. In contrast to other available tools, CRISPRcasIdentifier can both detect *cas* genes and extract potential association rules that reveal functional modules for CRISPR-Cas systems. In our experimental benchmark on the most recently published and comprehensive CRISPR-Cas system dataset, CRISPRcasIdentifier was compared with recent and state-of-the-art tools. According to the experimental results, CRISPRcasIdentifier presented the best Cas protein identification and subtype classification performance.

**Conclusions:**

Overall, our tool greatly extends the classification of CRISPR cassettes and, for the first time, predicts missing Cas proteins and association rules between Cas proteins. Additionally, we investigated the properties of CRISPR subtypes. The proposed tool relies not only on the knowledge of manual CRISPR annotation but also on models trained using machine learning.

## Background

CRISPR-Cas systems provide archaea and bacteria with a nucleic acid–based adaptive immune system against invading viruses and plasmids. Mechanistically, the immune response can be divided into 3 stages, namely, adaptation, processing, and interference, each carried out by different sets of protein complexes [1]. The universally conserved proteins Cas1, Cas2, and optionally Cas4 are responsible for the adaptation stage, when a fragment of invader DNA is excised and stored in the host chromosome as a spacer in the non-coding CRISPR (clustered regularly interspaced short palindromic repeats) region. The processing and interference stages are much more mechanistically diverse, using different sets of proteins, depending on the type of CRISPR-Cas system. CRISPR-Cas systems are found in many bacteria and most archaea and have diversified as much as their host organisms [2].

While the mechanistic principles are similar, with spacers comprising templates for synthesis of CRISPR interference RNAs (crRNAs) against the invader, the various types and classes of CRISPR-Cas systems show some important differences. Class 2 systems use a single multi-domain protein for locating and cleaving the re-invading nucleic acid, whereas Class 1 systems use a large multi-subunit complex for the same purpose. Class 2 systems can be further subdivided into types II, V, and VI, which seem to have evolved independently from each other. Thus, the respective Cas9, 12, and 13 enzymes that carry out invader cleavage rely on diverse mechanisms involving differing nuclease domains [2–4].

Class 1 systems, on the other hand, with types I, III, and IV, use structurally related proteins to carry out similar functions, although the protein subunits have diverged considerably. Common to all Class 1 systems is that Cas7 forms a helical backbone that spans the length of the tightly bound crRNA. This backbone is terminated in one end by Cas5, which itself is bound to Cas8 or Cas10 for type I and IV or type III systems, respectively. Type I systems use Cas8 for the recognition of the protospacer adjacent motif [5], which, along with invader crRNA hybridization, comprises a signal for recruitment of the Cas3 helicase-nuclease protein that subsequently digests the invader chromosome [6]. Type III systems, however, use the Cas7 backbone for cleaving invader messenger RNA while the Cas10 HD nuclease cleaves transcribed DNA [7,8]. Cas10 also synthesizes a signaling molecule that recruits additional accessory Cas proteins for other functions, such as cell suicide or activation of other defense systems [8, 9].

The different types of CRISPR-Cas systems are themselves so diverse that each type can be further subdivided into several subtypes. Type III, for example, is divided into 4 subtypes III-A, B, C, and D. While CRISPR-Cas systems of the same subtype encode similar proteins that occupy the same roles, the proteins have often diverged beyond the point of recognition by conventional sequence alignment methods such as BLAST, even within a subtype. This level of sequence diversity makes proper identification of the found CRISPR-Cas systems very challenging, and the field has thus far relied upon the gold standard of periodic manual annotations by experts, published once every few years [2,10,11]. The annotation involves profile hidden Markov models (HMM) searches for finding core genes, followed by the inspection of their neighbourhoods, gauging operonic structures, and manual BLAST and PSI-BLAST searches [12]. With the increasing number of genome sequences from uncultured microbes and metagenomic data, however, manual annotations cannot keep up and an automated approach is needed that would yield accuracy comparable to that of manual annotation. Furthermore, research groups working on organisms not yet covered by published annotations have thus far made their own manual annotations, leading to inconsistencies in nomenclature and inaccuracies in some cases.

There have been numerous attempts at devising computational pipelines for the identification of different elements of the CRISPR system, such as CRISPR arrays [13–15] and CRISPR leaders [16]. On the other hand, command line tools and webservers, usually based on HMM and HMMER [17] or PSI-BLAST [12], have been proposed for CRISPR subtype prediction. Examples of such tools are CRISPRdisco [18], CRISPRcasFinder [19], Macsyfinder [20], CRISPRone [21], and HmmCas [22]. We found, however, that the existing tools usually lack the ability to generalize unseen examples. Additionally, these tools can neither adapt to an extending repertoire of *cas* genes, predict possibly missing proteins, nor learn association rules among proteins.

In this work, we present a machine learning (ML) approach intending to capture much of the relevant essence of manual annotation. It is based on evidence for the different Cas proteins to be contained in a series of consecutive genes that are part of a cas cassette and thus represents genomic CRISPR-Cas systems as cassettes of adjacently encoded proteins. These pieces of evidence are calculated by newly designed sets of HMM models for each Cas protein, covering the diversity of Cas protein families. The proposed approach solves the problem of classification of new systems into types and subtypes. Because our features for the ML approach correspond to evidence for Cas proteins, we can determine Cas proteins whose evidence is critical for predicting a subtype, which corresponds to the concept of signature genes. We show that our approach correctly identifies known signature genes for types and subtypes. In addition, our approach is able to provide more information about the composition of cassettes. One application is to predict evidence for Cas proteins that have been missed in the Cas protein screening. This provides researchers with hints to search for remote homologs of the missing Cas proteins, or for new proteins that might replace the associated function. Furthermore, we are able to learn association rules, which are subsets of proteins being important to each other, indicating functional modules. As a proof of concept, when we search for Cas proteins associated with an interference protein, our approach finds other interference proteins to be most important. The more interesting cases undoubtedly involve non-interference proteins, where our tool could correctly predict a strong association of the ancillary protein Csn2 with Cas1, consistent with its hypothesized role in adaptation. For the protein CasR we found that it is associated with different functional modules in subtypes I-A and I-E, indicating a possible functional diversity. Thus, the set of protein associations derived in this manner provides a proper resource for researchers who want to investigate the function of different Cas proteins.

## Methods

### Data collection and preprocessing

All Cas proteins used in this study were selected from the current classified archaeal and bacterial CRISPR-Cas systems [2–4]. We performed an all-against-all sequence similarity comparison on these data using Fasta [23]. Subsequently, we clustered the proteins using the Markov Cluster Algorithm (MCL) [24] based on custom similarity criteria [9,16]. These criteria consider the size of the proteins, the length alignment, and the relative locations of similar regions between the 2 compared proteins. After clustering the protein sequences from a specific Cas protein family, we generated a multiple sequence alignment using MUSCLE [25]. Next, these alignments were converted to HMM profile models by using hmmbuild [17]. Except for MCL, all other tools were run with default parameters.

Throughout the text, each cassette in our training and test datasets is represented by a tuple consisting of its genomic sequence containing all genes of the cassette, and the list of all annotated Cas proteins. We extracted the genomic sequences as follows: we took Supplementary Table S7 from Makarova et al. [2], which contains all gene loci (i.e., genomic positions) in column “(sub)Type / Coordinates,” and downloaded the sequences from NCBI. For the second part, namely, the list of all Cas proteins, we extracted the genomic sequence for each annotated Cas protein individually, again from the “coordinates” column, and added 50 bases of context. The associated amino acid sequences were generated by running the Prodigal tool v2.6.3 [26] on the respective gene sequences, and stored together with the Cas annotation from the column “*cas* gene” in Table S7 from Makarova et al. [2].

To generate the feature vectors, we ran all HMM profile models using hmmsearch against the sequences of all cassettes. We selected the cassettes that had a hit for all proteins annotated for that subtype and used this as training and test set for the classification pipeline. Cassettes that had a missing protein were used instead as an independent test case for our regression models and the full pipeline.

### Classification of Cas cassettes

For this task, we apply ML algorithms onto a finite sample of CRISPR data to obtain predictive models that are able to classify Cas cassettes into their respective subtypes using a data matrix representation (see Results and Discussion). Thus, based on the finite sample of data, we investigate the application of classification algorithms that estimate a function that is able to generalize the association between a cassette and its subtype. As a consequence, we intend to use this function to classify new cassettes that were not seen during the training phase into their respective subtypes with a high level of accuracy.

### Prediction of missing Cas proteins

We also investigate the problem of predicting (possibly) missing Cas proteins by estimating their normalized bit scores. For this problem, we modelled it as follows. Given *m* Cas proteins, we filter, for each subtype, its set of *l* < *m* proteins (i.e., all Cas proteins whose bit score is >0 for ≥1 cassette of the subtype). Next, we train *l* regressors, where the *j*th regressor, *j* ∈ {1, ⋅⋅⋅, *l*}, predicts the bit score of the *j*th Cas protein using the remaining *l* − 1 proteins as input.

### Experimental evaluation of ML algorithms

Three ML algorithms were applied to the preprocessed dataset to train classification and regression models:

- Classification and Regression Trees (CART) [27], which trains a predictive model represented by a decision tree. This algorithm can train decision trees for classification (classification trees) and regression (regression trees) tasks. A decision tree is composed by a set of interpretable rules extracted from the training dataset. These rules explain the decisions made by the model to predict the class or regression value for new, previously unseen, examples.
- Support Vector Machines (SVM) [28], which trains a binary classifier represented by a hyperplane that separates examples from 2 classes with the maximum possible separation margin. By using kernel functions, an SVM can be applied to non-linearly separable problems. For multiclass classification tasks, a multiclass dataset is usually first decomposed into several classification binary datasets. SVMs can then be applied to each binary dataset, and their predictions are combined for a multiclasss classification.
- Extremely Randomized Trees (ERT) [29], which uses an ensemble of decision trees, where each tree is trained using a random subset of the original features. Instead of selecting the best discriminating threshold for each feature considered for a split, as would be the case for classical decision trees, ERT chooses a random threshold value. The final predictions are the average of the predictions of all the decision trees in the ensemble. We can extract the importance of each feature in the classification or regression task from the decision trees in the ensemble. The importance is represented by the decrease in impurity caused by a node that splits the feature, weighted by the number of examples contained in such a node [30], and averaged over all trees of the ensemble.

The model selection and evaluation of predictive models is a widely studied problem in the ML literature. Several works (e.g., [31–33]) investigate the advantages and drawbacks of different methodologies. On the basis of these previous studies, we use the nested cross-validation procedure. Given a set of data, the classical cross-validation approach splits the data into *K* mutually exclusive and similar sized subsets called folds. Next, at each iteration, *K* − 1 folds are used for training an ML model and the remaining fold for testing it [34,35]. The nested cross-validation approach separates the model selection and evaluation steps by using 2 different cross-validation loops: an outer loop, which splits the data into *K*_1_ folds and is used for model evaluation; and an inner loop, which splits the training data into *K*_2_ folds and is used for model selection. In this article, we set *K*_1_ = *K*_2_ = 10, and repeat the evaluation procedure 50 times, owing to the variance of the results when considering different splits [33]. It is important to mention that, during our experiments, to guarantee that examples from all classes are present in each outer fold, we used only classes containing ≥10 examples.

For each cross-validation iteration, we aggregate the predictions from all folds and calculate a single predictive performance evaluation, in order to avoid any averaging problems that might arise, especially when the dataset is imbalanced [36]. For the classification experiments, we used the following evaluation measures: adjusted balanced accuracy score [37,38], an adaptation of the original accuracy measure that gives higher weights to examples from smaller classes; and the F-score with macro-averaging [39], which is the average F-score among all classes. Both measures treat different subtypes equally. Thus, they do not favour those with the largest numbers of cassettes. For the regression experiments, we used the mean absolute error [40], which is the average absolute difference between the expected and the predicted target values.

Regarding the model selection step of each ML algorithm used, we performed a grid search over 20 different hyperparameter combinations, based on the guidelines from the scikit-learn package [41]. We describe these hyperparameter grids next. For the CART algorithm, we varied the hyperparameters that determine the maximum depth of the decision tree and the minimum number of examples necessary for a node to become a leaf. For the former, we considered the values in {5, 10, 15, max }, where max  allows the tree to grow as deep as possible. For the latter, we varied the values in {5, 6, 7, 8, 9}. For the SVM algorithm, we used a Gaussian kernel, owing to its ability to model nonlinear decision boundaries and its reduced number of hyperparameters when compared with another commonly used nonlinear kernel, the polynomial kernel [42]. For the cost hyperparameter *C*, we considered the values in {1; 10; 100; 1,000}. Regarding the kernel coefficient γ, we assessed the values in {0.01, 0.1, 1, 10, 100}. Finally, for the ERT algorithm, we varied the ensemble size using the values in {25, 50, 75, 100}, and the quantity of features to be considered when performing a split from the set of values in $\lbrace 25\%, 50\%, 75\%, 100\%, \sqrt{m} \rbrace$, where *m* is the number of known Cas protein families.

## Results and Discussion

### A combined approach to determine Cas proteins and cassette subtypes

The classification of a subtype is based on the membership for specific Cas proteins. Thus, any ML-based classification of a cassette requires the detection of the contained Cas proteins as a first step. While this first step is commonly performed using HMM, a difficulty arises from the fact that a single Cas protein family has to be split into different subfamilies owing to the high evolutionary diversity of their members. Owing to missing values in the dataset for a family, even the problem of splitting into different subfamilies is not an easy one. Even further, we have observed that the splitting of Cas protein families influences the quality of ML-based subtype classification. This would be quite obvious if subfamilies of individual Cas protein correlated well with subtypes. The real situation, however, is more complex, partially owing to the fact that cassettes are composed in a modular way, often involving horizontal gene transfer [2,9].

In brief and as described in more detail below, our classification approach takes the bit scores for the contained Cas proteins as evidence of their membership in the cassette. We use this information to apply a set of ML algorithms to classify the subtypes of cassettes. By generating different divisions of subfamilies for each Cas protein, we obtain different pieces of evidence for the contained Cas proteins. Thus, we can investigate which division is best related to subtype evolution. With this holistic view of Cas protein and subtype annotation, we can further examine relations between subtypes and Cas protein membership and as a result reassess key components of subtypes such as signature genes.

### Detection of Cas proteins by families of HMMer models

Our definition of Cas protein subfamilies is based on clustering the known sequences of a specific Cas protein family. We use ∼68,594 Cas proteins as a database, and applied different cluster criteria. Each cluster characterizes a subfamily, which is afterwards represented by an HMM model. All models for a Cas protein are grouped, and the best-matching HMM for each Cas protein is used to score a new sequence. To cluster the sequences, we performed an all-against-all sequence similarity comparison. Subsequently, we applied the MCL [24] to cluster the known sequences for a specific Cas protein family according to their sequence similarities. However, protein sequences can be clustered in different ways, depending on the cut-off for sequence similarity and the requested coverage of the alignment between 2 sequences. In addition, different hyperparameters for the MCL clustering algorithm result in different data partitions. Each partition defines different subfamilies, for which we train HMM models.

The different clustering approaches thus result in HMM models for different subfamilies, with varying specificity and sensitivity to detect members of a Cas protein family. We created 5 different collections of HMM models labelled HMM_1_ … HMM_5_ using different hyperparameter values for the clustering algorithm and distinct threshold values for the all-against-all sequence similarity detection (see Methods for details; the number of models for each Cas protein family is listed in Supplementary Table S1). For a given Cas protein sequence, we applied all HMM models that are contained in a specific collection for that protein family and took the maximum bit score, and zero otherwise. Non-zero values indicate that the investigated protein sequence belongs to the Cas protein family defined by the HMMer model set.

We used different measurements to assess the quality of a specific division represented by a set HMM_*i*_. One quality criterion for a set HMM_*i*_ is clearly the capability for detecting known members of Cas proteins. Table 1 shows the sensitivity for the 5 sets HMM_1_ … HMM_5_ by reporting the number of cassettes found in each subtype. It is easy to see that the more fine-grained sets, HMM_1_, HMM_2_, and HMM_3_, clearly detect more Cas proteins than the less fine-grained sets HMM_4_ and HMM_5_.

**Table 1.** Properties and Quality Measurements for the collections HMM_1_ … HMM_5_

Parameter	HMM_1_	HMM_2_	HMM_3_	HMM_4_	HMM_5_
No. models	379	385	416	209	201
No. sequences	14,674	14,674	23,622	16,018	16,018
Sensitivity per subtype					
I-A	116	116	117	0	0
I-B	715	715	713	421	421
I-C	629	629	629	612	612
I-D	138	138	137	100	100
I-E	1,114	1,114	1,116	1,069	1,069
I-F	354	354	353	339	339
I-U	136	136	82	8	8
II-A	320	320	331	249	249
II-B	28	28	35	35	35
II-C	327	327	333	328	328
III-A	376	376	364	326	326
III-B	292	292	290	178	178
III-C	93	93	93	83	83
III-D	184	184	186	49	49
IV-A	36	36	36	43	43
V-A	18	18	32	27	27
VI-A	6	6	4	6	6
VI-B	40	40	40	40	40
Total sensitivy	4,922	4,922	4,891	3,915	3,915
Accuracy (median)					
ERT	0.9900	0.9898	0.9909	0.9907	0.9907
CART	0.9629	0.9624	0.9636	0.9579	0.9583
SVM	0.9856	0.9856	0.9830	0.9868	0.9868

Sensitivity per subtype indicates sensitivity of set HMM_*i*_ in detecting Cas proteins, measured by the number of cassettes found per subtype. Sets HMM_1_, HMM_2_, and HMM_3_ are more fine grained than sets HMM_4_ and HMM_5_, which detect fewer Cas proteins overall. Accuracy indicates median accuracy for the classification of subtypes when using set HMM_*i*_ with different ML-approaches to determine the evidence for a Cas protein in a cassette. The quality difference is much lower in the overall task of subtype classification compared to the task of detecting individual Cas proteins.

In our holistic view of Cas protein detection and subtype classification, however, we also want to understand how the division into subfamilies relates to the cassette subtype and thus influences the subtype classification. For that reason, we show in Table 1 also as another quality criterion the median accuracy for correctly predicting the subtype of a cassette when using the HMM_*i*_ in an ML-based subtype classification approach as described in the next section. The surprising result is that the sensitivity of a specific set HMM_*i*_ in detecting Cas proteins does not correlate with the accuracy that is achieved in a subtype classification using this set HMM_*i*_.

### A pipeline for CRISPR cassette classification based on Cas protein evidence

**Figure 1:**
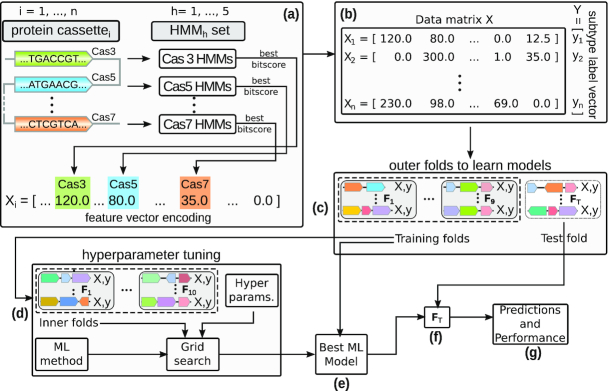
Experimental methodology adopted for this study. (a) Every cassette from our positive set is encoded into a feature vector, which has an entry for each Cas protein family. Given a specific cassette with known Cas proteins, we apply to each Cas protein sequence all HMMs from the set of HMMs that were generated for that specific Cas protein. The best bit score is included into the feature vector X_*i*_ encoding the *i*th cassette. (b) This feature vector is stored in the data matrix X, together with the known subtype. (c) Because the trained model highly depends on the collection of used cassettes, we use the 10-fold cross-validation strategy. Thus, we split the training set into 10 subsets called folds. We perform 10 runs, where, in each run, 1 of the folds is used for testing and the remaining 9 for selecting and training the best ML model. (d) For selecting the best ML model, a similar cross-validation strategy is applied to tune 20 hyperparameter combinations that affect the model predictive performance. Then, in (e), the selected model is trained using the whole training set. Finally, in (f) and (g) we apply the trained model to the respective test set of the outer fold and evaluate its performance.

Our classification pipeline for CRISPR cassettes is described in Fig. 1 and has 5 steps. For each set HMM_1_ … HMM_5_, we build a data matrix for classification and regression analysis of cassettes as follows. Usually, a CRISPR cassette $\mathcal {C}$ is a collection of Cas proteins and is thus defined as a subset of all known Cas proteins $\mathcal {P}$ (i.e., $\mathcal {C} \subset \mathcal {P}$). However, when predicting Cas proteins with HMMer models, this would imply a discretization of the bit score that would omit the information about the "evidence" we have for the prediction. For this reason, we define for each cassette $\mathcal {C}_i$ a real vector X_*i*_ of length *m*, where *m* is the number of known Cas protein families, containing an entry for each possible Cas protein. Each element X_*ij*_ is defined as the best bit score obtained by $\mathcal {P}_j$ among all HMM models of its family if it is detectable by the models, and zero otherwise (Fig. 1a). By concatenating the vectors obtained for all the *n* available cassettes, we obtain a data matrix X $\in \mathbb {R}_{+}^{n \times m}$ (Fig. 1b). In addition, each cassette is associated with a label that indicates its subtype, according to the classification provided by [2,4,9].

This data matrix, along with the feature vectors and the subtype labels for all known cassettes, is our training data for the subtype classification task. For the evaluation of our classification models, we apply a 10-fold cross-validation procedure on this data matrix. For this, we randomly split the data matrix X into 10 folds (Fig. 1c), each containing a subset of cassettes encoded by the associated feature vector. Each vector is annotated (labelled) by its true subtype. For model selection, we perform hyperparameter tuning by employing a grid search over 20 hyperparameter combinations and applying an inner cross-validation loop (Fig. 1d; see Methods for details). After selecting and training the best model (Fig. 1e), we have a classifier that, along with a feature vector with HMM bit scores for all known Cas protein families, predicts the subtype of new cassettes (Fig. 1f and g).

### The classification pipeline successfully predicts the subtype of cassettes

**Figure 2:**
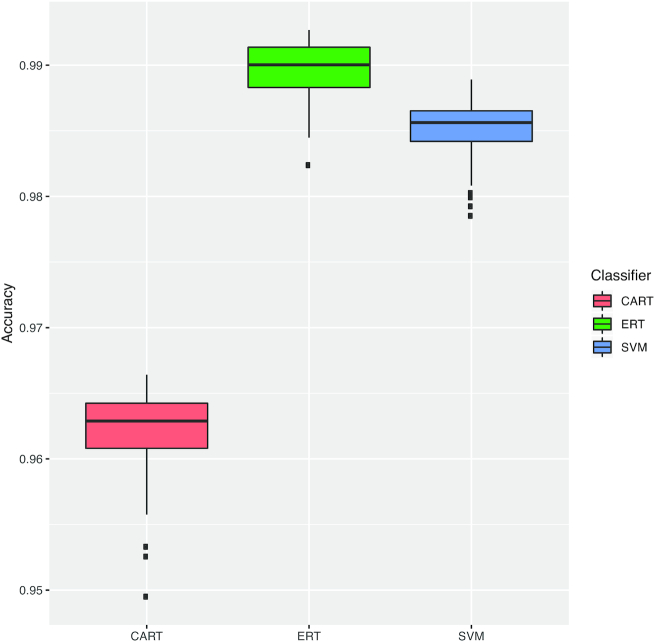
Adjusted balanced accuracy obtained for the 50 repetitions of nested 10-fold cross-validation applying ML algorithms to the dataset generated by the HMM_1_ set. The *x*-axis corresponds to the classifiers trained by different ML algorithms, represented as boxes with different colors. The *y*-axis shows the range of adjusted balanced accuracy values. Outliers are presented as square dots.

To evaluate the pipeline, we first assessed whether it can successfully perform the classification task, i.e., correctly predict the subtype of a cassette. As shown in Fig. 2 for HMM_1_, the predictive performances, measured by the adjusted balanced accuracy, for CART, ERT, and SVM algorithms are >95% in general. These high values suggest that, although imbalanced, the cassette subtypes are well defined in the feature space. It is important to mention that not all cassettes are complete in the investigated datasets. Some cassettes are composed only by subsets of the Cas proteins that integrate its subtype definition. In Supplementary Table S2, we summarize the percentage of cassettes that are complete for each subtype, ignoring Cas proteins that are contained in <5% of the cassettes of each subtype. We observed in the experimental results that, even though some incomplete cassettes are present, the 3 classifiers were still able to capture the relations among the remaining proteins. The results for the other 4 sets of HMM models, and for the F-score with the macro averaging measure, were similar and allowed us to draw similar conclusions (see Supplementary Fig. S1).

To investigate the prediction quality for specific subtypes, we performed an experiment using the "one-vs-the-rest" strategy [34]. Given *k* different classes, the one-vs-the-rest strategy trains *k* classifiers, 1 for each subtype, which learns how to discriminate this subtype (positive class) from the remaining classes (negative class). In Table 2 we report the average F-scores, after 50 cross-validation repetitions, obtained by the classifiers using the one-vs-the-rest strategy. It is clearly visible that the *k* classifiers were able to discriminate each class with a high predictive performance, in agreement with our previous results. In the case of SVM, one can use the margin separating positive and negative data as an additional quality criterion [43]. Again one can see here a clear separation of SVM scores for the positive and negative classes (see Supplementary Fig. S2).

**Table 2.** Mean F-scores for 50 nested cross-validation repetitions using the one-vs-the-rest strategy and Cas protein set HMM_1_. The best results for each subtype are in bold.

Subtype	CART	SVM	ERT
I-A	0.95	0.96	$\mathbf {0.98}$
I-B	0.95	0.98	$\mathbf {0.99}$
I-C	0.98	0.99	$\mathbf {1.00}$
I-D	0.98	0.97	$\mathbf {0.99}$
I-E	0.99	0.99	$\mathbf {1.00}$
I-F	0.95	0.99	$\mathbf {1.00}$
I-U	0.99	0.97	$\mathbf {1.00}$
II-A	$\mathbf {1.00}$	$\mathbf {1.00}$	$\mathbf {1.00}$
II-B	$\mathbf {1.00}$	$\mathbf {1.00}$	$\mathbf {1.00}$
II-C	$\mathbf {1.00}$	$\mathbf {1.00}$	$\mathbf {1.00}$
III-A	0.98	0.98	$\mathbf {0.99}$
III-B	0.97	0.98	$\mathbf {0.99}$
III-C	0.93	$\mathbf {0.98}$	$\mathbf {0.98}$
III-D	0.96	0.97	$\mathbf {0.99}$
IV-A	$\mathbf {1.00}$	$\mathbf {1.00}$	$\mathbf {1.00}$
V-A	$\mathbf {1.00}$	$\mathbf {1.00}$	$\mathbf {1.00}$
VI-B	0.86	0.95	$\mathbf {0.96}$

### The classification pipeline detects signature proteins

Makarova et al. [2] define the presence of unique signature Cas proteins that characterize most of the investigated CRISPR subtypes. According to the authors, signatures usually consist of either 1 or multiple Cas proteins that co-occur in the same cassette. On the basis of the aforementioned results, we hypothesize that the classifiers were able to learn these signature proteins. Because each one-vs-the-rest classifier introduced in the previous section learned how to discriminate a different subtype, we assessed whether it is possible to derive insights about signature proteins for each class by analyzing each classifier separately.

**Figure 3:**
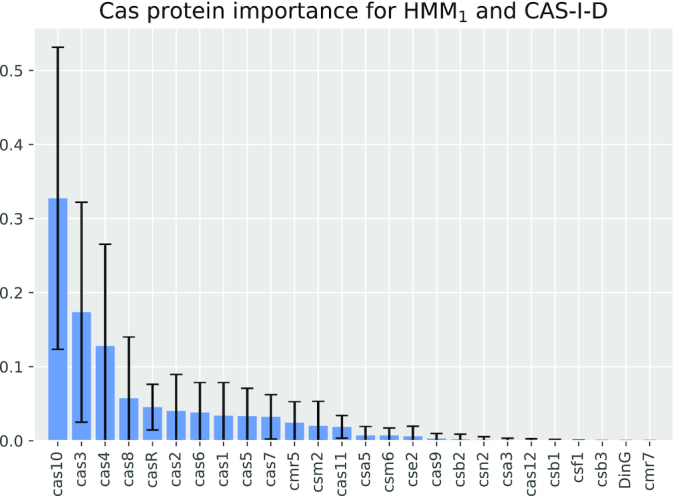
One-vs-the-rest mean Cas protein importance of ERT for I-D subtype. The *x*-axis presents different Cas proteins. The *y*-axis shows the importance of each Cas protein regarding the decision trees of the ensemble split. The error bars refer to the standard deviation over all trees in the ensemble. Note that the feature importance is not only related to the classification into the I-D subtype but may also be related to its contribution to classify a cassette into any other subtype. Thus, some of the proteins in the figure may not be related to I-D but to any other subtype.

We thus propose a new approach to detect signature proteins for a subtype by determining the importance of a specific feature (i.e., the evidence for a Cas protein in a cassette) to correctly predict the subtype in the respective one-vs-the-rest classifier. The rationale is that Cas proteins that are highly important for discriminating a specific subtype against all others are likely signature proteins for this subtype. Fig. 3 shows the importance of each Cas protein (see Methods for definition of feature importance) in predicting the I-D subtype. As can be seen, the importance is specifically high for Cas10(d) (respectively Cas3), which is the signature protein for Subtype I-D (respectively Type I) according to Makarova et al. [2]. Overall, we observed that Cas10 and Cas3 account, on average, for >50% of the feature importance for classifying the I-D subtype.

**Figure 4:**
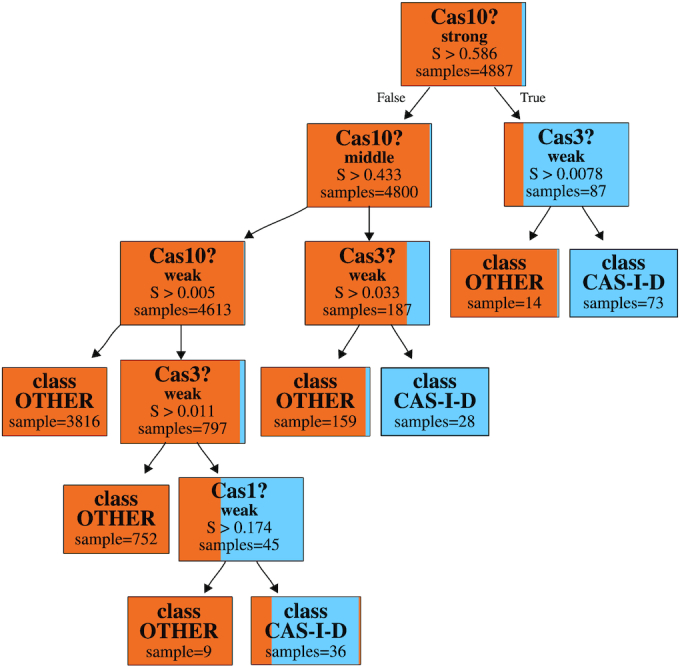
Reduced one-vs-the-rest CART for the I-D class (see Supplementary Fig. S3 for full tree). Cassettes that are labelled as Subtype I-D are highlighted in blue, the others in brown. Each node shows the fractions of class I-D and other cassettes, indicating the purity of the node. The number of cassettes is shown under the “samples” entry. In each node, we query for evidence of a specific Cas protein, indicated by the score calculated by the HMM family models. As one can see, strong evidence for Cas10 immediately points to Subtype I-D (top node and right branch). Otherwise, if we have middle evidence for Cas10, we need at least weak evidence for Cas3 to determine Subtype I-D. Finally, if we have only weak evidence for Cas10, we need at least weak evidence for Cas3 and also for Cas1 to determine Subtype I-D (left branch). However, the classification is not pure anymore (bottom nodes).

To investigate the relation between the 2 signature genes for proteins Cas10 and Cas3 in more detail, we selected the decision tree obtained by CART for the I-D subtype (Fig. 4). In this tree, terminal nodes with the blue colour indicate I-D classification (positive class), while those with brown colour indicate any other subtype classification (negative class). As shown in Fig. 4, Cas10 is the most important protein for identifying I-D, which is in agreement with Makarova et al. [2], where Subtype I-D is characterized by the presence of a variant of the Cas10 protein (instead of a protein from the Cas8 family, which is common for the other I subtypes) and 2 variants of the Cas3 protein. Interestingly, we need middle to strong evidence for Cas10 and only weak evidence for Cas3. In the case of weak evidence for Cas10, we also need weak evidence for both Cas3 and Cas1 in order to correct the missing 36 examples, albeit in this case the classification would not be pure anymore. Overall, it can be observed that CART was able to correctly model this signature because most of the nonterminal nodes refer to these proteins, indicating that they are the most important features in this subtype.

Because the current classification [2] is based only on the interference module, the adaptation-related Cas proteins (Cas1, Cas2, and Cas4) should not have a high importance for our classification pipeline. Thus, in another experiment, we removed these proteins and the processing proteins (Cas6) and tested the predictive performance of our classification pipeline when removing this information. The obtained results were similar to those previously discussed in this section and support our discussion and main conclusions (see Supplementary Fig. S4), strengthening the hypothesis that our ML-based approach captured biologically relevant information.

All the aforementioned examples illustrate how our ML models are able to learn the protein signatures without any extra information other than the normalized bit scores and cassette subtype labels. These results validate our hypothesis and provide models that are able to automatically categorize new cassettes with a high predictive accuracy.

### Regression instead of classification learns association rules

In our next set of experiments, we were interested in answering the question of whether some Cas proteins tend to co-occur frequently with other proteins. To answer this question, we hypothesized that they form a functional module. However, because we have varying information about the evidence for a specific Cas protein and there is also some redundancy and flexibility in forming this module, we followed an approach different from that described in the previous section. We believe that if a specific Cas protein is frequently associated with other Cas proteins, it is possible to predict the evidence for this protein by relying only on the known evidence for the other members of the functional module. We can confirm this belief by removing a specific Cas protein from the feature vector and predicting the “expected” normalized bit score for this protein from the remaining feature vector. This amounts to learning a regression model from known examples.

Association rules can now be inspected by determining again the important features (i.e., Cas proteins) to predict the correct evidence for a specific Cas protein. In Table 3, we list the 3 most important proteins for some target Cas proteins in some subtypes. In this case, for predicting evidence for Cas10d in Subtype I-D, we need the information about Cas3, Cas5, and Cas7. In agreement with the fact that subtypes are mainly associated with the interference complex [2], we find that for the interference proteins Cas10d, Cas3, and Cse2, the associated proteins are also interference proteins. For the non-interference proteins Csn2 and Cas4 in II-A and II-B, not only is Cas9 an interference and signature protein for Type II, but it is associated with them as well as the adaptation proteins Cas1 and Cas2. Interestingly, although Cas9 information is important for Cas4, Cas1 is actually more significant for Csn2. This is in agreement with the hypothesized role of Csn2 in the adaptation process [44–47].

**Table 3.** Top 3 most important proteins according to ERT when trying to predict a target protein across different subtypes

Subtype	Target protein	Most important proteins
I-D	Cas10d	(Cas3, 0.28), (Cas5, 0.26), (Cas7, 0.17)
I-D	Cas3	(Cas11, 0.48), (Cas10, 0.20), (Cas5, 0.11)
I-E	Cse2	(Cas7, 0.25), (Cas5, 0.23), (Cas8, 0.19)
II-A	Csn2	(Cas1, 0.62), (Cas9, 0.23), (Cas2, 0.15)
II-B	Cas4	(Cas9, 0.83), (Cas1, 0.09), (Cas2, 0.08)
I-A	CasR	(Csa5, 0.28), (Cas5, 0.16), (Cas6, 0.12)
I-E	CasR	(Cas1, 0.33), (Cas7, 0.25), (Cas8, 0.21)

For the interference proteins Cas10d, Cas3, and Cse2, the other most important Cas proteins are also interference proteins. For non-interference proteins, other Cas proteins linked to adaptation, e.g., Cas1 and Cas2, are also important. The helper protein CasR seems to have different modules associated in I-A and I-E.

An interesting case to consider is CasR (also known as CasRA or Csa3), a transcriptional regulator of CRISPR interference and/or adaptation [48,49]. This protein seems to play different roles in subtypes I-A and I-E and also appears to be associated with the different proteins in I-A and I-E (see Table 3, last 2 rows). In I-A, the most important proteins are Csa5, Cas5, and Cas6, whereas in I-E they are Cas1, Cas7, and Cas8.

### The ML approach can handle missing Cas proteins

During our experiments, we left out cassettes that had 1 or more Cas proteins missing, i.e., without hits in their corresponding HMM models during the preprocessing step (Fig. 1a). Because these cases often occur in real application scenarios, it is important to assess how our ML-based pipeline can handle them. We observed that most of these cassettes contained only 1 protein that did not present any hit for the HMM models of its family. For such, we worked with the cassettes having all proteins annotated as ground truth, and removed 1 bit score for a specific protein. We then learned a model able to predict this bit score using the evidence information from the remaining proteins.

Specifically, we investigated the performance of predicting the missing evidence using the previously described regression approach, trained on all subtypes. The basic idea is that finding high-quality predicted evidence for a missing protein is a hint for researchers to perform an in-depth attempt to either annotate the missing protein or to search for new proteins that might replace the function of the missing protein.

**Figure 5:**
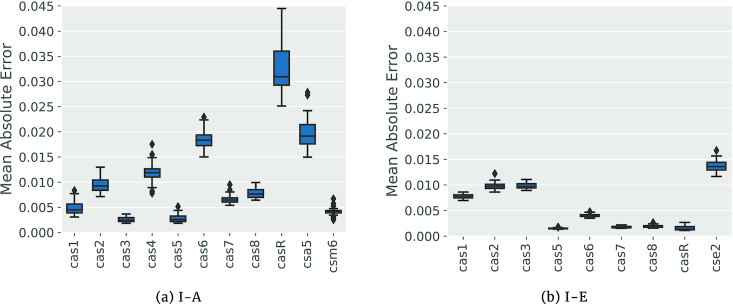
Mean absolute error rates for Cas proteins contained in I-A (a) and I-E (b) subtypes over 50 nested cross-validation repetitions. The *x*-axis lists the different Cas proteins that were used as target variables. The *y*-axis presents the mean absolute error values between the known bit score and the bit score predicted by our regression approach. In general, missing proteins are well predicted, especially in the case of the core Cas proteins Cas1– Cas7. For other Cas proteins, such as CasR, the prediction quality varies between I-A and I-E. This is likely due to the higher amount of I-E cassettes in the data basis, indicating a more complex relationship between CasR and other Cas proteins.

Fig. 5 shows the Cas protein regression results for ERT: the regressor with the best predictive performance for subtypes I-A and I-E in the dataset generated by HMM_1_. Other experimental results, for different subtypes and datasets, can be seen in Supplementary Figs S5–S9. These results show that the missing proteins are predicted with a high quality. For the core proteins Cas1 … Cas10, specifically, the proposed approach has very high prediction rates, showing a strong interdependence between these core proteins and other Cas proteins important for the subtype. We also observed that for proteins that are not core Cas proteins such as CasR, the size of the data basis (i.e., number of known cassettes for the subtype where this protein occurs) influences the prediction quality. While this is partially inherent in the ML approach, it also might indicate a more variable or complex interaction between these proteins and other proteins important for the subtype.

We also observed that, in general, ERT obtained the best results for Cas protein regression (see Supplementary Figs S5–S9). In most cases, ERT presented mean absolute error values <0.05 for the normalized bit score prediction. These results confirm the relevance of building specific regressors for each Cas protein inside of a specific subtype for the identification of unknown or possibly missing Cas proteins, when the label of the cassette of interest is known.

To assess whether the aforementioned setting would work on a more global level, we replicated the previous experiment by training the regressor on the full datasets with all subtypes. Most of the times, similar results were obtained (see Supplementary Figs S10–S14).

**Figure 6:**
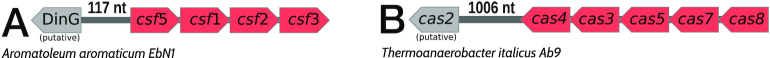
The cassettes with missing proteins. (A) In this genome, we predicted a DinG protein missing in the cassette with evidence >0.5. The HHblits [50] search in this genome for all ORFs determined 1 ORF 117 nucleotides (nt) upstream of the cassette with a high confidence score for a DinG homology (E-value: 7.6e–22). (B) In the case of Cas2, the quality of the predicted evidence was lower, between 0.221 and 0.165. Nevertheless, we found 1 ORF with a high confidence score for Cas2 homology (E-value: 1e–37) 1,006 nt downstream of the cassette.

Next, as a proof of concept, we looked at the cassettes with 1 missing protein that were left out of our experiments and constitute an independent test case (see Methods), and applied our regression approach to identify the cassettes with a high predictive performance of the evidence for having a specific missing protein. These cases would be good candidates for missing annotations. We found 13 cassettes that predicted a missing DinG protein, 3 of them with evidence of ≥0.5. By applying an HHblits [50] search for all open reading frames (ORFs) in the respective genome of these 3 cassettes, we found an ORF with convincing homology to DinG proteins in each case (see Fig. 6A for an example). Another case was Cas2, when we found 13 cassettes with a missing Cas2 protein predicted. We again used HHblits on all ORFs in the genome of the top 3 cassettes and found 1 case with a convincing Cas2 homology (see Fig. 6B).

Finally, we applied our regressors to the aforementioned test set, to predict the missing protein annotations, and the classifiers to predict the subtype for these incomplete cassettes, which were not included in the training set. Table 4 shows the classification results for all ML algorithms on this independent test. In these results, the ERT- and SVM-based classifiers, when combined with the CART regressor and the more fine-grained models HMM1 and HMM2, can predict the correct subtype with high predictive performance, even in the hard case of incomplete annotation. The ERT-based classifier can also achieve high performance when combined with the less fine-grained models HMM4 and HMM5. However, in these cases, there are fewer subtypes available because only classes containing ≥10 examples were included in our experiments (see Methods).

**Table 4.** Mean adjusted balanced accuracy for classification on the independent test set, consisting of cassettes with 1 Cas protein missing. The best results $\geq$0.7 are in bold.

Classifier	Regressor	HMM_1_	HMM_2_	HMM_3_	HMM_4_	HMM_5_
CART	–	0.50	0.50	0.68	0.48	0.48
	CART	0.68	0.68	0.52	0.55	0.55
	ERT	0.63	0.63	0.56	0.58	0.58
	SVM	0.56	0.56	0.51	0.54	0.54
ERT	–	0.63	0.63	0.65	$\mathbf {0.74}$	$\mathbf {0.74}$
	CART	$\mathbf {0.70}$	$\mathbf {0.70}$	0.63	0.64	0.63
	ERT	0.69	0.69	0.63	0.64	0.65
	SVM	0.60	0.60	0.63	0.63	0.62
SVM	–	0.50	0.50	0.58	0.64	0.64
	CART	$\mathbf {0.72}$	$\mathbf {0.72}$	0.53	0.58	0.58
	ERT	0.66	0.66	0.60	0.61	0.62
	SVM	0.54	0.54	0.53	0.57	0.57

A dash in the second column means no regression (i.e., only classification) was used.

### CRISPRcasIdentifier clearly outperforms existing tools

Finally, we assessed the quality of prediction in comparison with existing tools to assess whether they would be able to correctly classify cassettes that are not covered by the manual annotations. This is a typical application scenario, e.g., in the analysis of cassettes from metagenomic data. For this purpose, we used CRISPRcasIdentifier with default parameters and compared its performance with 3 command line CRISPR-Cas tools (CRISPRdisco [18], CRISPRcasFinder [19], and Macsyfinder [20]) and 2 webservers (CRISPRone [21] and HmmCas [22]).

To benchmark these tools we used the most recent and comprehensive set of cassettes as listed in the very recent classification article [51]. This dataset has 6,098 cassettes extracted from 4,974 archaeal and bacterial genomes, including the following subtypes: I-A to I-U, II-A to II-C, III-A to III-D, IV-A, V-A, and VI-B. In Table 5 we present the adjusted balanced accuracy scores and F-scores with the macro averaging obtained. The inferior results of HmmCas and CRISPRone can partially be explained by the fact that they (i) use the existing Cas HMMer models without any enhancement and (ii) rely on a concept that is similar to signature gene for predicting the subtype.

**Table 5.** Predictive performance of CRISPR-Cas tools for different measures

Environment	Tool	Adjusted balanced accuracy score	F-score
Webserver	CRISPRone	0.07	0.17
	HmmCas	0.05	0.15
Command line	CRISPRdisco	0.52	0.63
	CRISPRcasFinder	0.48	0.56
	Macsyfinder	0.54	0.60
	CRISPRcasIdentifier	$\mathbf {0.89}$	$\mathbf {0.91}$

Boldface indicates best results for each measure.

According to Table 5 our tool clearly outperforms the others for all measures. Our hypothesis is that the superior results are due to the generalization capability of ML models. Thus, our tool is more suitable to handle unseen examples even if they contain missing proteins. It occurs because it does not rely only on HMM profile searches but also on the general knowledge extracted from the training data. It is also important to observe that CRISPRcasIdentifier not only classifies unseen cassettes but also tries to predict potentially missing proteins, which, to the best of our knowledge, is a problem that has not been successfully addressed by the existing tools.

## Conclusion

In this article we introduced a new ML-based pipeline for the identification and classification of genomic CRISPR-Cas systems. To assess the predictive performance of this approach, we conducted an in-depth investigation into the suitability of ML algorithms that are commonly used for this task, by using the normalized profile HMM search bit scores of Cas proteins as input and classifying cassettes encoding Cas proteins to their respective subtypes according to the most recent classification [2,4,9].

Overall, this work covers 4 different research issues: (i) the classification of Cas cassettes, (ii) the prediction of normalized bit scores for missing Cas proteins, (iii) the investigation of the properties of CRISPR types and subtypes, and (iv) the comparison of our new tool to the ones available in the literature. Concerning topic (i), our classification models were able to achieve very high classification performance, >0.95, in terms of the adjusted balanced accuracy score. Thus, they are well placed for the prediction of CRISPR systems of newly sequenced organisms, or metagenomic data with sufficient read length to cover the full cassette in 1 contig. In addition, we introduced a new method for determining signature genes, which are genes most important for predicting the correct subtype. This approach was able to properly learn the known signature genes of CRISPR-Cas subtypes without any extra information other than the available gene cassettes and their labels but provides additional information about the composition of cassettes. In topic (ii), our regressor models achieved very small deviations between the expected and predicted normalized bit scores for different Cas proteins across the different subtypes. This illustrates the usefulness of these regressors on new cassettes that have missing hits for some Cas proteins. A high bit score provides a hint to researchers to search for more diverged forms of the protein or to look for proteins that could replace the missing function. The analysis performed under topic (iii) enabled us to correctly identify known signature genes and to identify putative functional modules. Overall, it provided us with a set of association rules for potential use in more advanced classification scenarios, in addition to providing insights about the biology of the systems. Finally, concerning (iv), our tool outperformed 5 other tools from the literature on the most recent and comprehensive CRISPR classification dataset published.

Manual annotation is the gold standard when it comes to classification and identification of genomic CRISPR-Cas systems. Supporting this process or annotating cassettes as part of an overall automatic pipeline such as the analysis of metagenomic data requires a classification approach with a degree of flexibility that is challenging to model. CRISPRcasIdentifier provides a boost in classification accuracy when compared to existing tools because it builds not only on an understanding of the manual annotation process but also on the generalization power of ML algorithms. We made CRISPRcasIdentifier available for researchers to use with their own data.

## Availability of Source Code and Requirements

Project name: CRISPRcasIdentifier

Project home page: https://github.com/BackofenLab/CRISPRcasIdentifier

RRID:SCR_018296

BiotoolsID: crisprcasidentifier

Operating system(s): Platform independent

Programming language: Python

Other requirements: Anaconda, Docker

License: GNU General Public License version 3 (GPLv3)

## Availability of Supporting Data and Materials

The data that support the present work are available in several publications [2–4,51]. An archival copy of the code and supporting data are also available via the *GigaScience* database, *GigaDB* [52].

## Abbreviations

BLAST: Basic Local Alignment Search Tool; CART: Classification and Regression Trees; CRISPR: clustered regularly interspaced short palindromic repeats; crRNA: CRISPR interference RNAs; ERT: Extremely Randomized Trees; HMM: hidden Markov model; MCL: Markov Cluster Algorithm; ML: machine learning; NCBI: National Center for Biotechnology Information; ORF: open reading frame; SVM: Support Vector Machines.

## Competing Interests

The authors declare that they have no competing interests.

## Funding

This research was supported by the Federal Agency for Support and Evaluation of Graduate Education within the Ministry of Education of Brazil (CAPES) (Probral CAPES/DAAD grant No. 88887.302257/2018-00), the São Paulo Research Foundation (FAPESP) (grants Nos. 2013/07375-0, 2016/18615-0, and 2019/21300-9), Intel, and the German Research Foundation (DFG) (grants BA 2168/13-1 SPP 1590 Probabilistic Structures in Evolution and BA 2168/23-1 SPP 2141 Much more than Defence: the Multiple Functions and Facets of CRISPR-Cas). The article processing charge was funded by the Baden-Wuerttemberg Ministry of Science, Research and Art and the University of Freiburg in the funding programme Open Access Publishing.

## Authors’ Contributions

V.A.P., O.S.A., A.C.P.L.F.C., and R.B. designed the study, analysed the data, and conceived the methods. V.A.P. implemented and performed the experiments. O.S.A. and S.A.S. acquired the data. V.A.P., O.S.A., S.A.S., A.C.P.L.F.C., and R.B. planned and wrote the manuscript. All authors read and approved the final manuscript.

## Supplementary Material

giaa062_GIGA-D-20-00081_Original_Submission

giaa062_GIGA-D-20-00081_Revision_1

giaa062_GIGA-D-20-00081_Revision_2

giaa062_Response_to_Reviewer_Comments_Original_Submission

giaa062_Response_to_Reviewer_Comments_Revision_1

giaa062_Reviewer_1_Report_Original_Submission Aidan O'Brien -- 4/1/2020 Reviewed

giaa062_Reviewer_2_Report_Original_Submission Florian Heigwer, Ph. D. -- 4/7/2020 Reviewed

giaa062_Supplemental_File

## References

[1] Garneau JE, Dupuis ME, Villion M, et al. The CRISPR/Cas bacterial immune system cleaves bacteriophage and plasmid DNA. Nature. 2010;468(7320):67–71. 21048762 10.1038/nature09523

[2] Makarova KS, Wolf YI, Alkhnbashi OS, et al. An updated evolutionary classification of CRISPR-Cas systems. Nat Rev Microbiol. 2015;13(11):722–36. 26411297 10.1038/nrmicro3569 PMC5426118

[3] Shmakov S, Abudayyeh OO, Makarova KS, et al. Discovery and functional characterization of diverse class 2 CRISPR-Cas systems. Mol Cell. 2015;60(3):385–97. 26593719 10.1016/j.molcel.2015.10.008 PMC4660269

[4] Shmakov S, Smargon A, Scott D, et al. Diversity and evolution of class 2 CRISPR-Cas systems. Nat Rev Microbiol. 2017;15(3):169–82. 28111461 10.1038/nrmicro.2016.184 PMC5851899

[5] Cass SDB, Haas KA, Stoll B, et al. The role of Cas8 in type I CRISPR interference. Biosci Rep. 2015;35(4):e00197. 26182359 10.1042/BSR20150043 PMC4613674

[6] Sinkunas T, Gasiunas G, Fremaux C, et al. Cas3 is a single-stranded DNA nuclease and ATP-dependent helicase in the CRISPR/Cas immune system. EMBO J. 2011;30(7):1335–42. 21343909 10.1038/emboj.2011.41 PMC3094125

[7] Zhang J, Rouillon C, Kerou M, et al. Structure and mechanism of the CMR complex for CRISPR-mediated antiviral immunity. Mol Cell. 2012;45(3):303–13. 22227115 10.1016/j.molcel.2011.12.013 PMC3381847

[8] Deng L, Kenchappa CS, Peng X, et al. Modulation of CRISPR locus transcription by the repeat-binding protein Cbp1 in *Sulfolobus*. Nucleic Acids Res. 2012;40(6):2470–80. 22139923 10.1093/nar/gkr1111 PMC3315313

[9] Shah SA, Alkhnbashi OS, Behler J, et al. Comprehensive search for accessory proteins encoded with archaeal and bacterial type III CRISPR-Cas gene cassettes reveals 39 new Cas gene families. RNA Biol. 2019;16(4):530–42. 29911924 10.1080/15476286.2018.1483685 PMC6546367

[10] Haft DH, Selengut J, Mongodin EF, et al. A guild of 45 CRISPR-associated (Cas) protein families and multiple CRISPR/Cas subtypes exist in prokaryotic genomes. PLoS Comput Biol. 2005;1(6):e60. 16292354 10.1371/journal.pcbi.0010060 PMC1282333

[11] Makarova KS, Haft DH, Barrangou R, et al. Evolution and classification of the CRISPR-Cas systems. Nat Rev Microbiol. 2011;9(6):467–77. 21552286 10.1038/nrmicro2577 PMC3380444

[12] Marchler-Bauer A, Bryant SH CD-Search: Protein domain annotations on the fly. Nucleic Acids Res. 2004;32(suppl 2):W327–31. 15215404 10.1093/nar/gkh454 PMC441592

[13] Lange SJ, Alkhnbashi OS, Rose D, et al. CRISPRmap: An automated classification of repeat conservation in prokaryotic adaptive immune systems. Nucleic Acids Res. 2013;41(17):8034–44. 23863837 10.1093/nar/gkt606 PMC3783184

[14] Alkhnbashi OS, Costa F, Shah SA, et al. CRISPRstrand: Predicting repeat orientations to determine the crRNA-encoding strand at CRISPR loci. Bioinformatics. 2014;30(17):i489–96. 25161238 10.1093/bioinformatics/btu459 PMC4147912

[15] Biswas A, Staals RHJ, Morales SE, et al. CRISPRDetect: A flexible algorithm to define CRISPR arrays. BMC Genomics. 2016;17:356. 27184979 10.1186/s12864-016-2627-0 PMC4869251

[16] Alkhnbashi OS, Shah SA, Garrett RA, et al. Characterizing leader sequences of CRISPR loci. Bioinformatics. 2016;32(17):i576–85. 27587677 10.1093/bioinformatics/btw454

[17] Finn RD, Clements J, Eddy SR HMMER web server: Interactive sequence similarity searching. Nucleic Acids Res. 2011;39(suppl 2):W29–W37. 21593126 10.1093/nar/gkr367 PMC3125773

[18] Crawley AB, Henriksen JR, Barrangou R, et al. CRISPRdisco: An automated pipeline for the discovery and analysis of CRISPR-Cas systems. CRISPR J. 2018;1(2):171–81. 31021201 10.1089/crispr.2017.0022 PMC6636876

[19] Couvin D, Bernheim A, Toffano-Nioche C, et al. CRISPRCasFinder, an update of CRISRFinder, includes a portable version, enhanced performance and integrates search for Cas proteins. Nucleic Acids Res. 2018;46(W1):W246–51. 29790974 10.1093/nar/gky425 PMC6030898

[20] Abby SS, Néron B, Ménager H, et al. MacSyFinder: A program to mine genomes for molecular systems with an application to CRISPR-Cas systems. PLoS One. 2014;9(10):e110726. 25330359 10.1371/journal.pone.0110726 PMC4201578

[21] Zhang Q, Ye Y Not all predicted CRISPR-Cas systems are equal: isolated Cas genes and classes of CRISPR like elements. BMC Bioinformatics. 2017;18(1):92. 28166719 10.1186/s12859-017-1512-4 PMC5294841

[22] Chai G, Yu M, Jiang L, et al. HMMCAS: A web tool for the identification and domain annotations of Cas proteins. IEEE/ACM Trans Comput Biol Bioinform. 2019;16(4):1313–5. 28186905 10.1109/TCBB.2017.2665542

[23] Pearson WR, Lipman DJ Improved tools for biological sequence comparison. Proc Natl Acad Sci U S A. 1988;85(8):2444–8. 3162770 10.1073/pnas.85.8.2444 PMC280013

[24] Enright AJ, Van Dongen S, Ouzounis CA, et al. An efficient algorithm for large-scale detection of protein families. Nucleic Acids Res. 2002;30(7):1575–84. 11917018 10.1093/nar/30.7.1575 PMC101833

[25] Edgar RC MUSCLE: Multiple sequence alignment with high accuracy and high throughput. Nucleic Acids Res. 2004;32:1792–7. 15034147 10.1093/nar/gkh340 PMC390337

[26] Hyatt D, Chen GL, LoCascio PF, et al. Prodigal: Prokaryotic gene recognition and translation initiation site identification. BMC Bioinformatics. 2010;11:119. 20211023 10.1186/1471-2105-11-119 PMC2848648

[27] Breiman L, Friedman JH, Olshen RA, et al. Classification and Regression Trees. Chapman & Hall/CRC; 1984.

[28] Vapnik V The Nature of Statistical Learning Theory. Springer; 1995.

[29] Geurts P, Ernst D, Wehenkel L, et al. Extremely randomized trees. Mach Learn. 2006;63(1):3–42.

[30] Wu X, Kumar V, Quinlan JR, et al. Top 10 algorithms in data mining. Knowl Inf Syst. 2008;14(1):1–37.

[31] Varma S, Simon R Bias in error estimation when using cross-validation for model selection. BMC Bioinformatics. 2006;7(1):91. 16504092 10.1186/1471-2105-7-91 PMC1397873

[32] Cawley GC, Talbot NL On over-fitting in model selection and subsequent selection bias in performance evaluation. J Mach Learn Res. 2010;11:2079–107.

[33] Krstajic D, Buturovic LJ, Leahy DE, et al. Cross-validation pitfalls when selecting and assessing regression and classification models. J Cheminformatics. 2014;6(1):10. 10.1186/1758-2946-6-10 PMC3994246 24678909

[34] Bishop CM Pattern Recognition and Machine Learning. Springer; 2006.

[35] Hastie T, Tibshirani R, Friedman J The Elements of Statistical Learning: Data Mining, Inference and Prediction. 2nd ed. Springer; 2009.

[36] Forman G, Scholz M Apples-to-apples in cross-validation studies: Pitfalls in classifier performance measurement. ACM SIGKDD Explor Newsl. 2010;12(1):49–57.

[37] Brodersen KH, Ong CS, Stephan KE, et al. The balanced accuracy and its posterior distribution. In: 2010 20th International Conference on Pattern Recognition, Istanbul. IEEE; 2010:3121–4.

[38] Guyon I, Bennett K, Cawley G, et al. Design of the 2015 chalearn automl challenge. In: 2015 International Joint Conference on Neural Networks (IJCNN). IEEE; 2015:1–8.

[39] Sokolova M, Lapalme G A systematic analysis of performance measures for classification tasks. Inf Proc Manag. 2009;45(4):427–37.

[40] Willmott CJ, Matsuura K Advantages of the mean absolute error (MAE) over the root mean square error (RMSE) in assessing average model performance. Clim Res. 2005;30(1):79–82.

[41] Pedregosa F, Varoquaux G, Gramfort A, et al. Scikit-learn: Machine learning in Python. J Mach Learn Res. 2011;12:2825–30.

[42] Hsu CW, Chang CC, Lin CJ, et al. A practical guide to support vector classification. 2003, https://www.csie.ntu.edu.tw/~cjlin/papers/guide/guide.pdf.

[43] Cherkassky V, Dhar S Simple method for interpretation of high-dimensional nonlinear SVM classification models. In: 6th International Conference on Data Mining. 2010:267–272.

[44] Nam KH, Kurinov I, Ke A, et al. Crystal structure of clustered regularly interspaced short palindromic repeats (CRISPR)-associated Csn2 protein revealed Ca2+-dependent double-stranded DNA binding activity. J Biol Chem. 2011;286(35):30759–68. 21697083 10.1074/jbc.M111.256263 PMC3162437

[45] Koo Y, Jung DK, Bae E, et al. Crystal structure of *Streptococcus pyogenes* Csn2 reveals calcium-dependent conformational changes in its tertiary and quaternary structure. PLoS One. 2012;7(3):1–8. 10.1371/journal.pone.0033401 PMC3316568 22479393

[46] Arslan Z, Wurm W, Brener O, et al. Double-strand DNA end-binding and sliding of the toroidal CRISPR-associated protein Csn2. Nucleic Acids Res. 2013;41(12):6347–59. 23625968 10.1093/nar/gkt315 PMC3695520

[47] Lee KH, Lee SG, Lee KE, et al. Identification, structural, and biochemical characterization of a group of large Csn2 proteins involved in CRISPR-mediated bacterial immunity. Proteins. 2012;80(11):2573–82. 22753072 10.1002/prot.24138

[48] He F, Vestergaard G, Peng W, et al. CRISPR-Cas type I-A Cascade complex couples viral infection surveillance to host transcriptional regulation in the dependence of Csa3b. Nucleic Acids Res. 2017;45(4):1902–13. 27980065 10.1093/nar/gkw1265 PMC5389559

[49] Vestergaard G, Garrett RA, Shah SA CRISPR adaptive immune systems of Archaea. RNA Biol. 2014;11(2):156–67. 24531374 10.4161/rna.27990 PMC3973734

[50] Remmert M, Biegert A, Hauser A, et al. HHblits: Lightning-fast iterative protein sequence searching by HMM-HMM alignment. Nat Methods. 2011;9(2):173–5. 22198341 10.1038/nmeth.1818

[51] Makarova KS, Wolf YI, Iranzo J, et al. Evolutionary classification of CRISPR–Cas systems: A burst of class 2 and derived variants. Nat Rev Microbiol. 2020;18(2):67–83. 31857715 10.1038/s41579-019-0299-x PMC8905525

[52] Padilha VA, Alkhnbashi OS, Shah SA, et al. Supporting data for “CRISPRcasIdentifier: Machine learning for accurate identification and classification of CRISPR-Cas systems.”. GigaScience Database. 2020; 10.5524/100751. PMC7298778 32556168

