# Supplementary material for: CRISPRcasIdentifier: Machine learning for accurate identification and classification of CRISPR-Cas systems
Source: Gigascience. 2020 Jun 17;9(6):giaa062. doi: 10.1093/gigascience/giaa062 (PMC7298778; doi:10.1093/gigascience/giaa062)
Supplement: giaa062_GIGA-D-20-00081_Revision_1 [file giaa062_giga-d-20-00081_revision_1.pdf]

GigaScience

CRISPRcasIdentifier: Machine learning for accurate identification and classification of CRISPR-Cas systems

--Manuscript Draft--

**Manuscript Number:**	GIGA-D-20-00081R1
**Full Title:**	CRISPRcasIdentifier: Machine learning for accurate identification and classification of CRISPR-Cas systems
**Article Type:**	Research
**Funding Information:**	Coordenação de Aperfeiçoamento de Pessoal de Nível Superior (Probral 88887.302257/2018-00) — Mr Victor A Padilha<br>Fundação de Amparo à Pesquisa do Estado de São Paulo (2019/21300-9) — Mr Victor A Padilha<br>Fundação de Amparo à Pesquisa do Estado de São Paulo (2016/18615-0) — Dr André C P L F Carvalho<br>Fundação de Amparo à Pesquisa do Estado de São Paulo (2013/07375-0) — Not applicable<br>Deutsche Forschungsgemeinschaft (BA 2168/13-1 SPP 1590) — Dr Rolf Backofen<br>Deutsche Forschungsgemeinschaft (BA 2168/23-1 SPP 2141) — Dr Rolf Backofen
**Abstract:**	Background: CRISPR-Cas genes are extraordinarily diverse and evolve rapidly when compared to other prokaryotic genes. With the rapid increase in newly sequenced archaeal and bacterial genomes, manual identification of CRISPR-Cas systems is no longer viable. Thus, an automated approach is required for advancing our understanding of the evolution and diversity of these systems, and for finding new candidates for genome engineering in eukaryotic models. Results: We introduce CRISPRcasIdentifier, a new machine learning based tool that combines regression and classification models<br>for the prediction of potentially missing proteins in instances of CRISPR-Cas systems and the prediction of their respective subtypes. In contrast to other available tools, CRISPRcasIdentifier can both detect cas genes and extract potential association rules that reveal functional modules for CRISPR-Cas systems. In our experimental benchmark on the most recently published and comprehensive CRISPR-Cas system dataset, CRISPRcasIdentifier was compared with recent and state-of-the-art tools. According to the experimental results, CRISPRcasIdentifier presented the best Cas protein identification and subtype classification performance. Conclusions: Overall, our tool greatly extends the classification of CRISPR cassettes and, for the first time, predicts missing Cas proteins and association rules between Cas proteins. Additionally, we investigated the properties of CRISPR subtypes. The proposed tool relies not only on the knowledge of manual CRISPR annotation but also on models trained by using machine learning.
**Corresponding Author:**	Omer Alkhnbashi<br><br>GERMANY
**Corresponding Author Secondary Information:**	
**Corresponding Author's Institution:**	
**Corresponding Author's Secondary Institution:**	
**First Author:**	Victor A Padilha
**First Author Secondary Information:**	

Powered by Editorial Manager® and ProduXion Manager® from Aries Systems Corporation

**Order of Authors:**	Victor A Padilha
	Omer Alkhnbashi
	Shiraz A Shah
	André C P L F Carvalho
	Rolf Backofen
**Order of Authors Secondary Information:**	
**Response to Reviewers:**	GigaScience<br><br>Dear Dr. Hans Zauner<br><br>Herewith we submit the revised version of our manuscript<br><br>CRISPRcasIdentifier: Machine learning for accurate identification and classification of CRISPR-Cas systems<br><br><br>We have answered each issue and have modified our submission where necessary. We have taken careful consideration of the reviewers’ comments and have integrated them into a thoroughly revised version of the manuscript. Additional to the reviewers comments, we took your suggestion into consideration. We are grateful for the constructive comments that have significantly improved the presentation of our work.<br><br><br>Please find a point-by-point response to the reviews below.<br><br><br>I am looking forward to hearing from you.<br><br><br>Sincerely yours,<br>Prof. Dr. Rolf Backofen<br><br><br><br><br>--<br><br><br><br><br><br>Point-to-point response to reviewer comments:<br><br><br><br>Editor suggestion<br><br>1- I particularly agree with reviewer 1 that clarifying all data sources and improving the "readme" are two important points to address. Please also consider the suggestion of reviewer 2 to provide a dockerized version for end users' convenience. In addition, please register any new software application in the bio.tools and SciCrunch.org databases to receive RRID (Research Resource Identification Initiative ID) and biotoolsID identifiers, and include these in your manuscript. This will facilitate tracking, reproducibility and re-use of your tool<br><br>Authors’ response: First, we better clarified the data sources by providing one extra table in our GitHub repository (https://github.com/BackofenLab/CRISPRcasIdentifier, see Supplementary_files folder under the GitHub) which lists all CRISPR cassettes

Powered by Editorial Manager® and ProduXion Manager® from Aries Systems Corporation

used and from which source each one of them came from. In addition, our repository includes a detailed README.md file (which describes the installation process and usage) and we also created a dockerized version (https://hub.docker.com/repository/docker/padilha/crispr-cas-identifier), as suggested by reviewer #2. Finally, we also registered our tool in SciCrunch.org (RRID: SCR_018296) and bio.tools (biotoolsID: crisprcasidentifier) and included the respective IDs in the “Availability of Source Code and Requirements” section.

Reviewer #1: The computational tool that the authors present for identifying CRISPR-Cas systems can potentially save researchers time and effort as well as assisting in identifying novel Cas proteins. Although not the first tool in this space, CRISPRCasIdentifier does appear to outperform pre-existing tools by a large margin. Modelling is based on a good-sized dataset and the ML component appropriately features cross-validation and tuning. The paper is well-written with minimal grammatical errors.

Manuscript comments:
1- I understand that the dataset appears to be from multiple sources with a total of 68594 samples. Is this the data from [2,3,4]? A table (perhaps in supplementary) outlining how much and from where all of the data comes from would help clarify this.

Authors’ response: Done. Please see the supplementary file named CRISPR_cassette_source.tab in our GitHub repository (https://github.com/BackofenLab/CRISPRcasIdentifier).

2 - Although both terms are appropriate, "trained" seems to be more common vocabulary than "induced" based on a quick literature search of recent manuscripts. I don't believe all instances of induced in the manuscript must be replaced with trained, but I do believe trained sounds more natural so should perhaps be considered. I.e. in the abstract "models induced by using machine learning" compared with "models trained using machine learning".

Authors’ response: Thank you for the suggestion, we changed all occurrences of the word “induce” to “train” in the text.

3 - Please check the grammar of the following phrase in "Data collecting and preprocessing" section: "is a pair of the genomic sequence containing all genes of the cassette".

Authors’ response: We checked the grammar and corrected the sentence.

4 - Should the title of that section be "Data collection and preprocessing"?

Authors’ response: We agree with the reviewer and changed the title of the section.

5 - Is hyphenation intended in this sentence? "and as a result re-assure key components of subtypes like"

Authors’ response: The correct term is reassure. We corrected it in the text.

6- I notice the project web page is password-protected, although the password is provided in the manuscript. Is password-protection a temporary measure until the paper is accepted?
I believe it would be more user-friendly to have the project available as a GitHub repository with README.md available upfront, rather then requiring the user to follow one set of instructions before being able to view it.
Also, as I'm sure you know, this has the benefit of tagged releases for better reproducibility, given the inevitable bugfixes/improvements of bioinformatics tools.

Authors’ response: Yes, the password protection was a temporary measure for the review process. We moved the code to a public GitHub repository. It is now available with a proper README.md file in: https://github.com/BackofenLab/CRISPRcasIdentifier. We also created a tagged release for the version we used in our paper.

7- Program comments:
* Biopython should be listed as a requirement in README.md
* The examples in README.md on lines 78 and 79 contain "fasta" extensions butthe respective files in the "examples" directory have a "fa" extension.
* The program looks for "models" directory or "models.tar.gz" archive. If I follow theinstructions I end up with a "trained_models_2015" directory. Therefore the program fails unless this directory is renamed.

Authors’ response: We thank the reviewer for all the above comments. We corrected them as suggested.

Reviewer #2:

The manuscript is well written and the conclusion is backed up by a comprehensive banchmark and data data collection. The tools are available and the code is documented.

My only concern is that my Mac had a hard time resolving the conda environment. More testing of the boiler plate code to allow others to deploy your methods is warranted prior to publication. (a docker container based solution through docker hub might be quick to implement and make things easier for end-users)

Despite this, I think the manuscript describes an elegant solution to a timely problem that may likely help many researches to find and understand CRISPR systems in more organisms.

Authors’ response: We would like to thank the reviewer for this comment. We now provide the users with three options to install our tool:
They may use a DockerHub image, available in https://hub.docker.com/repository/docker/padilha/crispr-cas-identifier;
They may build our docker image from the Dockerfile available in our GitHub repository (https://github.com/BackofenLab/CRISPRcasIdentifier); or
They may set a conda environment up from our crispr-env.yml file, also available in our Github repository.

In the README.md file we explain each of the aforementioned options in detail.

**Additional Information:**	
**Question**	**Response**
Are you submitting this manuscript to a special series or article collection?	No
**Experimental design and statistics**<br><br>Full details of the experimental design and statistical methods used should be given in the Methods section, as detailed in our	Yes

Minimum Standards Reporting Checklist. Information essential to interpreting the data presented should be made available in the figure legends.<br><br>Have you included all the information requested in your manuscript?	
**Resources**<br><br>A description of all resources used, including antibodies, cell lines, animals and software tools, with enough information to allow them to be uniquely identified, should be included in the Methods section. Authors are strongly encouraged to cite Research Resource Identifiers (RRIDs) for antibodies, model organisms and tools, where possible.<br><br>Have you included the information requested as detailed in our Minimum Standards Reporting Checklist?	Yes
**Availability of data and materials**<br><br>All datasets and code on which the conclusions of the paper rely must be either included in your submission or deposited in publicly available repositories (where available and ethically appropriate), referencing such data using a unique identifier in the references and in the “Availability of Data and Materials” section of your manuscript.<br><br>Have you have met the above requirement as detailed in our Minimum Standards Reporting Checklist?	Yes

Manuscript
Click here to access/download;Manuscript;CRISPRcasIdentifier_manuscript

OXFORD
(GIGA)^n SCIENCE

GigaScience, 2017, 1–11

doi: xx.xxxx/xxxx
Manuscript in Preparation
Paper

PAPER

CRISPRcasIdentifier: Machine learning for accurate identification and classification of CRISPR-Cas systems

Victor A. Padilha[1,†], Omer S. Alkhnbashi[2,†], Shiraz A. Shah[3], André C. P. L. F. de Carvalho[1] and Rolf Backofen[2,4,*]

[1]Institute of Mathematics and Computer Sciences, University of São Paulo, Av. Trabalhador São Carlense 400, São Carlos, SP, 13564-002, Brazil and [2]Chair of Bioinformatics, University of Freiburg, Georges-Köhler-Allee 101, Freiburg, 79110, Germany and [3]Danish Archaea Centre, Department of Biology, University of Copenhagen, Ole Maaløes Vej 5, Copenhagen, DK-2200, Denmark and [4]Signalling Research Centres BIOSS and CIBSS, University of Freiburg, Georges-Köhler-Allee 101, Freiburg, 79110, Germany

[*]backofen@informatik.uni-freiburg.de
[†]Contributed equally as first authors.

Abstract

Background: CRISPR-Cas genes are extraordinarily diverse and evolve rapidly when compared to other prokaryotic genes. With the rapid increase in newly sequenced archaeal and bacterial genomes, manual identification of CRISPR-Cas systems is no longer viable. Thus, an automated approach is required for advancing our understanding of the evolution and diversity of these systems, and for finding new candidates for genome engineering in eukaryotic models. **Results:** We introduce CRISPRcasIdentifier, a new machine learning based tool that combines regression and classification models for the prediction of potentially missing proteins in instances of CRISPR-Cas systems and the prediction of their respective subtypes. In contrast to other available tools, CRISPRcasIdentifier can both detect *cas* genes and extract potential association rules that reveal functional modules for CRISPR-Cas systems. In our experimental benchmark on the most recently published and comprehensive CRISPR-Cas system dataset, CRISPRcasIdentifier was compared with recent and state-of-the-art tools. According to the experimental results, CRISPRcasIdentifier presented the best Cas protein identification and subtype classification performance. **Conclusions:** Overall, our tool greatly extends the classification of CRISPR cassettes and, for the first time, predicts missing Cas proteins and association rules between Cas proteins. Additionally, we investigated the properties of CRISPR subtypes. The proposed tool relies not only on the knowledge of manual CRISPR annotation but also on models trained using machine learning.

Key words: CRISPR-Cas; Machine Learning; Cas genes; Cas proteins.

Background

CRISPR-Cas systems provide archaea and bacteria with a nucleic acid based adaptive immune system against invading viruses and plasmids. Mechanistically, the immune response can be divided into three stages, namely adaptation, processing and interference, each carried out by different sets of protein complexes [1]. The universally conserved proteins Cas1, Cas2 and, optionally Cas4, are responsible for the adaptation stage, when a fragment of invader DNA is excised and stored in the host chromosome as a spacer in the non-coding CRISPR region. The processing and interference stages are much more mech-

Compiled on: April 27, 2020.
Draft manuscript prepared by the author.

anistically diverse, using different sets of proteins, depending on the type of CRISPR-Cas system. CRISPR-Cas systems are found in many bacteria and most archaea, and have diversified as much as their host organisms [2].

While the mechanistic principles are similar, with spacers comprising templates for synthesis of CRISPR interference RNAs (crRNAs) against the invader, the different types and classes of CRISPR-Cas systems show some important differences. Class 2 systems use a single multi-domain protein for locating and cleaving the re-invading nucleic acid, whereas Class 1 systems employ a large multi-subunit complex for the same purpose. Class 2 systems can be further subdivided into type II, V and VI, which appear to have evolved independently from each other. Thus, the respective Cas9, 12 and 13 enzymes that carry out invader cleavage rely on diverse mechanisms involving differing nuclease domains [2, 3, 4].

Class 1 systems, on the other hand, with types I, III and IV, use structurally related proteins to carry out similar functions, although the protein subunits have diverged considerably. Common to all Class 1 systems is that Cas7 forms a helical backbone that spans the length of the tightly bound crRNA. This backbone is terminated in one end by Cas5, which itself is bound to Cas8 or Cas10 for type I and IV, or type III systems, respectively. Type I systems use Cas8 for the recognition of the protospacer adjacent motif (PAM) [5], which, along with invader crRNA hybridisation, comprises a signal for recruitment of the Cas3 helicase-nuclease protein that subsequently digests the invader chromosome [6]. Type III systems, however, use the Cas7 backbone for cleaving invader mRNA while the Cas10 HD nuclease cleaves transcribed DNA [7, 8]. Cas10 also synthesises a signaling molecule that recruits additional accessory Cas proteins for other functions, such as cell suicide or activation of other defense systems [8, 9].

The different types of CRISPR-Cas systems are themselves so diverse that each type can be further subdivided into several subtypes. Type III, for example, is divided into four subtypes III-A, B, C and D. While CRISPR-Cas systems of the same subtype encode similar proteins that occupy the same roles, the proteins have often diverged beyond the point of recognition by conventional sequence alignment methods such as BLAST, even within a subtype. This level of sequence diversity makes proper identification of the found CRISPR-Cas systems very challenging, and the field has thus far relied upon the gold standard of periodic manual annotations by experts, published once every few years [10, 11, 2]. The annotation involves profile HMM searches for finding core genes, followed by the inspection of their neighborhoods, gauging operonic structures, and manual BLAST and PSI-BLAST searches [12]. With the increasing number of genome sequences from uncultured microbes and metagenomic data, however, manual annotations cannot keep up and an automated approach is needed, which yields comparable accuracy to manual annotation. Furthermore, research groups working on organisms not yet covered by published annotations have thus far made their own manual annotations, leading to inconsistencies in nomenclature and inaccuracies in some cases.

There have been numerous attempts at devising computational pipelines for the identification of different elements of the CRISPR system, such as CRISPR arrays [13, 14, 15] and CRISPR leaders [16]. On the other hand, command line tools and webservers, usually based on Hidden Markov Models (HMM) and HMMER [17] or PSI-BLAST [12], were proposed for CRISPR subtype prediction. Examples of such tools are: CRISPRdisco [18], CRISPRcasFinder [19], Macsyfinder [20], CRISPRone [21] and HmmCas [22]. We found, however, that the existing tools usually lack the ability to generalize unseen examples. Additionally, these tools are neither able to adapt to an extending repertoire of *cas* genes, nor predict possibly missing proteins, nor can learn association rules among proteins.

In this work, we present a machine learning (ML) approach intending to capture much of the relevant essence of manual annotation. It is based on evidence for the different Cas proteins to be contained in a series of consecutive genes that are part of a cas cassette, and thus represents genomic CRISPR-Cas systems as cassettes of adjacently encoded proteins. These evidences are calculated by newly designed sets of HMM models for each Cas protein, covering the diversity of Cas protein families. The proposed approach solves the problem of classification of new systems into types and subtypes. As our features for the ML approach correspond to evidence for Cas proteins, we can determine Cas proteins whose evidence is critical for predicting a subtype, which corresponds to the concept of signature genes. We show that our approach correctly identifies known signature genes for types and subtypes. In addition, our approach is able to provide more information about the composition of cassettes. One application is to predict evidence for Cas proteins that have been missed in the Cas protein screening. This provides researchers with hints to search for remote homologs of the missing Cas proteins, or for new proteins that might replace the associated function. Furthermore, we are able to learn association rules, which are subsets of proteins being important to each other, indicating functional modules. As a proof of concept, when we search for Cas proteins associated with an interference protein, our approach finds other interference proteins to be most important. The more interesting cases are undoubtedly with non-interference proteins, where our tool could correctly predict a strong association of the ancillary protein Csn2 with Cas1, consistent with its hypothesized role in adaptation. For the protein CasR we found that it is associated with different functional modules in subtypes I-A and I-E, indicating a possible functional diversity. Thus, the set of protein associations derived in this manner provides a proper resource for researchers that want to investigate the function of different Cas proteins.

Methods

Data collection and preprocessing

All Cas proteins used in this study were selected from the current classified archaeal and bacterial CRISPR-Cas systems [2, 3, 4]. We performed an all-against-all sequence similarity comparison on these data using Fasta [23]. Subsequently, we clustered the proteins using the Markov Cluster Algorithm (MCL) [24] based on custom similarity criteria [16, 9]. These criteria consider the size of the proteins, the length alignment and the relative locations of similar regions between the two compared proteins. After clustering the protein sequences from a specific Cas protein family, we generated a multiple sequence alignment (MSA) using MUSCLE [25]. Next, these alignments were converted to HMM profile models by using hmmbuild [17]. Except for MCL, all other tools were run with default parameters.

Throughout the text, each cassette in our training and test data sets is represented by a tuple consisting of its genomic sequence containing all genes of the cassette, and the list of all annotated Cas proteins. We extracted the genomic sequences as follows: we took the Supplementary Table S7 from [2], which contains all gene loci (i.e. genomic positions) in column "(sub)Type / Coordinates", and downloaded the sequences from NCBI. For the second part, namely the list of all Cas proteins, we extracted the genomic sequence for each annotated Cas protein individually, again from the "coordinates" column and added 50 bases of context. The associated amino-acid sequences were generated by running the Prodigal tool

v2.6.3 [26] on the respective gene sequences, and stored together with the Cas annotation from the column "*cas* gene" in Table S7 from [2].

To generate the feature vectors, we ran all HMM profile models using hmmsearch against the sequences of all cassettes. We selected the cassettes, which had a hit for all proteins annotated for that subtype, and used this as training and test set for the classification pipeline. Cassettes that had a missing protein were used instead as an independent test case for our regression models and the full pipeline.

Classification of Cas cassettes

For this task, we apply ML algorithms onto a finite sample of CRISPR data in order to obtain predictive models that are able to classify Cas cassettes into their respective subtypes using a data matrix representation (see Results and discussion). Thus, based on the finite sample of data, we investigate the application of classification algorithms that estimate a function which is able to generalize the association between a cassette and its subtype. As a consequence, we intend to use this function to classify new cassettes that were not seen during the training phase into their respective subtypes with a high level of accuracy.

Prediction of missing Cas proteins

We also investigate the problem of predicting (possibly) missing Cas proteins by estimating their normalized bit scores. For this problem, we modelled it as follows. Given m Cas proteins, we filter, for each subtype, its set of $l < m$ proteins (i.e., all Cas proteins whose bit score is larger than zero for at least one cassette of the subtype). Next, we train l regressors, where the jth regressor, $j \in \{1, \cdots, l\}$, predicts the bit score of the jth Cas protein using the remaining $l - 1$ proteins as input.

Experimental evaluation of ML algorithms

Three ML algorithms were applied to the preprocessed dataset to train classification and regression models:

- *Classification and Regression Trees (CART)* [27], which trains a predictive model represented by a decision tree. This algorithm can train decision trees for classification (classification trees) and regression (regression trees) tasks. A decision tree is composed by a set of interpretable rules extracted from the training dataset. These rules explain the decisions made by the model to predict the class or regression value for new, previously unseen, examples.
- *Support Vector Machines (SVM)* [28], which trains a binary classifier represented by a hyperplane that separates examples from two classes with the maximum possible separation margin. By using kernel functions, an SVM can be applied to non-linearly separable problems. For multiclass classification tasks, a multiclass dataset is usually first decomposed into several classification binary datasets. SVMs can then be applied to each binary dataset, and their predictions are combined for a multiclasss classification.
- *Extremely Randomized Trees (ERT)* [29], uses an ensemble of decision trees, where each tree is trained using a random subset of the original features. Instead of selecting the best discriminating threshold for each feature considered for a split, as would be the case for classical decision trees, ERT chooses a random threshold value. The final predictions are the average of the predictions of all the decision trees in the ensemble. We can extract the importance of each feature in the classification or regression task from the decision trees in the ensemble. The importance is represented by the decrease in impurity caused by a node that splits the feature, weighted by the number of examples contained in such a node [30], and averaged over all trees of the ensemble.

The model selection and evaluation of predictive models is a widely studied problem in the ML literature. Several works, such as [31, 32, 33], investigate the advantages and drawbacks of different methodologies. Based on these previous studies, we use the nested cross-validation procedure. Given a set of data, the classical cross-validation approach splits the data into K mutually exclusive and similar sized subsets called folds. Next, at each iteration, $K - 1$ folds are used for training an ML model and the remaining fold for testing it [34, 35]. The nested cross-validation approach separates the model selection and evaluation steps, by using two different cross-validation loops: an outer loop, which splits the data into K_1 folds, and is used for model evaluation; and an inner loop, which splits the training data into K_2 folds, and is used for model selection. In this paper, we set $K_1 = K_2 = 10$, and repeat the evaluation procedure 50 times, due to the variance of the results when considering different splits [33]. It is important to mention that, during our experiments, to guarantee that examples from all classes are present in each outer fold, we used only classes containing at least 10 examples.

For each cross-validation iteration, we aggregate the predictions from all folds and calculate a single predictive performance evaluation, in order to avoid any averaging problems that might arise, especially when the dataset is imbalanced [36]. For the classification experiments, we used the following evaluation measures: adjusted balanced accuracy score [37, 38], an adaptation of the original accuracy measure that gives higher weights to examples from smaller classes; and the F-score with macro-averaging [39], which is the average F-score among all classes. Both measures treat different subtypes equally. Thus, they do not favor those with the largest numbers of cassettes. For the regression experiments, we used the mean absolute error [40], which is the average absolute difference between the expected and the predicted target values.

Regarding the model selection step of each ML algorithm used, we performed a grid search over 20 different hyperparameter combinations, based on the guidelines from the scikit-learn package [41]. We describe these hyperparameter grids next. For the CART algorithm, we varied the hyperparameters that determine the maximum depth of the decision tree and the minimum number of examples necessary for a node to become a leaf. For the former, we considered the values in $\{5, 10, 15, \max\}$, where max allows the tree to grow as deep as possible. For the latter, we varied the values in $\{5, 6, 7, 8, 9\}$. For the SVM algorithm, we used a Gaussian kernel, due to its ability to model nonlinear decision boundaries and its reduced number of hyperparameters when compared with another commonly used nonlinear kernel, the polynomial kernel [42]. For the cost hyperparameter C, we considered the values in $\{1, 10, 100, 1000\}$. Regarding the kernel coefficient γ, we assessed the values in $\{0.01, 0.1, 1, 10, 100\}$. Finally, for the ERT algorithm, we varied the ensemble size using the values in $\{25, 50, 75, 100\}$, and the quantity of features to be considered when performing a split from the set of values in $\{25\%, 50\%, 75\%, 100\%, \sqrt{m}\}$, where m is the number of known Cas protein families.

Results and discussion

A combined approach to determine Cas proteins and cassette subtypes

The classification of a subtype is based on the membership for specific Cas proteins. Thus, any ML-based classification of a cassette requires the detection of the contained Cas proteins as a first step. While this first step is commonly performed using Hidden Markov models, a difficulty arises from the fact that a single Cas protein family has to be split into different subfamilies due to the high evolutionary diversity of their members. Due to missing values in the dataset for a family, even the problem of splitting into different subfamilies is not an easy one. Even further, we have observed that the splitting of Cas protein families influence the quality of ML-based subtype classification. This would be quite obvious if subfamilies of individual Cas protein would correlate well with subtypes. The real situation, however, is more complex, partially due to the fact that cassettes are composed in a modular way, often involving horizontal gene transfer [2, 9].

In brief and as described in more detail later, our classification approach takes the bit scores for the contained Cas proteins as evidence of their membership to the cassette. We use this information to apply a set of ML algorithms to classify the subtypes of cassettes. By generating different divisions of subfamilies for each Cas protein, we obtain different evidences for the contained Cas proteins. Thus, we can investigate which division is best related to subtype evolution. With this holistic view of Cas protein and subtype annotation, we can further examine relations between subtypes and Cas protein membership and as a result reassure key components of subtypes like signature genes.

Detection of Cas proteins by families of HMMer models

Our definition of Cas protein subfamilies is based on clustering the known sequences of a specific Cas protein family. We use around 68594 Cas proteins as a database, and applied different cluster criterion. Each cluster characterizes a subfamily, which is afterwards represented by a HMM model. All models for a Cas protein are grouped, and the best matching HMM for each Cas protein is used to score a new sequence. To cluster the sequences, we performed an all-against-all sequence similarity comparison. Subsequently, we applied the Markov Cluster Algorithm (MCL) [24] to cluster the known sequences for a specific Cas protein family according to their sequence similarities. However, protein sequences can be clustered in different ways, depending on the cut-off for sequence similarity and the requested coverage of the alignment between two sequences. In addition, different hyperparameters for the MCL clustering algorithm result in different data partitions. Each partition defines different subfamilies, for which we train HMM models.

The different clustering approaches thus result in HMM models for different subfamilies, with varying specificity and sensitivity to detect members of a Cas protein family. We created five different collections of HMM models labeled HMM_1 ... HMM_5 using different hyperparameter values for the clustering algorithm and distinct threshold values for the all-against-all sequence similarity detection (see Methods for detail; the number of models for each Cas protein family is listed in Supplementary Table S1). For a given Cas protein sequence, we applied all HMM models that are contained in a specific collection for that protein family and took the maximum bit score, and zero otherwise. Non-zero values indicate that the investigated protein sequence belongs to the Cas protein family defined by the HMMer model set.

We used different measurements to assess the quality of a specific division represented by a set HMM_i. One quality criteria for a set HMM_i is clearly the capability for detecting known members of Cas proteins. Table 1 shows the sensitivity for the five sets HMM_1 ... HMM_5 by reporting the number of cassettes found in each subtype. It is easy to see that the more fine grained sets, HMM_1, HMM_2 and HMM_3, clearly detect more Cas proteins than the less fine grained sets HMM_4 and HMM_5.

Table 1. Properties and Quality Measurements for the collections HMM_1 ... HMM_5. (a) Sensitivity of set HMM_i in detecting Cas proteins, measured by the number of cassettes found per subtype. Sets HMM_1, HMM_2 and HMM_3 are more fine grained than sets HMM_4 and HMM_5, which detect less Cas proteins overall. (b) Median Accuracy for the classification of subtypes when using set HMM_i with different ML-approaches to determine the evidence for a Cas protein in a cassette. The quality difference is much lower in the overall task of subtype classification compared to the task of detecting individual Cas proteins.

		HMM_1	HMM_2	HMM_3	HMM_4	HMM_5
	No. models	379	385	416	209	201
	No. sequences	14674	14674	23622	16018	16018
(a) Sensitivity per Subtype	I-A	116	116	117	0	0
	I-B	715	715	713	421	421
	I-C	629	629	629	612	612
	I-D	138	138	137	100	100
	I-E	1114	1114	1116	1069	1069
	I-F	354	354	353	339	339
	I-U	136	136	82	8	8
	II-A	320	320	331	249	249
	II-B	28	28	35	35	35
	II-C	327	327	333	328	328
	III-A	376	376	364	326	326
	III-B	292	292	290	178	178
	III-C	93	93	93	83	83
	III-D	184	184	186	49	49
	IV-A	36	36	36	43	43
	V-A	18	18	32	27	27
	VI-A	6	6	4	6	6
	VI-B	40	40	40	40	40
	Total Sensitivy	4922	4922	4891	3915	3915
(b) Accuracy	ERT	0.9900	0.9898	0.9909	0.9907	0.9907
	CART	0.9629	0.9624	0.9636	0.9579	0.9583
	SVM	0.9856	0.9856	0.9830	0.9868	0.9868

In our holistic view of Cas protein detection and subtype classification, however, we want to understand also how the division into subfamilies relates to the cassette subtype, and thus influence the subtype classification. For that reason, we show in Table 1 also as another quality criteria the median accuracy for correctly predicting the subtype of a cassette when using the HMM_i in a ML-based subtype classification approach as described in the next section. The surprising result is that the sensitivity of a specific set HMM_i in detecting Cas proteins does not correlate with the accuracy that is achieved in a subtype classification using this set HMM_i.

A pipeline for CRISPR cassette classification based on Cas protein evidences

Our classification pipeline for CRISPR cassettes is described in Figure 1 and has five steps. For each set HMM_1 ... HMM_5, we build a data matrix for classification and regression analysis of cassettes as follows. Usually, a CRISPR cassette $\mathcal{C}$ is a collection of Cas proteins and is thus defined as a subset of all known Cas proteins $\mathcal{P}$ (i.e., $\mathcal{C} \subset \mathcal{P}$). However, when predicting Cas proteins with HMMer models, this would imply a discretization of the bit score that would omit the information about the *evidence* we have for the prediction. For this reason, we define for each cassette $\mathcal{C}_i$ a real vector X_i of length m, where m is the number of known Cas protein families, containing an en-

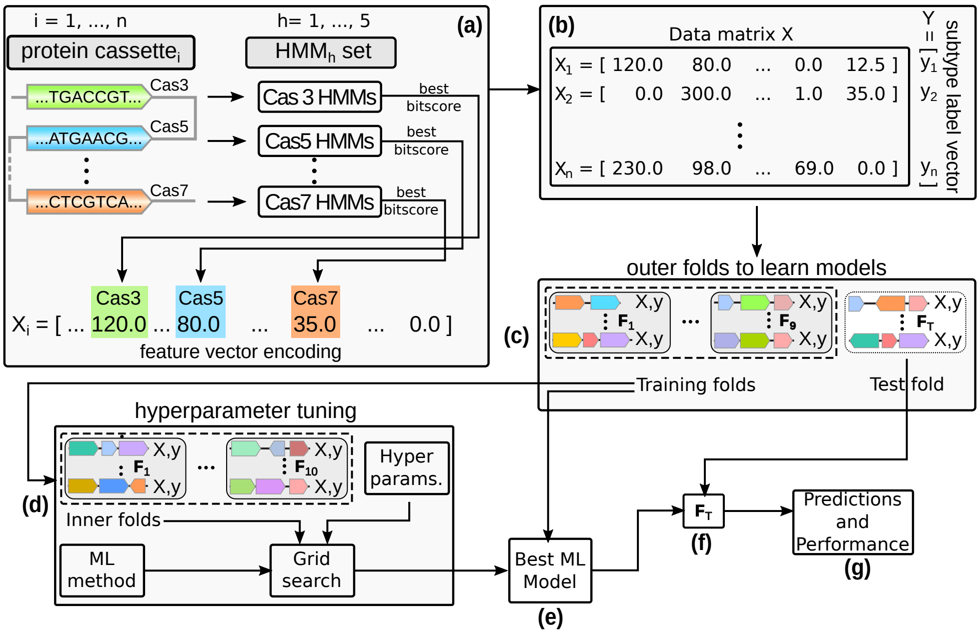

Figure 1. Experimental methodology adopted for this study. (a) Every cassette from our positive set is encoded into a feature vector, which has an entry for each Cas protein family. Given a specific cassette with known Cas proteins, we apply to each Cas protein sequence all HMMs from the set of HMMs that were generated for that specific Cas protein. The best bit-score is included into the feature vector X_i encoding the ith cassette. (b) This feature vector is stored in the data matrix X, together with the known subtype. (c) As the trained model highly depends on the collection of used cassettes, we use the ten-fold cross-validation strategy. Thus, we split the training set into 10 subsets called folds. We perform 10 runs, where, in each run, one of the folds is used for testing and the remaining 9 for selecting and training the best ML model. (d) For selecting the best ML model, a similar cross-validation strategy is applied to tune twenty hyperparameter combinations that affect the model predictive performance. Then, in (e), the selected model is trained using the whole training set. Finally, in (f) and (g) we apply the trained model to the respective test set of the outer fold and evaluate its performance.

try for each possible Cas protein. Each element X_{ij} is defined as the best bit-score obtained by $\mathcal{P}_j$ among all HMM models of its family if it is detectable by the models, and zero otherwise (Figure 1a). By concatenating the vectors obtained for all the n available cassettes, we obtain a data matrix $X \in \mathbb{R}_+^{n \times m}$ (Figure 1b). In addition, each cassette is associated to a label that indicates its subtype, according to the classification provided by [2, 4, 9].

This data matrix, along with the feature vectors and the subtype labels for all known cassettes, is our training data for the subtype classification task. For the evaluation of our classification models, we apply a ten-fold cross-validation procedure on this data matrix. For this, we randomly split the data matrix X into 10 folds (Figure 1c), each one containing a subset of cassettes encoded by the associated feature vector. Each vector is annotated (labeled) by its true subtype. For model selection, we perform hyperparameter tuning by employing a grid search over 20 hyperparameter combinations and applying an inner cross-validation loop (Figure 1d, see Methods for detail). After selecting and training the best model (Figure 1e), we have a classifier that, along with a feature vector with HMM bit scores for all known Cas protein families, predicts the subtype of new cassettes (Figure 1f and Figure 1g).

The classification pipeline successfully predicts the subtype of cassettes

To evaluate the pipeline, we first assessed whether it can successfully perform the classification task, i.e., correctly predict the subtype of a cassette. As shown in Figure 2 for HMM_1, the predictive performances, measured by the adjusted balanced accuracy, for CART, ERT and SVM algorithms are above 95% in general. These high values suggest that, though imbalanced, the cassette subtypes are well-defined in the feature space. It is important to mention that not all cassettes are complete in the investigated datasets. Some cassettes are composed only by subsets of the Cas proteins that integrate its subtype definition. In Supplementary Table S2, we summarize the percentage of cassettes that are complete for each subtype, ignoring Cas proteins that are contained in less than 5% of the cassettes of each subtype. We observed in the experimental results that, even though some incomplete cassettes are present the three classifiers were still able to capture the relations among the remaining proteins. The results for the other four sets of HMM models, and for the F-score with the macro averaging measure were similar and allowed us to draw similar conclusions (see Supplementary Figure S1).

In order to investigate the prediction quality for specific subtypes, we performed an experiment using the *one-vs-the-rest* strategy [34]. Given k different classes, the *one-vs-the-rest* strategy trains k classifiers, one for each subtype, which learns how to discriminate this subtype (positive class) from the remaining classes (negative class). In Table 2 we show the average F-scores, after 50 cross-validation repetitions, obtained by the classifiers using the *one-vs-the-rest* strategy. It is clearly visible that the k classifiers were able to discriminate each class with a high predictive performance, in agreement with our previous results. In the case of SVM, one can use the margin separating positive and negative data as an additional quality criterion [43]. Again one can see here a clear separation of SVM scores for the positive and negative classes (see Supplementary Figure S2).

The classification pipeline detects signature proteins

Makarova et *al.* [2] define the presence of unique signature Cas proteins that characterize most of the investigated CRISPR subtypes. According to the authors, signatures usually consist of either one or multiple Cas proteins that co-occur in the same cassette. Based on the aforementioned results, we hypothesize

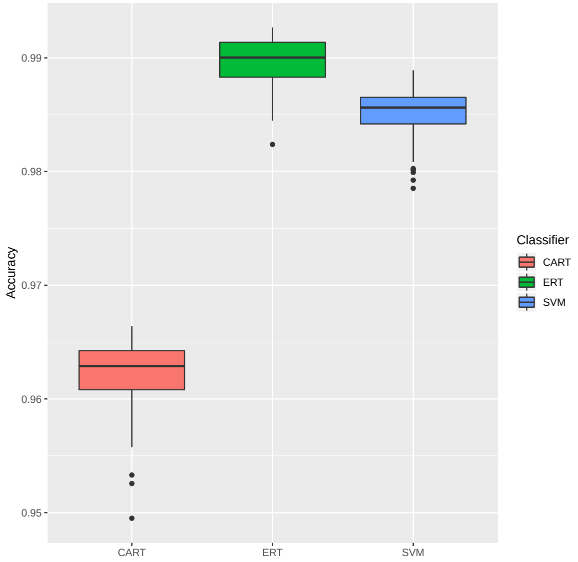

Figure 2. Adjusted balanced accuracy obtained for the 50 repetitions of nested ten-fold cross-validation applying ML algorithms to the dataset generated by the HMM_1 set. The x-axis corresponds to the classifiers trained by different ML algorithms. The y-axis shows the range of adjusted balanced accuracy values.

Table 2. Mean F-scores for 50 nested cross-validation repetitions using the *one-vs-the-rest* strategy and Cas protein set HMM_1.

Subtype	CART	SVM	ERT
I-A	0.95	0.96	**0.98**
I-B	0.95	0.98	**0.99**
I-C	0.98	0.99	**1.00**
I-D	0.98	0.97	**0.99**
I-E	0.99	0.99	**1.00**
I-F	0.95	0.99	**1.00**
I-U	0.99	0.97	**1.00**
II-A	**1.00**	**1.00**	**1.00**
II-B	**1.00**	**1.00**	**1.00**
II-C	**1.00**	**1.00**	**1.00**
III-A	0.98	0.98	**0.99**
III-B	0.97	0.98	**0.99**
III-C	0.93	**0.98**	**0.98**
III-D	0.96	0.97	**0.99**
IV-A	**1.00**	**1.00**	**1.00**
V-A	**1.00**	**1.00**	**1.00**
VI-B	0.86	0.95	**0.96**

that the classifiers were able to learn these signature proteins. Since each *one-vs-the-rest* classifiers introduced in the last section learned how to discriminate a different subtype, we assessed whether it is possible to derive insights about signature proteins for each class by analyzing each classifier separately.

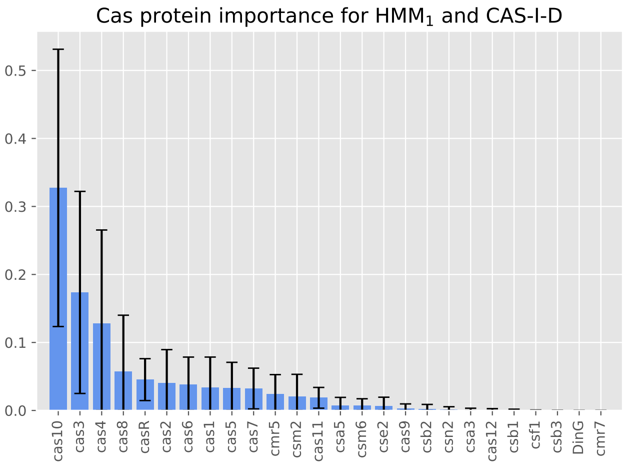

Figure 3. *One-vs-the-rest* average Cas protein importance of ERT for I-D subtype. The x-axis presents different Cas proteins. The y-axis shows the importance of each Cas protein regarding the decision trees of the ensemble split. The error bars refer to the standard deviation over all trees in the ensemble. Note that the feature importance is not only related with the classification into the I-D subtype, but may also be related to its contribution to classify a cassette into any other subtype. Thus, some of the proteins in the figure may not be related to I-D, but to any other subtype.

We thus propose a new approach to detect signature proteins for a subtype by determining the importance of a specific feature (i.e, the evidence for a Cas protein in a cassette) to correctly predict the subtype in the respective *one-vs-the-rest* classifier. The rationale is that Cas proteins which are highly important for discriminating a specific subtype against all others are likely signature proteins for this subtype. Figure 3 shows the importance of each Cas protein (see Methods for definition of feature importance) in predicting the I-D subtype. As it can be seen, the importance is specifically high for Cas10(d) (resp. Cas3), which is the signature protein for the subtype I-D (resp. the type I) according to [2]. Overall, we observed that Cas10 and Cas3 account, on average, for more than 50% of the feature importance for classifying the I-D subtype.

To investigate the relation between the two signature genes for proteins Cas10 and Cas3 in more detail, we selected the decision tree obtained by CART for the I-D subtype (Figure 4). In this tree, terminal nodes with the blue color indicate I-D classification (positive class), while those with brown color indicate any other subtype classification (negative class). As shown in Figure 4, Cas10 is the most important protein for identifying I-D, which is in agreement with [2], where the subtype I-D is characterized by the presence of a variant of the Cas10 protein (instead of a protein from the Cas8 family, which is common for the other I subtypes) and two variants of the Cas3 protein. Interestingly, we need middle to strong evidence for Cas10 and only weak evidence for Cas3. In the case of weak evidence for Cas10, we also need weak evidence for both Cas3 and Cas1 in order to correct the missing 36 examples, albeit in this case the classification would not be pure anymore. Overall, it can be observed that CART was able to correctly model this signature, since most of the nonterminal nodes refer to these proteins, indicating that they are the most important features in this subtype.

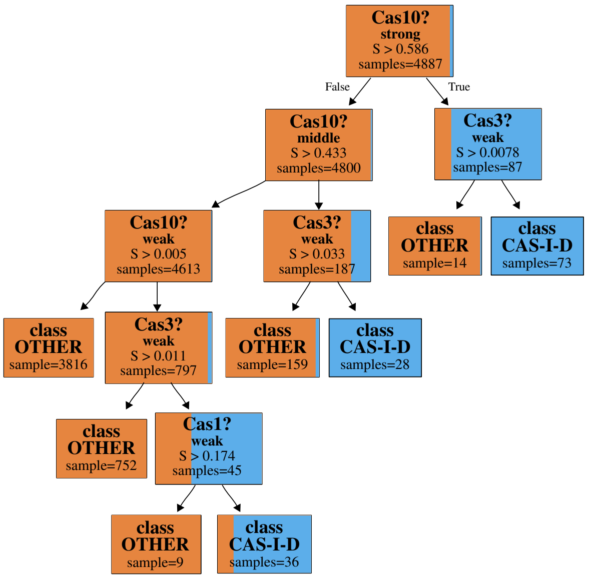

Figure 4. Reduced *one-vs-the-rest* CART for the I-D class (see Supplementary Figure S3 for full tree). Cassetes that are labeled as subtype I-D are highlighted in blue, the others in brown. Each node shows the fractions of class I-D and other cassetes, indicating the purity of the node. The number of cassetes is shown under the "samples" entry. In each node, we query for evidence of a specific Cas protein, indicated by the score calculated by the HMM family models. As one can see, a strong evidence for Cas10 immediately points to a subtype I-D (top node and right branch). Otherwise, if we have middle evidence for Cas10, we need at least weak evidence for Cas3 to determine subtype I-D. Finally, if we have only weak evidence for Cas10, we need at least weak evidence for Cas3 and also for Cas1 to determine subtype I-D (left branch). However, the classification is not pure anymore (bottom nodes).

Since the current classification [2] is based only on the interference module, the adaptation-related Cas proteins (Cas1, Cas2 and Cas4) should not have a high importance for our classification pipeline. Thus, in another experiment, we removed these proteins and the processing proteins (Cas6), and tested the predictive performance of our classification pipeline when removing this information. The obtained results were similar to those previously discussed in this section and support our discussion and main conclusions (see Supplementary Figure S4), strengthening the hypothesis that our ML-based approach captured biologically relevant information.

All the aforementioned examples illustrate how our ML models are able to learn the protein signatures without any extra information other than the normalized bit scores and cassette subtype labels. These results validate our hypothesis and provide models that are able to automatically categorize new cassettes with a high predictive accuracy.

Regression instead of classification learns association rules

In our next set of experiments, we were interested in answering the question of whether some Cas proteins tend to be co-occurring frequently with other proteins. To answer this question, we hypothesized that they form a functional module. However, as we have varying information about the evidence for a specific Cas protein, and there is also some redundancy and flexibility in forming this module, we followed an approach different from that described in the previous section. We believe that if a specific Cas protein is frequently associated with other Cas proteins, it is possible to predict the evidence for this protein by relying only on the known evidence for the other members of the functional module. We can confirm this belief by removing a specific Cas protein from the feature vector, and predicting the "expected" normalized bit score for this protein from the remaining feature vector. This amounts to learning a regression model from known examples.

Association rules can now be inspected by determining again the important features (i.e., Cas proteins) to predict the correct evidence for a specific Cas protein. In Table 3, we list the three most important proteins for some target Cas proteins in some subtypes. In this case, for predicting evidence for Cas10d in subtype I-D, we need the information about Cas3, Cas5 and Cas7. In agreement with the fact that subtypes are mainly associated with the interference complex [2], we find that for the interference proteins Cas10d, Cas3 and Cse2, the associated proteins are also interference proteins. For the non-interference proteins Csn2 and Cas4 in II-A and II-B, not only is Cas9 an interference and signature protein for type II, but it is associated with them as well as the adaptation proteins Cas1 and Cas2. Interestingly, though Cas9 information is important for Cas4, Cas1 is actually more significant for Csn2. This is in agreement with the hypothesized role of Csn2 in the adaptation process [44, 45, 46, 47].

Table 3. Top 3 most important proteins according to ERT when trying to predict a target protein across different subtypes. For the interference proteins Cas10d, Cas3 and Cse2, the other most important Cas proteins are also interference proteins. For non-interference proteins, other Cas proteins linked to adaptation, e.g. Cas1 and Cas2, are also important. The helper protein CasR seems to have different modules associated in I-A and I-E.

Subtype	Target protein	Most important proteins
I-D	Cas10d	(Cas3, 0.28), (Cas5, 0.26), (Cas7, 0.17)
I-D	Cas3	(Cas11, 0.48), (Cas10, 0.20), (Cas5, 0.11)
I-E	Cse2	(Cas7, 0.25), (Cas5, 0.23), (Cas8, 0.19)
II-A	Csn2	(Cas1, 0.62), (Cas9, 0.23), (Cas2, 0.15)
II-B	Cas4	(Cas9, 0.83), (Cas1, 0.09), (Cas2, 0.08)
I-A	CasR	(Csa5, 0.28), (Cas5, 0.16), (Cas6, 0.12)
I-E	CasR	(Cas1, 0.33), (Cas7, 0.25), (Cas8, 0.21)

An interesting case to consider is CasR (also known as CasRA or Csa3), a transcriptional regulator of CRISPR interference and/or adaptation [48, 49]. This protein seems to have different roles in subtypes I-A and I-E, and also appears to be associated with the different proteins in I-A and I-E (see Table 3, last two rows). In I-A, the most important proteins are Csa5, Cas5 and Cas6, whereas in I-E they are Cas1, Cas7 and Cas8.

The ML-approach can handle missing Cas proteins

During our experiments, we left out cassettes that had one or more Cas proteins missing, i.e., without hits in their corresponding HMM models during the preprocessing step (Figure 1a). Since these cases often occur in real application scenarios it is important to assess how our ML-based pipeline can handle them. We observed that most of these cassettes contained only one protein that did not present any hit for the HMM models of its family. For such, we worked with the cassettes having all proteins annotated as ground truth, and removed one bit score for a specific protein. We then learned a model able to predict this bit score using the evidence information from the remaining proteins.

Specifically, we investigated the performance of predicting the missing evidence using the previously described regression approach, trained on all subtypes. The basic idea is that a high predicted evidence for a missing protein is a hint for researchers to perform an in-depth attempt to either annotate the missing protein or to search for new proteins that might replace the function of the missing protein.

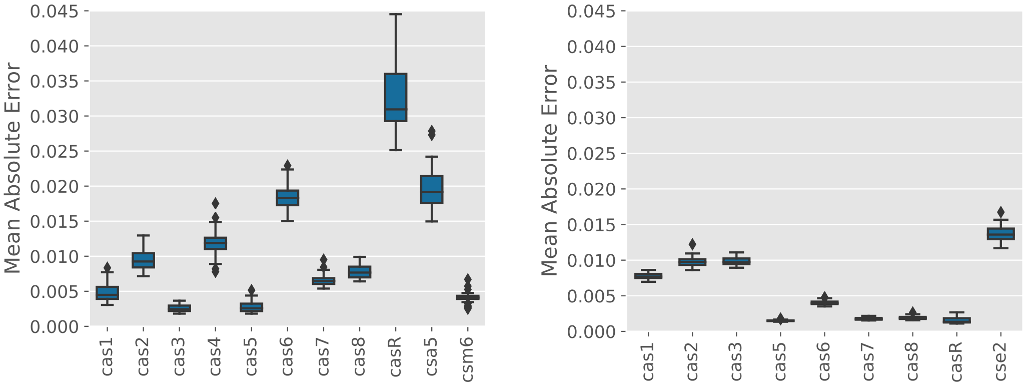

(a) I-A (b) I-E

Figure 5. Mean absolute error rates for Cas proteins contained in I-A (a) and I-E (b) subtypes over 50 nested cross-validation repetitions. The *x*-axis lists the different Cas proteins that were used as target variables. The *y*-axis presents the mean absolute error values between the known bit score, and the bit score predicted by our regression approach. In general, missing proteins are well predicted, especially in the case of the core Cas proteins Cas1 to Cas7. For other Cas proteins, like CasR, the prediction quality varies between I-A and I-E. This is likely due to the higher amount of I-E cassettes in the data basis, indicating a more complex relationship between CasR and other Cas proteins.

Figure 5 shows the Cas protein regression results for ERT; the regressor with the best predictive performance for subtypes I-A and I-E in the dataset generated by HMM_1. Other experimental results, for different subtypes and datasets, can be seen in our Supplementary material, Figures S5–S9. These results show that the missing proteins are predicted with a high quality. For the core proteins Cas1 . . . Cas10, specifically, the proposed approach has very high prediction rates, showing a strong interdependence between these core proteins and other Cas proteins important for the subtype. We also observed that for proteins that are not core Cas proteins such as CasR, the size of the data basis (i.e., number of known cassettes for the subtype where this protein occurs) influences the prediction quality. While this is partially inherent in the machine learning approach, it also might indicate a more variable or complex interaction between these proteins and other proteins important for the subtype.

We also observed that, in general, ERT obtained the best results for Cas protein regression (see Supplementary Figures S5–S9). In most cases, ERT presented mean absolute error values below 0.05 for the normalized bit score prediction. These results confirm the relevance of building specific regressors for each Cas protein inside of a specific subtype for the identification of unknown or possibly missing Cas proteins, when the label of the cassette of interest is known.

In order to assess whether the aforementioned setting would work on a more global level, we replicated the previous experiment by training the regressor on the full datasets with all subtypes. Most of the times, similar results were obtained (see Supplementary Figures S10–S14).

Next, as a proof of concept, we looked at the cassettes with one missing protein that were left out of our experiments and constitute an independent test case (see Methods), and applied our regression approach to identify the cassettes with a high predictive performance of the evidence for having a specific missing protein. These cases would be good candidates for missing annotations. We found 13 cassettes that predicted a missing DinG protein, 3 of them with evidence of at least 0.5. By applying a HHblits [50] search for all ORFs in the respective genome of these three cassettes, we found an ORF with convincing homology to DinG-proteins in each case (see Figure 6A for an example). Another case was Cas2, when we found 13 cassettes with a missing Cas2 protein predicted. We again used HHblits on all ORFs in the genome of the top three cassettes, and found one case with a convincing Cas2 homology (see Figure 6B).

Table 4. Average adjusted balanced accuracy for classification on the independent test set, consisting of cassettes with one Cas protein missing. "Clf." refers to "Classifier" while "Reg." to "Regressor". A dash in the second column means no regression (i.e., only classification) was used.

Clf.	Reg.	HMM_1	HMM_2	HMM_3	HMM_4	HMM_5
CART	–	0.50	0.50	0.68	0.48	0.48
	CART	0.68	0.68	0.52	0.55	0.55
	ERT	0.63	0.63	0.56	0.58	0.58
	SVM	0.56	0.56	0.51	0.54	0.54
ERT	–	0.63	0.63	0.65	**0.74**	**0.74**
	CART	**0.70**	**0.70**	0.63	0.64	0.63
	ERT	0.69	0.69	0.63	0.64	0.65
	SVM	0.60	0.60	0.63	0.63	0.62
SVM	–	0.50	0.50	0.58	0.64	0.64
	CART	**0.72**	**0.72**	0.53	0.58	0.58
	ERT	0.66	0.66	0.60	0.61	0.62
	SVM	0.54	0.54	0.53	0.57	0.57

Finally, we applied our regressors to the aforementioned test set, to predict the missing protein annotations, and the classifiers to predict the subtype for these incomplete cassettes, which were not included in the training set. Table 4 shows the classification results for all ML algorithms on this independent test. In these results, the ERT- and SVM-based classifiers, when combined with the CART regressor and the more fine grained models HMM1 and HMM2, can predict the correct subtype with high predictive performance, even in the hard case of incomplete annotation. The ERT-based classifier can also achieve high performance when combined with the less fine grained models HMM4 and HMM5. However, in these cases, there are less subtypes available, because only classes containing at least 10 examples were included in our experiments (see Methods).

CRISPRcasIdentifier clearly outperforms existing tools

Finally, we assessed the quality of prediction in comparison with existing tools, to assess whether they would be able to correctly classify cassettes that are not covered by the manual annotations. This is a typical application scenario e.g. in the analysis of cassettes from metagenomic data. For this purpose, we used CRISPRcasIdentifier with default parameters and

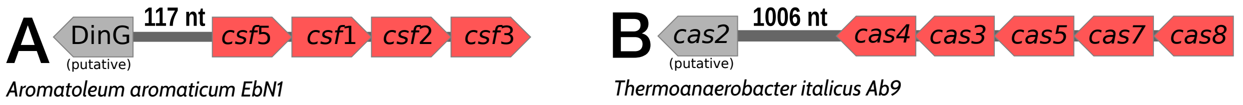

Figure 6. The cassettes with missing proteins. A) In this genome, we predicted a DinG protein missing in the cassette with evidence > 0.5. The HHblits [50] search in this genome for all ORFs determined 1 ORF 117nt upstream of the cassette with a high confidence score for a DinG homology (E-value: 7.6e-22). B) In the case of Cas2, the predicted evidence was lower, between 0.221 to 0.165. Nevertheless, we found 1 ORF with a high confidence score for Cas2 homology (E-value: 1e-37) 3010nt downstream of the cassette.

Table 5. Predictive performance of CRISPR-Cas tools for different measures. The best results for each measure are marked in bold.

	Tool	Adj. Bal. Acc.	F-score
web-server	CRISPRone	0.07	0.17
	HmmCas	0.05	0.15
command-line	CRISPRdisco	0.52	0.63
	CRISPRcasFinder	0.48	0.56
	Macsyfinder	0.54	0.60
	CRISPRcasIdentifier	**0.89**	**0.91**

compared its performance with three command line CRISPR-Cas tools (CRISPRdisco [18], CRISPRcasFinder [19] and Macsyfinder [20]) and two webservers (CRISPRone [21] and HmmCas [22]).

To benchmark these tools we used the most recent and comprehensive set of cassettes as listed in the very recent classification paper [51]. This dataset has 6098 cassettes extracted from 4974 archaeal and bacterial genomes, including the following subtypes: I-A to I-U, II-A to II-C, III-A to III-D, IV-A, V-A and VI-B. In Table 5 we present the adjusted balanced accuracy scores and F-scores with the macro averaging obtained. The inferior results of HmmCas and CRISPRone can partially be explained by the fact that they 1) use the existing Cas HMMer models without any enhancement, and 2) rely on a concept that is similar to signature gene for predicting the subtype.

According to Table 5 our tool clearly outperforms the others for all measures. Our hypothesis is that the superior results are due to the generalization capability of ML models. Thus, our tool is more suitable to handle unseen examples even if they contain missing proteins. It occurs because it does not rely only on HMM profile searches but also on the general knowledge extracted from the training data. It is also important to observe that CRISPRcasIdentifier not only classifies unseen cassettes but also tries to predict potentially missing proteins which, to the best of our knowledge, is a problem that has not been successfully addressed by the existing tools.

Conclusion

In this paper we introduced a new ML-based pipeline for the identification and classification of genomic CRISPR-Cas systems. To assess the predictive performance of this approach, we conducted an in-depth investigation into the suitability of ML algorithms that are commonly used for this task, by using the normalized profile HMM search bit scores of Cas proteins as input and classifying cassettes encoding Cas proteins to their respective subtypes according to the most recent classification [2, 4, 9].

Overall, this work covers four different research issues: (i) the classification of Cas cassettes; (ii) the prediction of normalized bit scores for missing Cas proteins; (iii) the investigation of the properties of CRISPR types and subtypes; and (iv) the comparison of our new tool to the ones available in the literature. Concerning topic (i), our classification models were able to achieve very high classification performance, above 0.95, in terms of the adjusted balanced accuracy score. Thus, they are well placed for the prediction of CRISPR systems of newly sequenced organisms, or metagenomic data with sufficient read length to cover the full cassette in one contig. In addition, we introduced a new method for determining signature genes, which are genes most important for predicting the correct subtype. This approach was able to properly learn the known signature genes of CRISPR-Cas subtypes without any extra information other than the available gene cassettes and their labels, but provides additional information about the composition of cassettes. In topic (ii), our regressor models achieved very small deviations between the expected and predicted normalized bit scores for different Cas proteins across the different subtypes. This illustrates the usefulness of these regressors on new cassettes that have missing hits for some Cas proteins. A high bit score provides a hint to researchers to search for more diverged forms of the protein, or to look for proteins which could replace the missing function. The analysis performed under topic (iii) enabled us to correctly identify known signature genes, and to identify putative functional modules. Overall, it provided us with a set of association rules for potential use in more advanced classification scenarios, in addition to providing insights about the biology of the systems. Finally, concerning (iv), our tool outperformed five other tools from the literature on the most recent and comprehensive CRISPR classification dataset published.

Manual annotation is the gold standard when it comes to classification and identification of genomic CRISPR-Cas systems. Supporting this process or annotating cassettes as part of an overall automatic pipeline such as the analysis of metagenomic data requires a classification approach with a degree of flexibility that is challenging to model. CRISPRcasIdentifier provides a boost in classification accuracy when compared to existing tools, because it builds not only on an understanding of the manual annotation process but also on the generalization power of ML algorithms. We made CRISPRcasIdentifier available for researchers to use with their own data.

Availability of Source Code and Requirements

Project name: CRISPRcasIdentifier
Project home page: https://github.com/BackofenLab/CRISPRcasIdentifier
RRID: SCR_018296
BiotoolsID: crisprcasidentifier
Operating system(s): Platform independent
Programming language: Python
Other requirements: Anaconda, Docker
License: GNU General Public License version 3 (GPLv3)

Availability of Supporting Data and Materials

The data that supports the present work are available from the studies of [2, 3, 4, 51] and also in our supplementary material.

Declarations

List of abbreviations

CART: Classification and Regression Trees; crRNA: CRISPR interference RNAs; ERT: Extremely Randomized Trees; HMM: Hidden Markov Models; MCL: Markov Cluster Algorithm; ML: machine learning; PAM: protospacer adjacent motif; SVM: Support Vector Machines.

Competing interests

The authors declare that they have no competing interests.

Funding

This research was supported by the Federal Agency for Support and Evaluation of Graduate Education within the Ministry of Education of Brazil (CAPES) (Probral CAPES/DAAD grant #88887.302257/2018-00), the São Paulo Research Foundation (FAPESP) (grants #2013/07375-0, #2016/18615-0 and #2019/21300-9), Intel, and the German Research Foundation (DFG) (grants BA 2168/13-1 SPP 1590 Probabilistic Structures in Evolution and BA 2168/23-1 SPP 2141 Much more than Defence: the Multiple Functions and Facets of CRISPR-Cas).

Authors' contributions

V.A.P., O.S.A., A.C.P.L.F.C. and R.B. designed the study, analysed the data and conceived the methods. V.A.P. implemented and performed the experiments. O.S.A. and S.A.S. acquired the data. V.A.P., O.S.A., S.A.S., A.C.P.L.F.C. and R.B. planned and wrote the manuscript. All authors read and approved the final manuscript.

References

1. Garneau JEo. The CRISPR/Cas bacterial immune system cleaves bacteriophage and plasmid DNA. Nature 2010;468(7320):67–71.
2. Makarova KS, Wolf YI, Alkhnbashi OS, Costa F, Shah SA, Saunders SJ, et al. An Updated Evolutionary Classification of CRISPR-Cas Systems. Nature Reviews Microbiology 2015 Nov;13(11):722–736.
3. Shmakov S, Abudayyeh OO, Makarova KS, Wolf YI, Gootenberg JS, Semenova E, et al. Discovery and functional characterization of diverse class 2 CRISPR-Cas systems. Molecular cell 2015;60(3):385–397.
4. Shmakov S, et al. Diversity and evolution of class 2 CRISPR–Cas systems. Nature Reviews Microbiology 2017;.
5. Cass SDB, Haas KA, Stoll B, Alkhnbashi O, Sharma K, Urlaub H, et al. The role of Cas8 in type I CRISPR interference. Biosci Rep 2015;35(4):e00197.
6. Sinkunas T, Gasiunas G, Fremaux C, Barrangou R, Horvath P, Siksnys V. Cas3 is a single-stranded DNA nuclease and ATP-dependent helicase in the CRISPR/Cas immune system. EMBO J 2011;30(7):1335–42.
7. Zhang J, et al. Structure and mechanism of the CMR complex for CRISPR-mediated antiviral immunity. Mol Cell 2012;45(3):303–13.
8. Deng L, et al. Modulation of CRISPR locus transcription by the repeat-binding protein Cbp1 in Sulfolobus. NAR 2012;40(6):2470–80.
9. Shah SA, et al. Comprehensive search for accessory proteins encoded with archaeal and bacterial type III CRISPR-cas gene cassettes reveals 39 new cas gene families. RNA Biology 2018;0(0):1–13. PMID: 29911924.
10. Haft DH, et al. A guild of 45 CRISPR-associated (Cas) protein families and multiple CRISPR/Cas subtypes exist in prokaryotic genomes. PLoS Comput Biol 2005;1(6):e60.
11. Makarova KS, et al. Evolution and classification of the CRISPR-Cas systems. Nat Rev Microbiol 2011;9(6):467–77.
12. Marchler-Bauer A, Bryant SH. CD-Search: protein domain annotations on the fly. Nucleic Acids Research 2004 07;32(suppl_2):W327–W331. https://doi.org/10.1093/nar/gkh454.
13. Lange SJ, Alkhnbashi OS, Rose D, Will S, Backofen R. CRISPRmap: An Automated Classification of Repeat Conservation in Prokaryotic Adaptive Immune Systems. Nucleic Acids Research 2013 Jan;41(17):8034–8044.
14. Alkhnbashi OS, Costa F, Shah SA, Garrett RA, Saunders SJ, Backofen R. CRISPRstrand: Predicting Repeat Orientations to Determine the crRNA-Encoding Strand at CRISPR Loci. Bioinformatics (Oxford, England) 2014 Sep;30(17):i489–496.
15. Biswas A, Staals RHJ, Morales SE, Fineran PC, Brown CM. CRISPRDetect: A flexible algorithm to define CRISPR arrays. BMC Genomics 2016;p. i356–i356.
16. Alkhnbashi OS, et al. Characterizing leader sequences of CRISPR loci. Bioinformatics 2016;32(17):i576–i585.
17. Finn RD, Clements J, Eddy SR. HMMER web server: interactive sequence similarity searching. Nucleic acids research 2011;39(suppl_2):W29–W37.
18. Crawley AB, et al. CRISPRdisco: An Automated Pipeline for the Discovery and Analysis of CRISPR-Cas Systems. The CRISPR Journal 2018 Apr;1(2):171–181.
19. Couvin D, Bernheim A, Toffano-Nioche C, Touchon M, Michalik J, Néron B, et al. CRISPRCasFinder, an Update of CRISRFinder, Includes a Portable Version, Enhanced Performance and Integrates Search for Cas Proteins. Nucleic Acids Research 2018 Jul;46(W1):W246–W251.
20. Abby SS, et al. MacSyFinder: A Program to Mine Genomes for Molecular Systems with an Application to CRISPR-Cas Systems. PLOS ONE 2014 10;9(10):1–9. https://doi.org/10.1371/journal.pone.0110726.
21. Zhang Q, Ye Y. Not all predicted CRISPR–Cas systems are equal: isolated cas genes and classes of CRISPR like elements. BMC Bioinformatics 2017 2;.
22. Chai G, Yu M, Jiang L, Duan Y, Huang J. HMMCAS: a web tool for the identification and domain annotations of Cas proteins. IEEE/ACM Transactions on Computational Biology and Bioinformatics (TCBB) 2019;16(4):1313–1315.
23. Pearson WR, Lipman DJ. Improved tools for biological sequence comparison. Proceedings of the National Academy of Sciences 1988;85(8):2444–2448. https://www.pnas.org/content/85/8/2444.
24. Enright AJ, et al. An Efficient Algorithm for Large-Scale Detection of Protein Families. Nucleic Acids Research 2002 Apr;30(7):1575–1584.
25. Edgar RC. MUSCLE: multiple sequence alignment with high accuracy and high throughput. Nucleic Acids Research 2004 Mar;32:1792–1797.
26. Hyatt D, Chen GL, LoCascio PF, Land ML, Larimer FW, Hauser LJ. Prodigal: prokaryotic gene recognition and translation initiation site identification. BMC Bioinformatics 2010 03;.
27. Breiman L, et al. Classification and regression trees. Chapman & Hall/CRC; 1984.
28. Vapnik V. The nature of statistical learning theory. Springer-Verlag; 1995.
29. Geurts P, et al. Extremely randomized trees. Machine learning 2006;63(1):3–42.
30. Wu X, Kumar V, Quinlan JR, Ghosh J, Yang Q, Motoda H,

et al. Top 10 algorithms in data mining. Knowledge and information systems 2008;14(1):1–37.
31. Varma S, Simon R. Bias in error estimation when using cross-validation for model selection. BMC bioinformatics 2006;7(1):91.
32. Cawley GC, Talbot NL. On over-fitting in model selection and subsequent selection bias in performance evaluation. Journal of Machine Learning Research 2010;11(Jul):2079–2107.
33. Krstajic D, et al. Cross-validation pitfalls when selecting and assessing regression and classification models. Journal of cheminformatics 2014;6(1):10.
34. Bishop CM. Pattern recognition and machine learning. springer; 2006.
35. Hastie T, Tibshirani R, Friedman J. The Elements of Statistical Learning: Data Mining, Inference and Prediction. 2 ed. Springer; 2009.
36. Forman G, Scholz M. Apples-to-apples in cross-validation studies: pitfalls in classifier performance measurement. ACM SIGKDD Explorations Newsletter 2010;12(1):49–57.
37. Brodersen KH, Ong CS, Stephan KE, Buhmann JM. The balanced accuracy and its posterior distribution. In: 2010 20th International Conference on Pattern Recognition IEEE; 2010. p. 3121–3124.
38. Guyon I, Bennett K, Cawley G, Escalante HJ, Escalera S, Ho TK, et al. Design of the 2015 chalearn automl challenge. In: 2015 International Joint Conference on Neural Networks (IJCNN) IEEE; 2015. p. 1–8.
39. Sokolova M, Lapalme G. A systematic analysis of performance measures for classification tasks. Information Processing & Management 2009;45(4):427–437.
40. Willmott CJ, Matsuura K. Advantages of the mean absolute error (MAE) over the root mean square error (RMSE) in assessing average model performance. Climate research 2005;30(1):79–82.
41. Pedregosa F, Varoquaux G, Gramfort A, Michel V, Thirion B, Grisel O, et al. Scikit-learn: Machine learning in Python. Journal of machine learning research 2011;12(Oct):2825–2830.
42. Hsu CW, Chang CC, Lin CJ, et al. A practical guide to support vector classification 2003;.
43. Cherkassky V, Dhar S. Simple Method for Interpretation of High-Dimensional Nonlinear SVM Classification Models. In: 6th International Conference on Data Mining; 2010. p. 267–272.
44. Nam KH, et al. Crystal structure of clustered regularly interspaced short palindromic repeats (CRISPR)-associated Csn2 protein revealed Ca2+-dependent double-stranded DNA binding activity. J Biol Chem 2011 09;35:30759–68.
45. Koo Y, et al. Crystal Structure of Streptococcus pyogenes Csn2 Reveals Calcium-Dependent Conformational Changes in Its Tertiary and Quaternary Structure. PLOS ONE 2012 03;7(3):1–8.
46. Arslan Z, et al. Double-strand DNA end-binding and sliding of the toroidal CRISPR-associated protein Csn2. Nucleic Acids Research 2013 04;41(12):6347–6359.
47. Lee KH, et al. Identification, structural, and biochemical characterization of a group of large Csn2 proteins involved in CRISPR-mediated bacterial immunity. Proteins 2012;80 11:2573–82.
48. He F, Vestergaard G, Peng W, She Q, Peng X. CRISPR-Cas type I-A Cascade complex couples viral infection surveillance to host transcriptional regulation in the dependence of Csa3b. Nucleic Acids Research 2017 2;.
49. Vestergaard G, Garrett RA, Shah SA. CRISPR adaptive immune systems of Archaea. RNA biology 2014 11;.
50. Remmert M, , et al. HHblits: lightning-fast iterative protein sequence searching by HMM-HMM alignment. Nature Methods 2012 02;9:173–175.
51. Makarova KS, Wolf YI, Iranzo J, Shmakov S, Alkhnbashi OS, Costa F, et al. Evolutionary classification of CRISPR–Cas systems: a burst of class 2 and derived variants. Nature Reviews Microbiology 2019;p. 1–17.

Click here to access/download
Supplementary Material
CRISPRcasIdentifier_Supplementary_Material.pdf

Personal Cover

Click here to access/download;Personal Cover;Cover-letter_and_Response.pdf

GigaScience

Dear Dr. Hans Zauner

Herewith we submit the revised version of our manuscript

CRISPRcasIdentifier: Machine learning for accurate identification and classification of CRISPR-Cas systems

We have answered each issue and have modified our submission where necessary. We have taken careful consideration of the reviewers' comments and have integrated them into a thoroughly revised version of the manuscript. Additional to the reviewers comments, we took your suggestion into consideration. We are grateful for the constructive comments that have significantly improved the presentation of our work.

Please find a point-by-point response to the reviews below.

I am looking forward to hearing from you.

Sincerely yours,
Prof. Dr. Rolf Backofen

Point-to-point response to reviewer comments:

Editor suggestion

1- I particularly agree with reviewer 1 that clarifying all data sources and improving the "readme" are two important points to address. Please also consider the suggestion of reviewer 2 to provide a dockerized version for end users' convenience. In addition, please register any new software application in the bio.tools and SciCrunch.org databases to receive RRID (Research Resource Identification Initiative ID) and biotoolsID identifiers, and include these in your manuscript. This will facilitate tracking, reproducibility and re-use of your tool

Authors' response: First, we better clarified the data sources by providing one extra table in our GitHub repository (https://github.com/BackofenLab/CRISPRcasIdentifier, see Supplementary_files folder under the GitHub) which lists all CRISPR cassettes used and from which source each one of them came from. In addition, our repository includes a detailed README.md file (which describes the installation process and usage) and we also created a dockerized version (https://hub.docker.com/repository/docker/padilha/crispr-cas-identifier), as suggested by reviewer #2. Finally, we also registered our tool in SciCrunch.org (RRID: SCR_018296) and bio.tools (biotoolsID: crisprcasidentifier) and included the respective IDs in the "Availability of Source Code and Requirements" section.

Reviewer #1: *The computational tool that the authors present for identifying CRISPR-Cas systems can potentially save researchers time and effort as well as assisting in identifying novel Cas proteins. Although not the first tool in this space, CRISPRCasIdentifier does appear to outperform pre-existing tools by a large margin. Modelling is based on a good-sized dataset and the ML component appropriately features cross-validation and tuning. The paper is well-written with minimal grammatical errors.*

Manuscript comments:
1- I understand that the dataset appears to be from multiple sources with a total of 68594 samples. Is this the data from [2,3,4]? A table (perhaps in supplementary) outlining how much and from where all of the data comes from would help clarify this.

Authors' response: Done. Please see the supplementary file named CRISPR_cassette_source.tab in our GitHub repository (https://github.com/BackofenLab/CRISPRcasIdentifier).

2 - Although both terms are appropriate, "trained" seems to be more common vocabulary than

"induced" based on a quick literature search of recent manuscripts. I don't believe all instances of induced in the manuscript must be replaced with trained, but I do believe trained sounds more natural so should perhaps be considered. I.e. in the abstract "models induced by using machine learning" compared with "models trained using machine learning".

Authors' response: Thank you for the suggestion**,** we changed all occurrences of the word "induce" to "train" in the text.

3 - Please check the grammar of the following phrase in "Data collecting and preprocessing" section: "is a pair of the genomic sequence containing all genes of the cassette".

Authors' response: We checked the grammar and corrected the sentence.

4 - Should the title of that section be "Data collection and preprocessing"?

Authors' response: We agree with the reviewer and changed the title of the section.

5 - Is hyphenation intended in this sentence? "and as a result re-assure key components of subtypes like"

Authors' response: The correct term is reassure. We corrected it in the text.

6- I notice the project web page is password-protected, although the password is provided in the manuscript. Is password-protection a temporary measure until the paper is accepted?
I believe it would be more user-friendly to have the project available as a GitHub repository with README.md available upfront, rather then requiring the user to follow one set of instructions before being able to view it.
Also, as I'm sure you know, this has the benefit of tagged releases for better reproducibility, given the inevitable bugfixes/improvements of bioinformatics tools.

Authors' response: Yes, the password protection was a temporary measure for the review process. We moved the code to a public GitHub repository. It is now available with a proper README.md file in: https://github.com/BackofenLab/CRISPRcasIdentifier. We also created a tagged release for the version we used in our paper.

7- Program comments:

- *Biopython should be listed as a requirement in README.md*
- *The examples in README.md on lines 78 and 79 contain "fasta" extensions but the respective files in the "examples" directory have a "fa" extension.*

** The program looks for "models" directory or "models.tar.gz" archive. If I follow the instructions I end up with a "trained_models_2015" directory. Therefore the program fails unless this directory is renamed.*

Authors' response: We thank the reviewer for all the above comments. We corrected them as suggested.

Reviewer #2:

The manuscript is well written and the conclusion is backed up by a comprehensive banchmark and data data collection. The tools are available and the code is documented.

My only concern is that my Mac had a hard time resolving the conda environment. More testing of the boiler plate code to allow others to deploy your methods is warranted prior to publication. (a docker container based solution through docker hub might be quick to implement and make things easier for end-users)

Despite this, I think the manuscript describes an elegant solution to a timely problem that may likely help many researches to find and understand CRISPR systems in more organisms.

Authors' response: We would like to thank the reviewer for this comment. We now provide the users with three options to install our tool:

- They may use a DockerHub image, available in https://hub.docker.com/repository/docker/padilha/crispr-cas-identifier;
- They may build our docker image from the Dockerfile available in our GitHub repository (https://github.com/BackofenLab/CRISPRcasIdentifier); or
- They may set a conda environment up from our crispr-env.yml file, also available in our Github repository.

In the README.md file we explain each of the aforementioned options in detail.
