# Supplementary material for: CRISPRcasIdentifier: Machine learning for accurate identification and classification of CRISPR-Cas systems
Source: Gigascience. 2020 Jun 17;9(6):giaa062. doi: 10.1093/gigascience/giaa062 (PMC7298778; doi:10.1093/gigascience/giaa062)
Supplement: giaa062_Supplemental_File [file giaa062_supplemental_file.pdf]

CRISPRCasIdentifier: Machine learning for accurate identification and classification of CRISPR-Cas systems – Supplementary Material

Victor A Padilha, Omer S Alkhnbashi, Shiraz A Shah, André C P L F de Carvalho and Rolf Backofen

May 13, 2020

In Table S1, we summarize the five different collections of HMM models, labeled $HMM_1 \ldots HMM_5$, by listing the number of models for each Cas protein family.

Table S1: Number of models for each Cas protein family

Cas Protein	# of models HMM1	# of models HMM2	# of models HMM3	# of models HMM4	# of models HMM5
cas1	17	18	15	10	8
cas10	18	18	18	6	6
cas11	2	2	2	2	2
cas12			2	4	4
cas13			3	4	4
cas2	90	93	78	35	38
cas3	25	25	19	14	10
cas4	18	19	19	12	12
cas5	37	35	34	17	17
cas6	36	36	37	4	4
cas7	19	21	19	11	15
cas8	38	38	48	27	20
cas9	4	3	11	6	4
casR	2	1	2	2	2
cmr1	5	5	3	2	2
cmr3	2	2	2	3	3
cmr4	2	2	2	2	2
cmr5	3	3	3	4	4
cmr6	3	3	2	3	3
cmr7	1	1		1	1
cmr8				1	1
cpf1	1	1			
csa3	1	1	1		
csa5	2	2	2	1	1
csaX				1	1
csb1	1	1		1	1
csb2	1	1	3	1	1
csb3	1	1		1	1
csc1	1	1	2	1	1
csc2	1	1	1	1	1
cse1				1	1
cse2	2	2	17	1	1
csf1	1	1	1	2	2
csf2	1	1	1	2	2
csf3	1	1	1	2	2
csf4	1	1	1	2	2
csf5			1	1	1
csm2	4	4	4	3	3
csm3	22	25	5	5	5
csm4	3	3	5	2	2
csm5	2	2	2	2	2
csm6	6	6	7	1	1
csn2	5	5	4	1	1
csx			39		
csx10				2	2
csx17				1	1
csx19				1	1
csy1				1	1
csy2				1	1
csy3				1	1
	379	385	416	209	201

In Supplementary Table S2, we summarize the percentage of cassettes that are complete for each subtype, ignoring Cas proteins that are contained in less than 5% of the cassettes of each subtype. We observed in the experimental results that, even though some incomplete cassettes are present, the three classifiers were still able to capture the relations among the remaining proteins. The complete results for all sets of HMM models and the two evaluation measures (adjusted balanced accuracy and F-score) are presented in the Supplementary Figure S1.

Table S2: Percentage of complete cassettes across the different subtypes after ignoring Cas proteins that are present in less than 5% of the cassettes of each subtype.

Subtype	% Complete cassettes
I-A	26.72
I-B	71.89
I-C	77.42
I-D	8.7
I-E	80.34
I-F	84.46
I-U	0.0
II-A	93.44
II-B	60.71
II-C	83.79
III-A	29.79
III-B	3.08
III-C	11.83
III-D	1.63
IV-A	0.0
V-A	55.56
VI-B	100.0

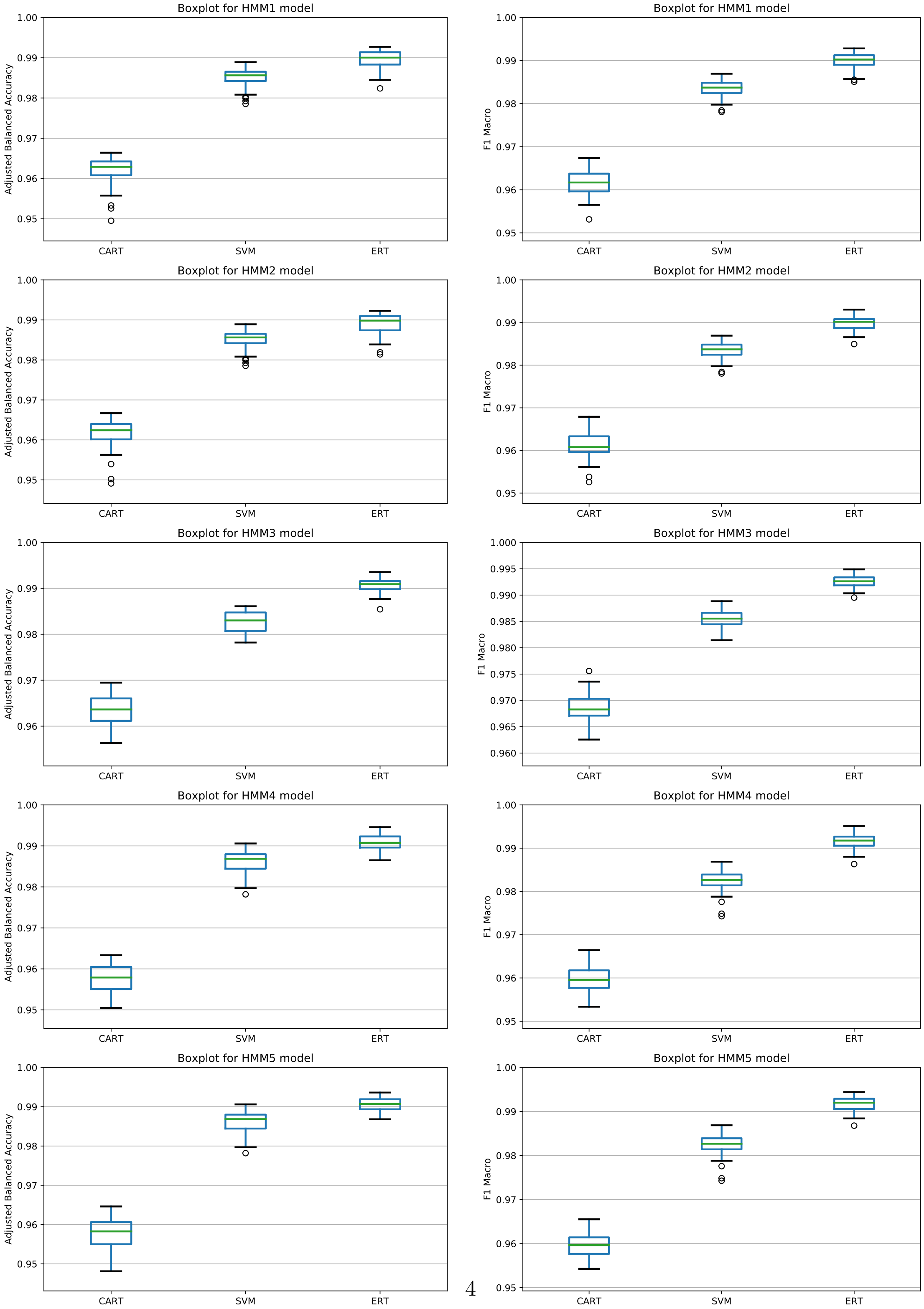

(a) Adjusted balanced accuracy.

(b) F-score.

Figure S1: Adjusted balanced accuracy and F-score values achieved for 50 nested ten-fold cross-validation repetitions in all datasets.

Concerning our *one-vs-the-rest* experiments we can use, in the case of SVM, the margin separating positive and negative data as an additional quality criteria. In Figure S2, we present an example with subtype I-D, where a clear separation of SVM scores for the positive (I-D) and negative classes (other subtypes) can be observed.

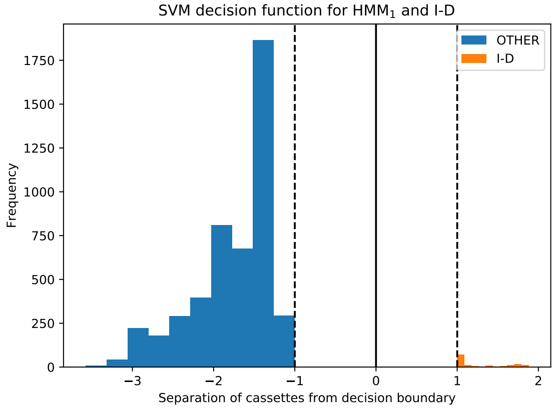

Figure S2: *One-vs-the-rest* SVM histogram of projections (Cherkassky and Dhar, 2010) for the I-D subtype. The solid line represents SVM's optimal hyperplane. The dashed lines represent SVM's margins. The x-axis corresponds to the distance of a cassette to the decision boundary. The y-axis indicates the frequency of cassettes that have different distances to the decision boundary.

In Figure S3, we present the full *one-vs-the-rest* CART for the I-D subtype. As one can see, a strong evidence for Cas10 immediately points to a subtype I-D (top node and right branch). Otherwise, if we have middle evidence for Cas10, we need at least weak evidence for Cas3 to determine subtype I-D. Finally, if we have only weak evidence for Cas10, we need at least weak evidence for Cas3 and also for Cas1 to determine subtype I-D (left branch). However, the classification is not pure anymore.

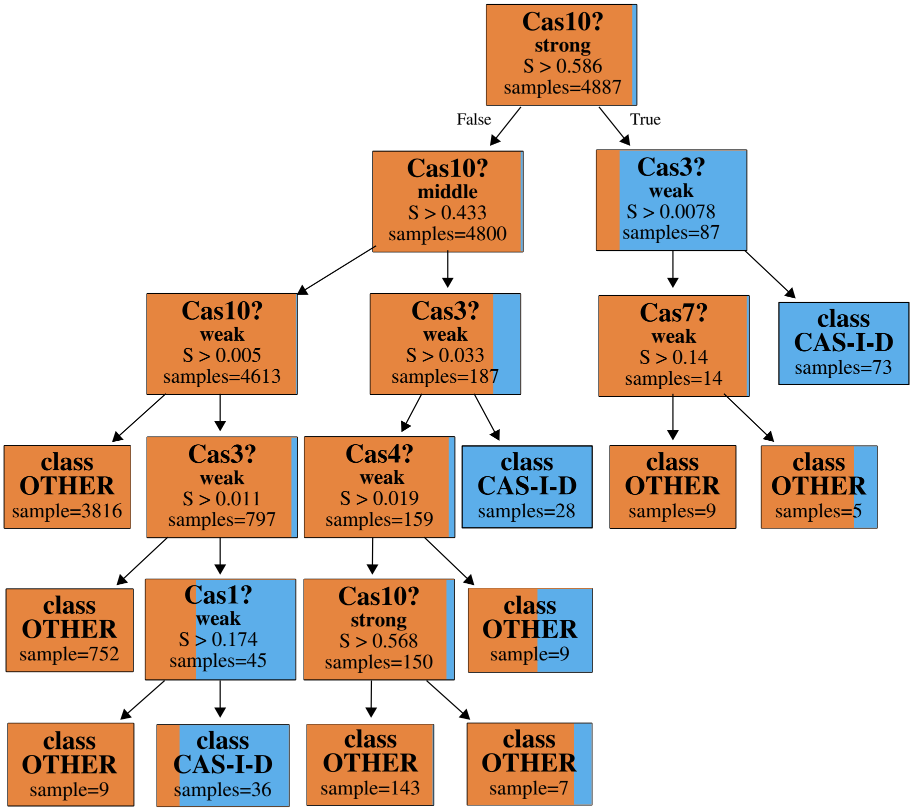

Figure S3: Full *one-vs-the-rest* CART for the I-D subtype. Cassettes that are labeled as subtype I-D are labeled in blue, the others in brown. Each node shows the fractions of class I-D and other cassettes, indicating the purity of the node. The number of cassettes is shown under "samples" entry.

Since the current classification (Makarova *et al.*, 2015) is based only on the interference module, the adaptation-related Cas proteins (Cas1, Cas2 and Cas4) should not have a high importance for our classification pipeline. Thus, we removed, in another experiment, these proteins and the process proteins (Cas6), and tested the predictive performance of our classification pipeline when removing this information. The obtained results were similar to those discussed in our paper and support our discussion and main conclusions (see Supplementary Figure S4), strengthening the hypothesis that our ML-based approach captured biologically relevant information.

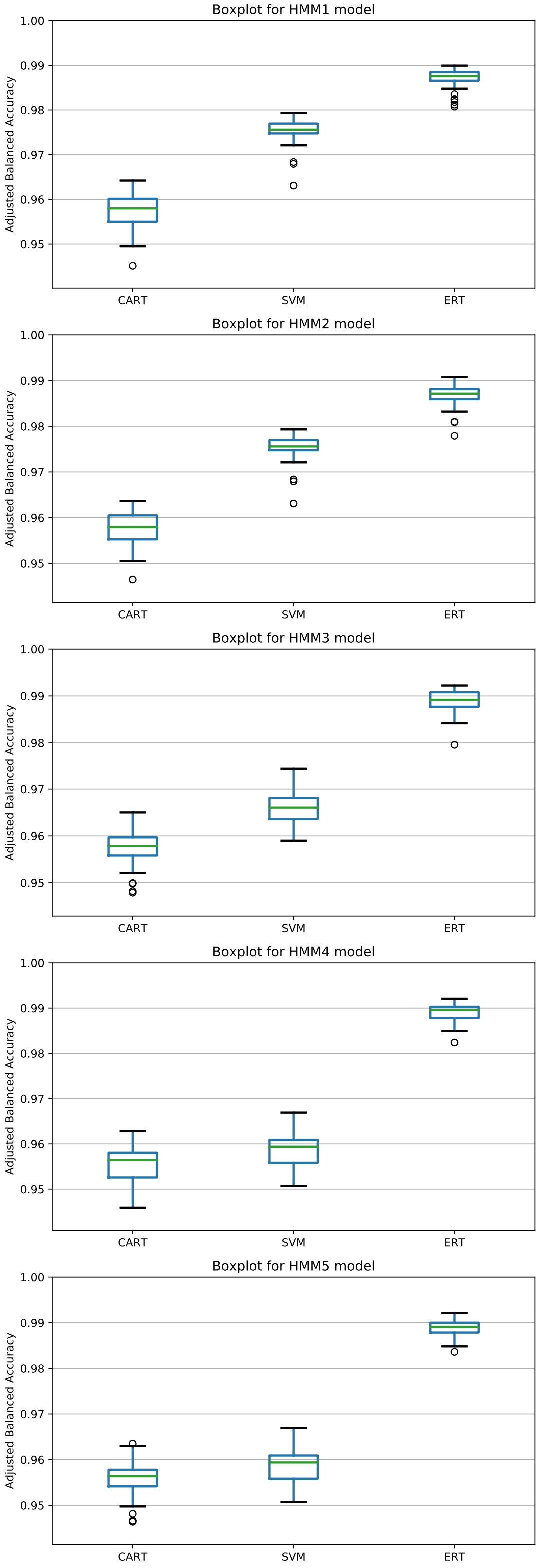

(a) Adjusted balanced accuracy.

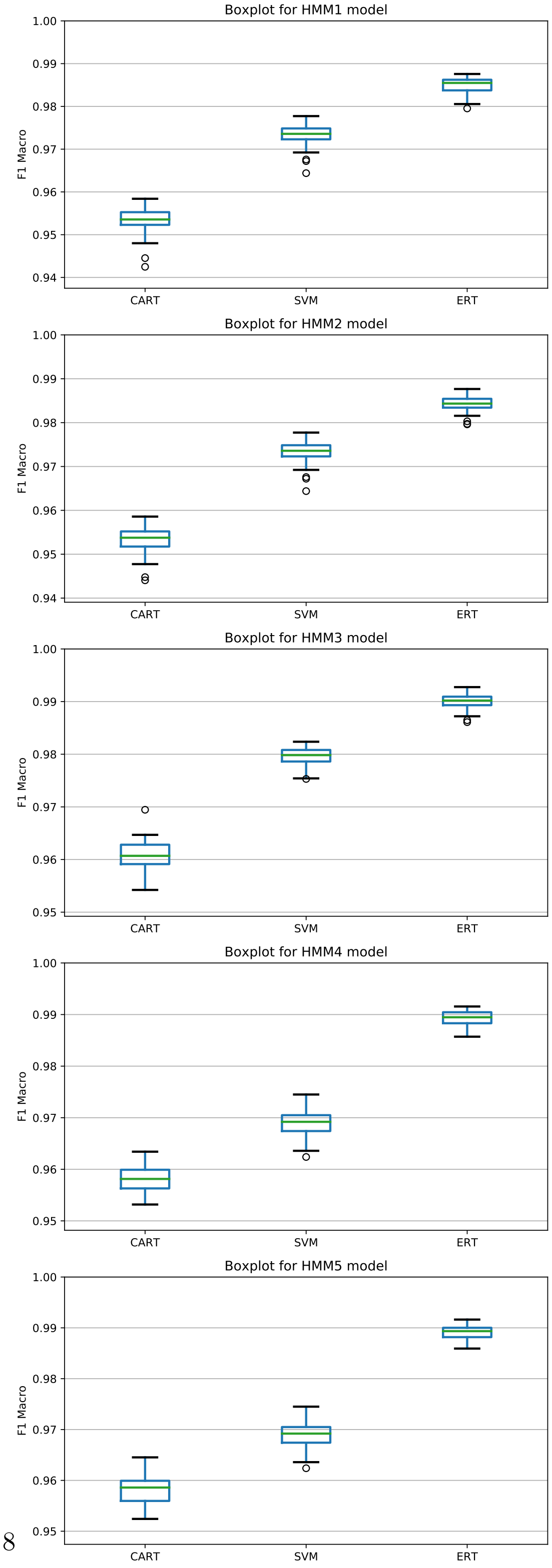

(b) F-score.

Figure S4: Adjusted balanced accuracy and F-score values achieved for 50 nested ten-fold cross-validation repetitions in all datasets after removing Cas1, Cas2, Cas4 and Cas6.

In Figures S5–S9 we present the remaining regression results, concerning the other subtypes and datasets. In general, we can observe that the proteins can be well predicted. In Figures S10–S14, we present regression results considering the full datasets (i.e., not separating by subtype).

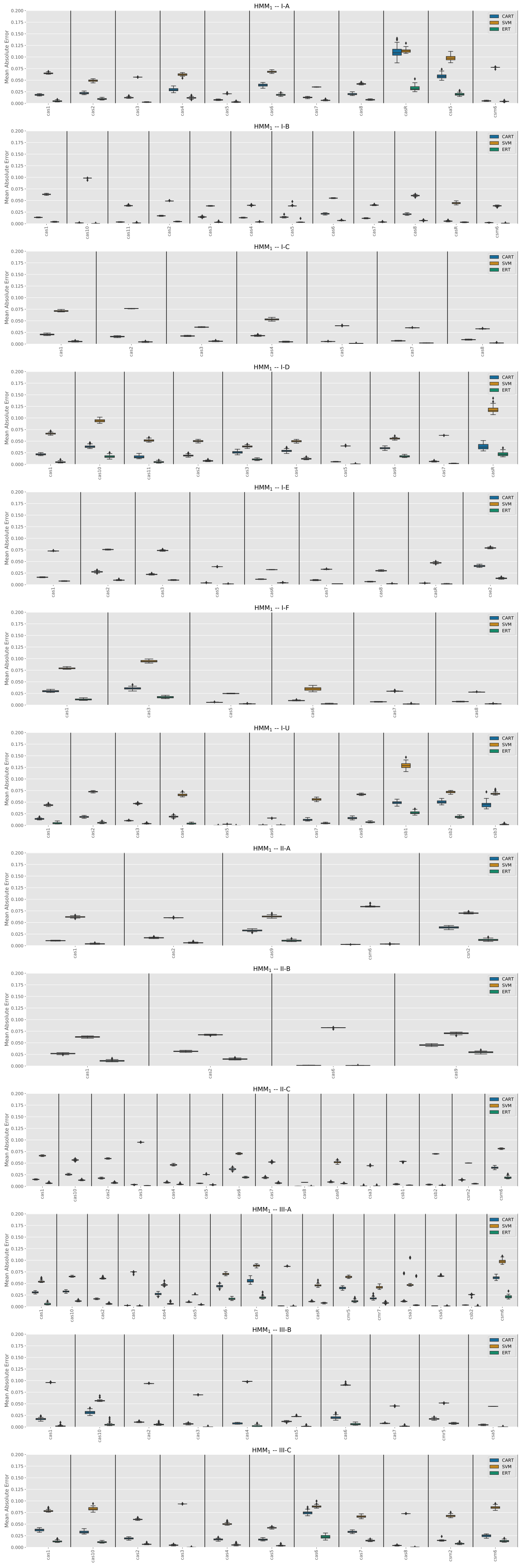

Figure S5: Mean absolute error results for all subtypes in HMM_1 over 50 nested cross-validation repetitions.

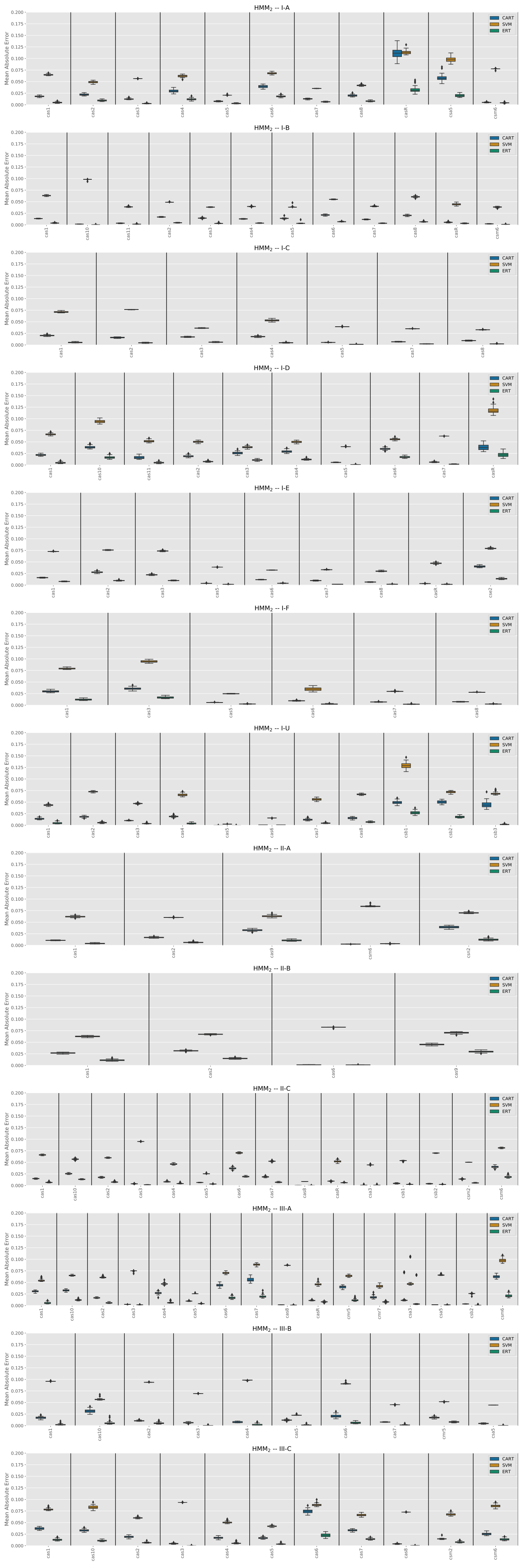

Figure S6: Mean absolute error results for all subtypes in HMM$_2$ over 50 nested cross-validation repetitions.

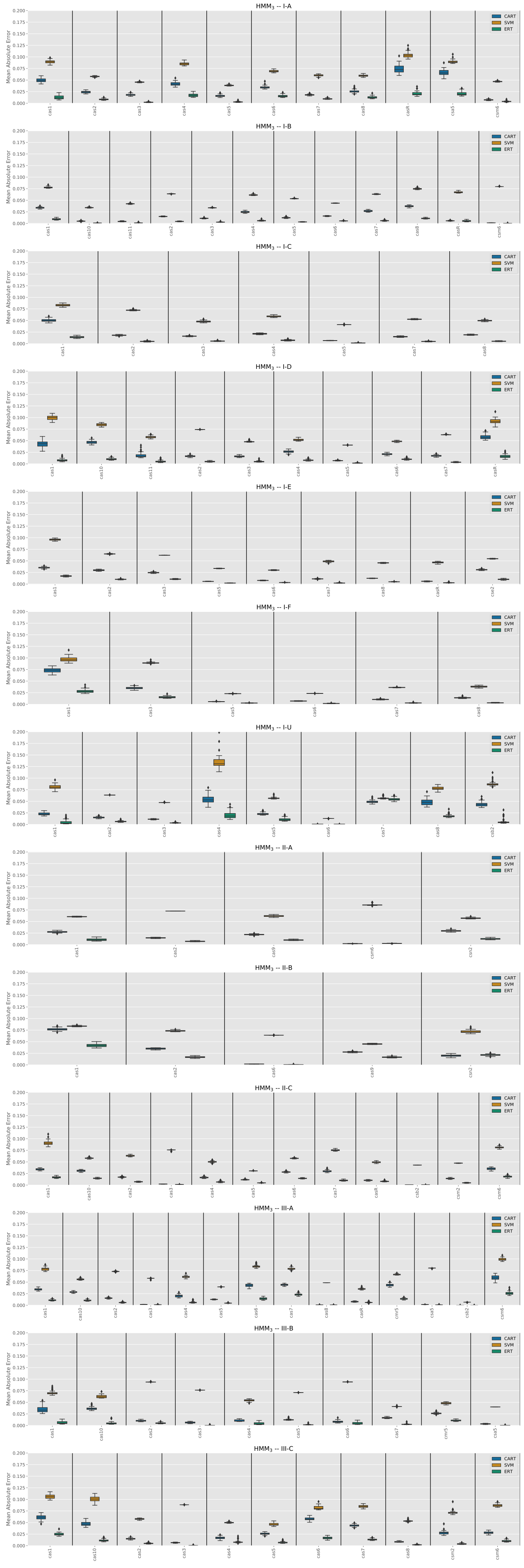

Figure S7: Mean absolute error results for all subtypes in HMM_3 over 50 nested cross-validation repetitions.

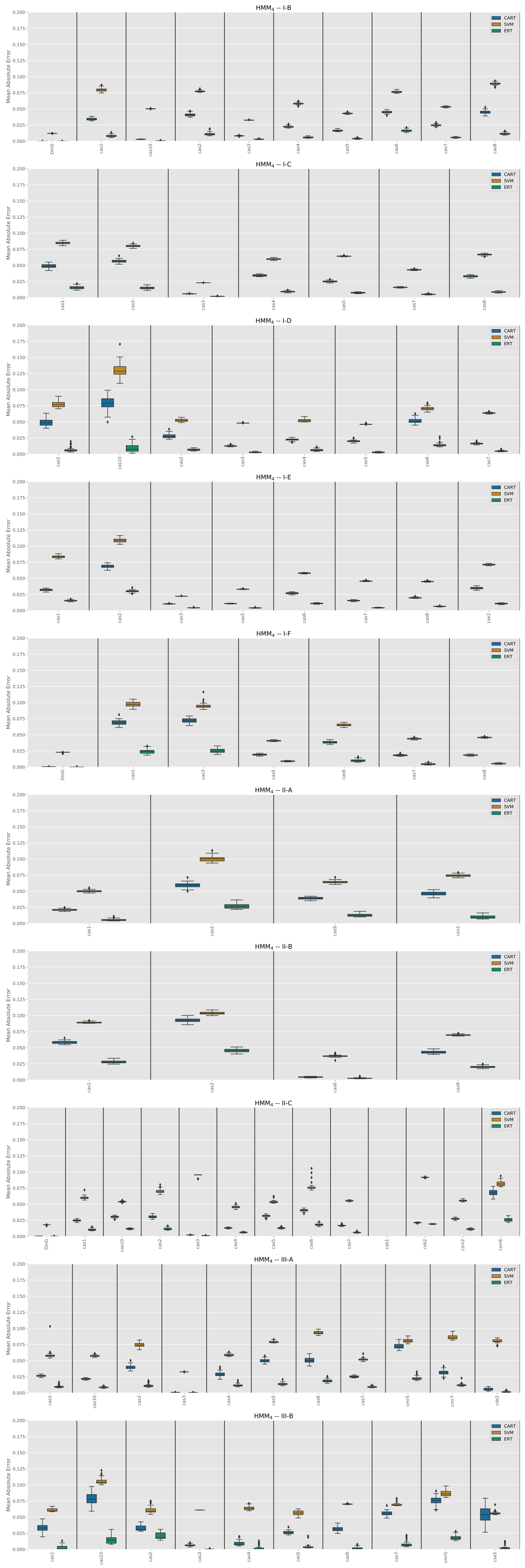

Figure S8: Mean absolute error results for all subtypes in HMM_4 over 50 nested cross-validation repetitions.

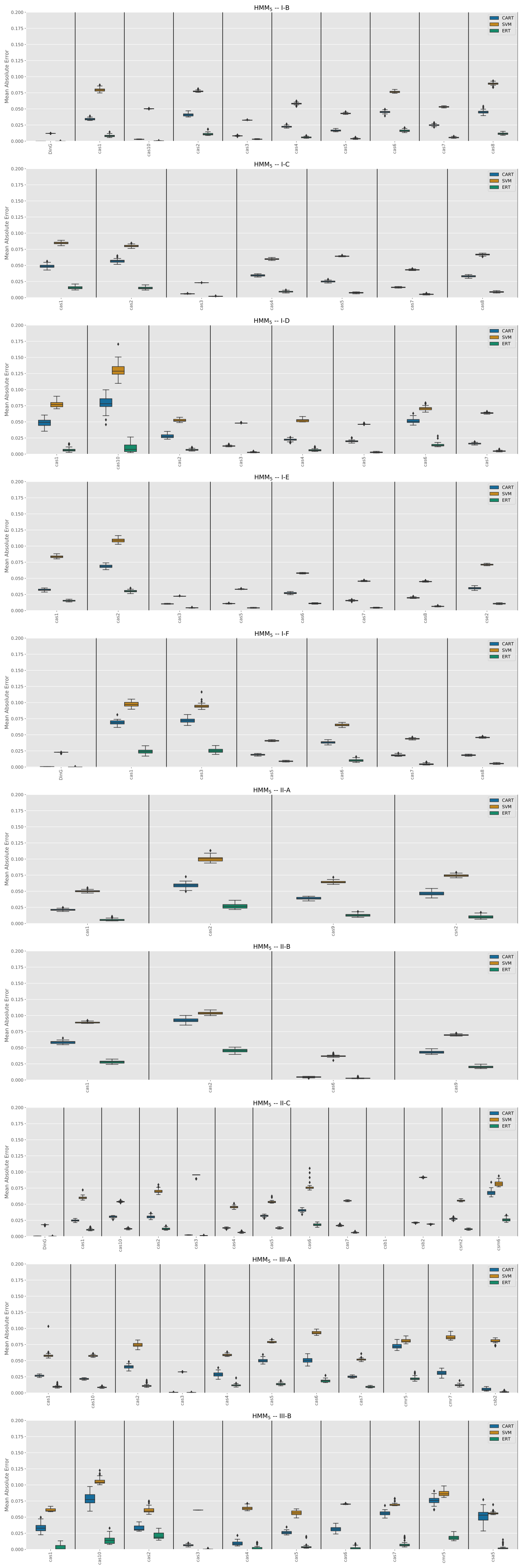

Figure S9: Mean absolute error results for all subtypes in HMM_5 over 50 nested cross-validation repetitions.

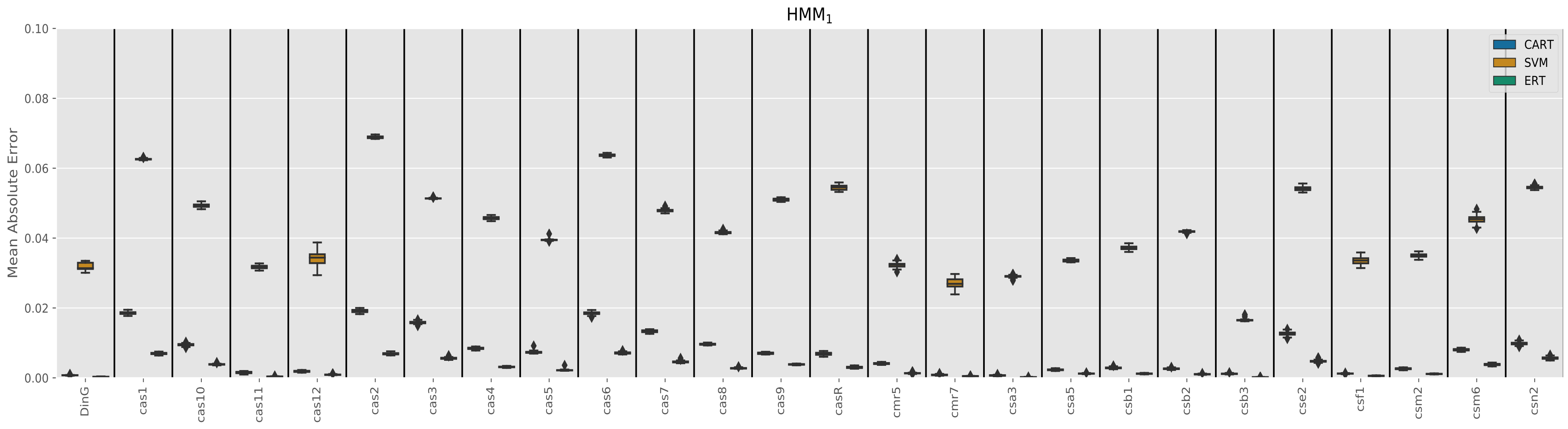

Figure S10: Mean absolute error results for the full HMM1 dataset (i.e., without separating by subtype) over 50 nested cross-validation repetitions.

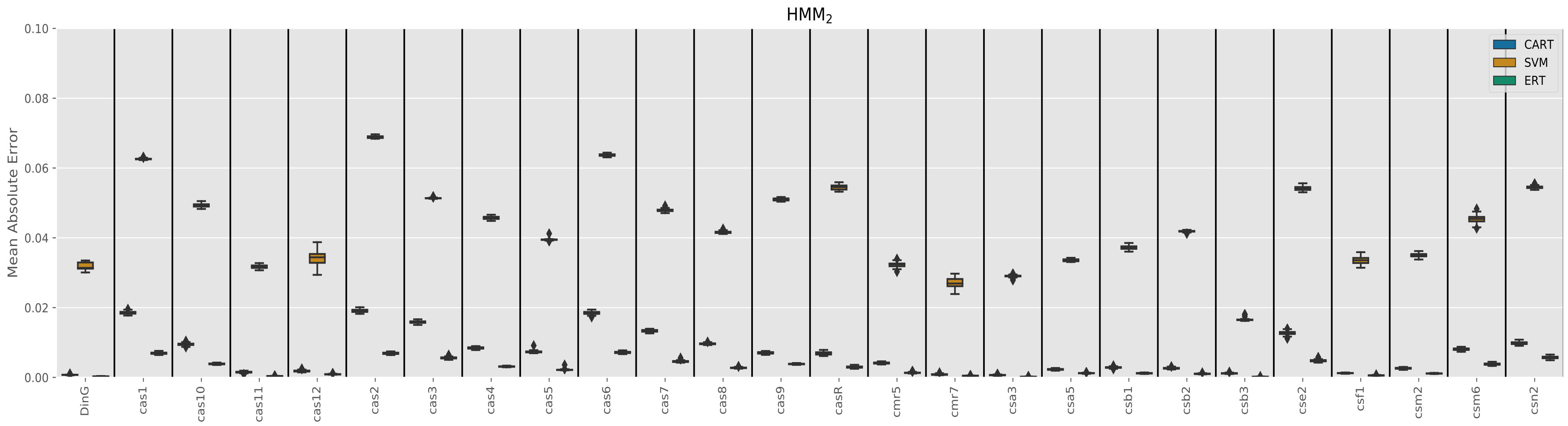

Figure S11: Mean absolute error results for the full HMM2 dataset (i.e., without separating by subtype) over 50 nested cross-validation repetitions.

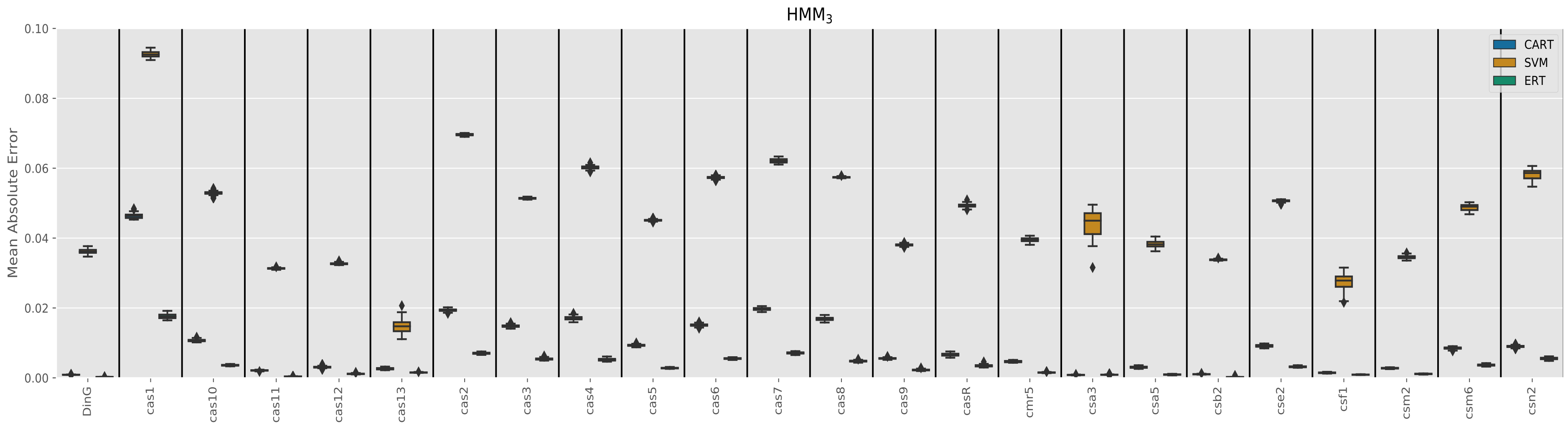

Figure S12: Mean absolute error results for the full HMM3 dataset (i.e., without separating by subtype) over 50 nested cross-validation repetitions.

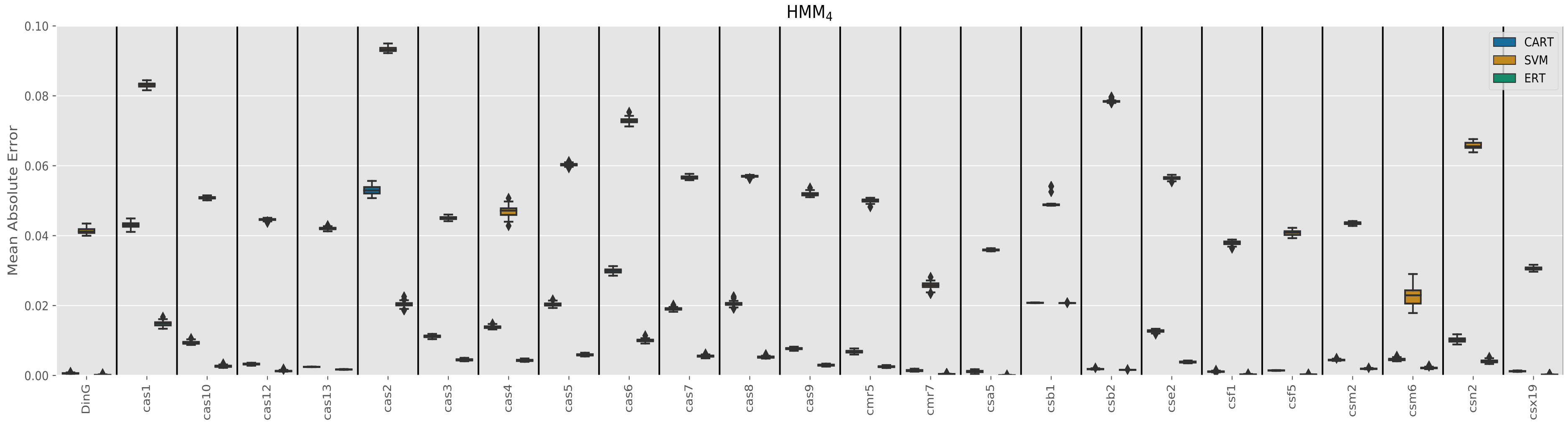

Figure S13: Mean absolute error results for the full HMM4 dataset (i.e., without separating by subtype) over 50 nested cross-validation repetitions.

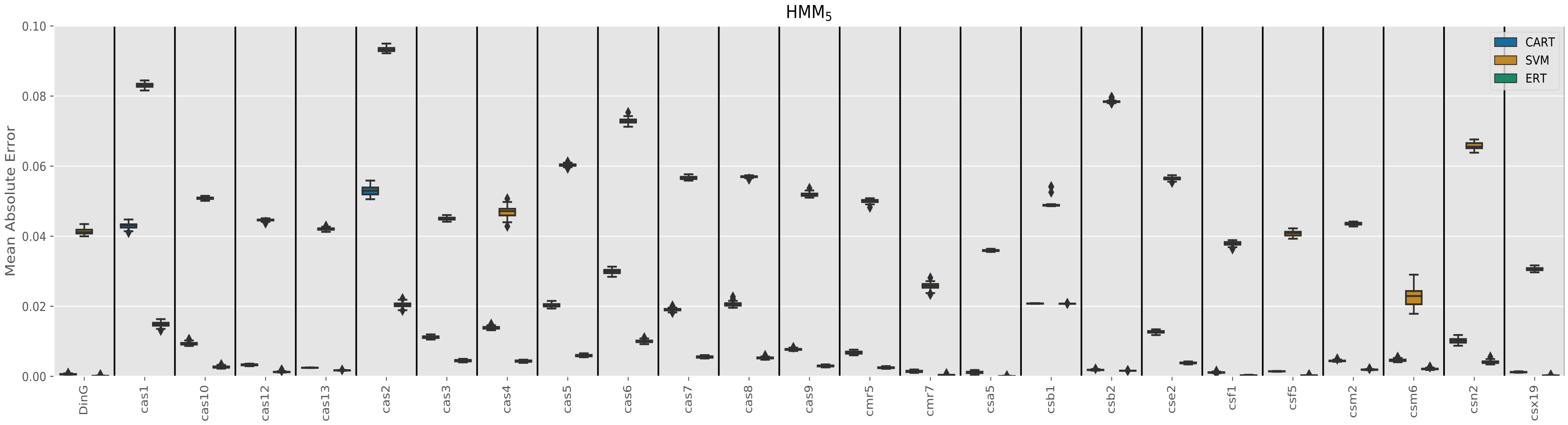

Figure S14: Mean absolute error results for the full HMM5 dataset (i.e., without separating by subtype) over 50 nested cross-validation repetitions.

In Tables S3–S18 we present all association rules found for the HMM1 dataset for each subtype separately.

Table S3: Rules generated for subtype I-A.

Target protein	Most important proteins
Cas3	(Cas8, 0.28), (Cas5, 0.24), (Cas7, 0.17), (Csa5, 0.09), (Casr, 0.06), (Cas6, 0.05), (Cas1, 0.04), (Cas4, 0.04), (Cas2, 0.03)
Cas8	(Cas3, 0.21), (Csa5, 0.19), (Casr, 0.18), (Cas5, 0.12), (Cas7, 0.10), (Cas2, 0.06), (Cas4, 0.05), (Cas1, 0.04), (Cas6, 0.04)
Cas7	(Casr, 0.31), (Csa5, 0.21), (Cas8, 0.18), (Cas5, 0.09), (Cas3, 0.07), (Cas1, 0.05), (Cas4, 0.04), (Cas6, 0.03), (Cas2, 0.02)
Cas5	(Casr, 0.30), (Csa5, 0.23), (Cas3, 0.13), (Cas8, 0.09), (Cas6, 0.09), (Cas7, 0.05), (Cas4, 0.04), (Cas1, 0.03), (Cas2, 0.03)
Cas6	(Cas3, 0.52), (Cas5, 0.17), (Cas2, 0.08), (Cas8, 0.07), (Csa5, 0.05), (Cas4, 0.03), (Cas7, 0.03), (Cas1, 0.03), (Casr, 0.02)
Cas1	(Casr, 0.44), (Csa5, 0.11), (Cas4, 0.10), (Cas7, 0.08), (Cas3, 0.08), (Cas8, 0.07), (Cas6, 0.06), (Cas2, 0.03), (Cas5, 0.03)
Cas2	(Cas4, 0.20), (Csa5, 0.16), (Cas7, 0.12), (Cas6, 0.11), (Casr, 0.10), (Cas8, 0.09), (Cas1, 0.08), (Cas3, 0.07), (Cas5, 0.07)
Cas4	(Cas2, 0.21), (Cas3, 0.18), (Cas1, 0.17), (Casr, 0.09), (Cas5, 0.08), (Cas8, 0.08), (Cas7, 0.07), (Csa5, 0.06), (Cas6, 0.05)
Csa5	(Csm6, 0.37), (Casr, 0.18), (Cas3, 0.10), (Cas7, 0.09), (Cas5, 0.08), (Cas6, 0.06), (Cas8, 0.05), (Cas2, 0.03), (Cas1, 0.02), (Cas4, 0.02)
Casr	(Csa5, 0.28), (Cas5, 0.16), (Cas6, 0.12), (Cas3, 0.12), (Cas8, 0.10), (Cas7, 0.09), (Cas2, 0.05), (Cas4, 0.05), (Cas1, 0.03)

Table S4: Rules generated for subtype I-B.

Target protein	Most important proteins
Cas3	(Cas5, 0.48), (Cas7, 0.19), (Cas8, 0.18), (Cas2, 0.05), (Cas4, 0.04), (Cas6, 0.03), (Cas1, 0.02)
Cas8	(Cas6, 0.28), (Cas3, 0.18), (Cas5, 0.18), (Cas7, 0.14), (Cas2, 0.08), (Cas1, 0.08), (Cas4, 0.06)
Cas7	(Cas3, 0.28), (Cas5, 0.18), (Cas6, 0.12), (Cas4, 0.12), (Cas8, 0.11), (Cas2, 0.10), (Cas1, 0.09)
Cas5	(Cas3, 0.54), (Cas7, 0.16), (Cas8, 0.11), (Cas6, 0.07), (Cas2, 0.05), (Cas4, 0.04), (Cas1, 0.03)
Cas6	(Cas8, 0.37), (Cas7, 0.18), (Cas3, 0.12), (Cas5, 0.11), (Cas2, 0.10), (Cas4, 0.06), (Cas1, 0.06)
Cas1	(Cas4, 0.87), (Cas7, 0.03), (Cas8, 0.03), (Cas3, 0.02), (Cas6, 0.02), (Cas2, 0.02)
Cas2	(Cas8, 0.19), (Cas5, 0.18), (Cas6, 0.14), (Cas7, 0.13), (Cas3, 0.12), (Cas4, 0.11), (Cas1, 0.11), (Casr, 0.02)
Cas4	(Cas1, 0.87), (Cas7, 0.05), (Cas3, 0.02), (Cas8, 0.02), (Cas6, 0.02)
Cas10	(Cas8, 0.28), (Cas5, 0.21), (Cas6, 0.18), (Cas1, 0.18), (Cas2, 0.15)
Casr	(Cas6, 0.23), (Cas8, 0.21), (Cas3, 0.19), (Cas5, 0.15), (Cas1, 0.08), (Cas7, 0.08), (Cas2, 0.03), (Cas4, 0.03)
Cas11	(Cas7, 0.28), (Cas6, 0.21), (Cas8, 0.19), (Cas5, 0.14), (Cas3, 0.09), (Cas4, 0.07), (Cas2, 0.02)
Csm6	(Cas7, 0.36), (Cas4, 0.35), (Cas3, 0.29)

Table S5: Rules generated for subtype I-C.

Target protein	Most important proteins
Cas3	(Cas7, 0.30), (Cas5, 0.18), (Cas8, 0.17), (Cas4, 0.12), (Cas2, 0.11), (Cas1, 0.11)
Cas8	(Cas7, 0.45), (Cas5, 0.29), (Cas3, 0.08), (Cas2, 0.07), (Cas4, 0.06), (Cas1, 0.04)
Cas7	(Cas5, 0.38), (Cas8, 0.26), (Cas3, 0.22), (Cas2, 0.05), (Cas1, 0.05), (Cas4, 0.04)
Cas5	(Cas7, 0.40), (Cas8, 0.25), (Cas3, 0.10), (Cas2, 0.10), (Cas4, 0.08), (Cas1, 0.07)
Cas1	(Cas4, 0.26), (Cas8, 0.19), (Cas2, 0.18), (Cas3, 0.17), (Cas7, 0.10), (Cas5, 0.09)
Cas2	(Cas5, 0.20), (Cas4, 0.19), (Cas1, 0.19), (Cas8, 0.16), (Cas3, 0.13), (Cas7, 0.12)
Cas4	(Cas2, 0.24), (Cas5, 0.18), (Cas1, 0.18), (Cas8, 0.16), (Cas7, 0.13), (Cas3, 0.12)

Table S6: Rules generated for subtype I-D.

Target protein	Most important proteins
Cas3	(Cas11, 0.48), (Cas10, 0.20), (Cas5, 0.11), (Cas7, 0.08), (Cas6, 0.04), (Cas4, 0.03), (Cas2, 0.03), (Cas1, 0.02)
Cas7	(Cas5, 0.56), (Cas3, 0.07), (Cas2, 0.07), (Casr, 0.07), (Cas6, 0.07), (Cas10, 0.06), (Cas1, 0.05), (Cas11, 0.03), (Cas4, 0.03)
Cas5	(Cas7, 0.66), (Cas10, 0.12), (Cas3, 0.07), (Cas6, 0.05), (Cas2, 0.04), (Cas4, 0.02), (Casr, 0.02), (Cas1, 0.02)
Cas6	(Cas3, 0.20), (Cas2, 0.19), (Cas10, 0.17), (Cas4, 0.14), (Cas7, 0.12), (Cas5, 0.10), (Casr, 0.03), (Cas1, 0.02)
Cas1	(Cas7, 0.36), (Cas5, 0.15), (Cas4, 0.15), (Cas10, 0.11), (Cas2, 0.08), (Cas3, 0.07), (Casr, 0.05), (Cas6, 0.04)
Cas2	(Cas10, 0.21), (Cas5, 0.20), (Cas3, 0.15), (Cas4, 0.14), (Cas6, 0.12), (Cas7, 0.08), (Casr, 0.05), (Cas1, 0.04)
Cas4	(Cas10, 0.24), (Cas3, 0.18), (Cas2, 0.11), (Cas7, 0.10), (Cas1, 0.10), (Casr, 0.09), (Cas5, 0.08), (Cas6, 0.06), (Cas11, 0.03)
Cas10	(Cas3, 0.28), (Cas5, 0.26), (Cas7, 0.17), (Cas4, 0.08), (Cas2, 0.06), (Cas6, 0.05), (Casr, 0.05), (Cas1, 0.04), (Cas11, 0.02)
Casr	(Cas5, 0.21), (Cas10, 0.18), (Cas2, 0.16), (Cas7, 0.14), (Cas1, 0.12), (Cas4, 0.09), (Cas3, 0.07), (Cas6, 0.04)
Cas11	(Cas3, 0.47), (Cas10, 0.36), (Cas5, 0.15)

Table S7: Rules generated for subtype I-E.

Target protein	Most important proteins
Cas3	(Cas8, 0.74), (Cse2, 0.08), (Cas7, 0.07), (Cas5, 0.04), (Cas1, 0.03), (Cas6, 0.02)
Cas8	(Cas3, 0.68), (Cse2, 0.13), (Cas5, 0.07), (Cas7, 0.05), (Cas1, 0.03), (Cas6, 0.03), (Cas2, 0.02)
Cse2	(Cas7, 0.25), (Cas5, 0.23), (Cas8, 0.19), (Cas1, 0.13), (Cas3, 0.10), (Cas6, 0.08), (Cas2, 0.02)
Cas7	(Cas6, 0.21), (Cas8, 0.18), (Cse2, 0.16), (Cas3, 0.14), (Cas1, 0.13), (Cas5, 0.11), (Cas2, 0.06)
Cas5	(Cse2, 0.19), (Cas1, 0.19), (Cas6, 0.18), (Cas7, 0.14), (Cas8, 0.13), (Cas2, 0.09), (Cas3, 0.08)
Cas6	(Cas5, 0.26), (Cas2, 0.16), (Cas7, 0.16), (Cas1, 0.15), (Cse2, 0.12), (Cas8, 0.10), (Cas3, 0.05)
Cas1	(Cas7, 0.21), (Cas5, 0.20), (Cse2, 0.18), (Cas3, 0.13), (Cas8, 0.13), (Cas6, 0.10), (Cas2, 0.06)
Cas2	(Cas6, 0.24), (Cas5, 0.19), (Cas7, 0.14), (Cas8, 0.12), (Cas1, 0.12), (Cse2, 0.11), (Cas3, 0.08)
Casr	(Cas1, 0.33), (Cas7, 0.25), (Cas8, 0.21), (Cas5, 0.06), (Cas2, 0.04), (Cas6, 0.04), (Cse2, 0.03), (Cas3, 0.03)

Table S8: Rules generated for subtype I-F.

Target protein	Most important proteins
Cas3	(Cas8, 0.39), (Cas5, 0.29), (Cas7, 0.15), (Cas1, 0.10), (Cas6, 0.07)
Cas8	(Cas5, 0.67), (Cas3, 0.10), (Cas7, 0.09), (Cas6, 0.07), (Cas1, 0.07)
Cas7	(Cas8, 0.28), (Cas5, 0.20), (Cas6, 0.19), (Cas1, 0.18), (Cas3, 0.16)
Cas5	(Cas8, 0.65), (Cas6, 0.13), (Cas7, 0.11), (Cas1, 0.07), (Cas3, 0.04)
Cas6	(Cas7, 0.28), (Cas8, 0.24), (Cas5, 0.23), (Cas1, 0.20), (Cas3, 0.06)
Cas1	(Cas7, 0.36), (Cas8, 0.19), (Cas5, 0.18), (Cas3, 0.14), (Cas6, 0.13)

Table S9: Rules generated for subtype I-U.

Target protein	Most important proteins
Cas3	(Csb2, 0.53), (Cas8, 0.33), (Csb1, 0.05), (Cas7, 0.03), (Cas5, 0.02)
Cas8	(Cas3, 0.53), (Csb1, 0.18), (Csb2, 0.15), (Cas7, 0.06), (Cas2, 0.04), (Cas4, 0.03), (Cas1, 0.02)
Cas7	(Csb2, 0.43), (Cas8, 0.29), (Cas3, 0.24), (Cas2, 0.03)
Cas6	(Cas8, 0.28), (Cas3, 0.28), (Csb2, 0.24), (Csb1, 0.20)
Cas1	(Cas4, 0.85), (Cas3, 0.06), (Csb2, 0.04), (Cas8, 0.02), (Cas2, 0.02)
Cas2	(Csb3, 0.34), (Cas1, 0.22), (Cas4, 0.19), (Cas8, 0.14), (Cas3, 0.05), (Csb2, 0.04)
Cas4	(Cas1, 0.86), (Cas2, 0.06), (Cas3, 0.03), (Cas8, 0.02), (Csb2, 0.02)
Csb2	(Cas3, 0.72), (Cas8, 0.17), (Csb1, 0.02), (Cas2, 0.02), (Cas1, 0.02), (Cas4, 0.02)
Csb1	(Cas8, 0.62), (Cas3, 0.11), (Cas2, 0.11), (Csb3, 0.07), (Csb2, 0.06), (Cas1, 0.02)
Csb3	(Cas2, 0.33), (Cas1, 0.17), (Csb1, 0.17), (Cas4, 0.12), (Csb2, 0.12), (Cas3, 0.08)

Table S10: Rules generated for subtype II-A.

Target protein	Most important proteins
Cas1	(Csn2, 0.42), (Cas9, 0.36), (Cas2, 0.23)
Cas2	(Cas9, 0.55), (Csn2, 0.27), (Cas1, 0.18)
Cas9	(Cas1, 0.41), (Cas2, 0.32), (Csn2, 0.27)
Csn2	(Cas1, 0.62), (Cas9, 0.23), (Cas2, 0.15)

Table S11: Rules generated for subtype II-B.

Target protein	Most important proteins
Cas1	(Cas9, 0.88), (Cas4, 0.07), (Cas2, 0.05)
Cas2	(Cas9, 0.46), (Cas4, 0.41), (Cas1, 0.14)
Cas4	(Cas9, 0.83), (Cas1, 0.09), (Cas2, 0.08)
Cas9	(Cas1, 0.66), (Cas4, 0.32), (Cas2, 0.02)

Table S12: Rules generated for subtype II-C.

Target protein	Most important proteins
Cas6	(Cas2, 0.37), (Cas1, 0.36), (Cas9, 0.27)
Cas1	(Cas9, 0.75), (Cas2, 0.25)
Cas2	(Cas1, 0.59), (Cas9, 0.41)
Cas9	(Cas1, 0.68), (Cas2, 0.32)

Table S13: Rules generated for subtype III-A.

Target protein	Most important proteins
Cas3	(Csb2, 0.23), (Csb1, 0.22), (Cas7, 0.21), (Cas4, 0.13), (Cas2, 0.07), (Cas6, 0.06), (Cas1, 0.04), (Casr, 0.02), (Cas8, 0.02)
Cas7	(Cas5, 0.38), (Cas10, 0.19), (Cas6, 0.13), (Csm2, 0.11), (Cas2, 0.08), (Cas1, 0.05), (Csm6, 0.03)
Cas5	(Cas6, 0.27), (Cas10, 0.25), (Cas7, 0.16), (Csm2, 0.12), (Cas2, 0.08), (Cas1, 0.06), (Csm6, 0.06)
Cas6	(Cas10, 0.27), (Csm6, 0.25), (Cas5, 0.17), (Csm2, 0.13), (Cas2, 0.12), (Cas7, 0.04), (Cas1, 0.02)
Cas1	(Cas4, 0.25), (Cas2, 0.19), (Cas5, 0.13), (Csm2, 0.11), (Cas10, 0.08), (Cas7, 0.08), (Cas6, 0.08), (Csm6, 0.04), (Casr, 0.02)
Cas2	(Csm6, 0.38), (Cas6, 0.21), (Cas10, 0.11), (Cas7, 0.09), (Csm2, 0.08), (Cas1, 0.07), (Cas5, 0.05)
Cas4	(Cas1, 0.73), (Cas7, 0.08), (Cas6, 0.05), (Cas5, 0.04), (Cas2, 0.04), (Cas10, 0.02), (Csm2, 0.02)
Cas10	(Cas5, 0.40), (Cas6, 0.24), (Csm2, 0.10), (Cas7, 0.09), (Csm6, 0.07), (Cas2, 0.04), (Cas1, 0.03)
Casr	(Cas2, 0.27), (Cas3, 0.22), (Cas8, 0.19), (Cas6, 0.10), (Cas1, 0.07), (Cas10, 0.07), (Cas4, 0.06), (Cas5, 0.02), (Csm2, 0.02)
Csm6	(Cas6, 0.48), (Cas7, 0.19), (Cas2, 0.15), (Cas10, 0.08), (Csm2, 0.05), (Cas5, 0.03)
Csm2	(Cas5, 0.27), (Cas6, 0.22), (Cas10, 0.16), (Cas7, 0.12), (Cas2, 0.07), (Csm6, 0.06), (Cas1, 0.05)

Table S14: Rules generated for subtype III-B.

Target protein	Most important proteins
Cas3	(Casr, 0.19), (Cas7, 0.16), (Cmr5, 0.16), (Csm6, 0.15), (Cas1, 0.15), (Cas4, 0.12), (Cas2, 0.07)
Cas7	(Cas10, 0.24), (Cas2, 0.21), (Cas5, 0.19), (Cmr5, 0.12), (Csm6, 0.09), (Cas6, 0.08), (Cas1, 0.06)
Cas5	(Cmr5, 0.32), (Cas10, 0.24), (Cas7, 0.21), (Cas2, 0.07), (Cas6, 0.05), (Cas1, 0.04), (Csm6, 0.03), (Cas4, 0.02)
Cas6	(Cas1, 0.79), (Cas2, 0.07), (Cmr5, 0.05), (Cas5, 0.04), (Cas10, 0.02), (Cas7, 0.02)
Cas1	(Cas6, 0.84), (Csb2, 0.04), (Cas4, 0.04), (Cas2, 0.04)
Cas2	(Cas7, 0.20), (Cas5, 0.18), (Cas6, 0.16), (Cas10, 0.13), (Cas1, 0.11), (Cmr5, 0.10), (Csm6, 0.07), (Cas4, 0.03), (Csa3, 0.02)
Cas4	(Csb2, 0.67), (Cas1, 0.22), (Cmr5, 0.03), (Cas2, 0.03), (Cas7, 0.02)
Cas10	(Cas7, 0.31), (Cas5, 0.22), (Cas6, 0.16), (Cmr5, 0.11), (Cas1, 0.08), (Cmr7, 0.06), (Cas2, 0.04), (Csm6, 0.03)
Casr	(Cmr5, 0.21), (Cas7, 0.17), (Cas3, 0.16), (Csm6, 0.14), (Cas10, 0.13), (Cas5, 0.07), (Cas1, 0.05), (Cas4, 0.05)
Csm6	(Cmr5, 0.27), (Cas5, 0.26), (Cas10, 0.20), (Cas7, 0.12), (Cas6, 0.08), (Cas1, 0.03), (Cas2, 0.02)
Cmr5	(Cas5, 0.32), (Cas7, 0.21), (Cas10, 0.17), (Cas1, 0.13), (Csm6, 0.10), (Cas2, 0.04), (Cas6, 0.02)
Cmr7	(Cas7, 0.33), (Cmr5, 0.30), (Cas10, 0.21), (Cas5, 0.16)
Csa3	(Cas7, 0.28), (Cas6, 0.16), (Cmr5, 0.12), (Cas4, 0.12), (Cas1, 0.12), (Cas5, 0.12), (Cas2, 0.08)

Table S15: Rules generated for subtype III-C.

Target protein	Most important proteins
Cas7	(Cas10, 0.46), (Cas5, 0.38), (Cas2, 0.05), (Cmr5, 0.04), (Cas1, 0.04), (Cas6, 0.02)
Cas5	(Cas3, 0.31), (Csa5, 0.27), (Cmr5, 0.16), (Cas7, 0.13), (Cas10, 0.06), (Cas2, 0.03), (Cas6, 0.02)
Cas6	(Cas1, 0.33), (Cas10, 0.31), (Cas7, 0.16), (Cmr5, 0.09), (Cas2, 0.05), (Cas5, 0.04), (Cas4, 0.02)
Cas1	(Cas6, 0.49), (Cas4, 0.20), (Cas2, 0.10), (Cas7, 0.06), (Cas10, 0.06), (Cmr5, 0.06), (Cas5, 0.02)
Cas2	(Cas7, 0.29), (Cas6, 0.23), (Cas10, 0.17), (Cas1, 0.12), (Cas4, 0.09), (Cas5, 0.07), (Cmr5, 0.02)
Cas4	(Cas3, 0.19), (Cas7, 0.16), (Csa5, 0.13), (Cas5, 0.13), (Cas6, 0.12), (Cmr5, 0.10), (Cas1, 0.09), (Cas10, 0.08)
Cas10	(Cas7, 0.46), (Cas5, 0.29), (Cmr5, 0.14), (Cas6, 0.05), (Cas2, 0.03), (Cas1, 0.02)
Cmr5	(Cas5, 0.34), (Cas10, 0.25), (Cas7, 0.17), (Cas2, 0.09), (Cas6, 0.08), (Cas1, 0.06)

Table S16: Rules generated for subtype III-D.

Target protein	Most important proteins
Cas3	(Cas6, 0.20), (Cas4, 0.13), (Cas2, 0.13), (Cas1, 0.13), (Cas8, 0.13), (Cas10, 0.10), (Cas7, 0.10), (Cas5, 0.08)
Cas8	(Cas3, 0.51), (Cas5, 0.41), (Cas2, 0.06)
Cas7	(Cas10, 0.36), (Csm2, 0.27), (Csm6, 0.15), (Cas5, 0.10), (Cas6, 0.04), (Cas1, 0.03), (Cas2, 0.03)
Cas5	(Cas6, 0.33), (Cas1, 0.25), (Cas2, 0.15), (Cas7, 0.09), (Cas10, 0.06), (Cas3, 0.03), (Cas4, 0.03), (Csm6, 0.02)
Cas6	(Cas10, 0.34), (Cas1, 0.32), (Cas7, 0.09), (Csm6, 0.08), (Csm2, 0.07), (Cas5, 0.06), (Cas2, 0.02)
Cas1	(Cas6, 0.48), (Cas5, 0.26), (Cas4, 0.10), (Cas7, 0.06), (Cas10, 0.05), (Cas2, 0.04)
Cas2	(Cas10, 0.17), (Csm2, 0.15), (Cas7, 0.13), (Cas5, 0.13), (Csm6, 0.10), (Cas8, 0.09), (Cas1, 0.08), (Cas4, 0.07), (Cas6, 0.07), (Cas3, 0.02)
Cas4	(Cas1, 0.48), (Cas6, 0.26), (Cas2, 0.12), (Cas7, 0.07), (Cas5, 0.04)
Cas10	(Csm2, 0.50), (Cas7, 0.21), (Cas5, 0.14), (Cas6, 0.09), (Csm6, 0.04)
Csm6	(Cas5, 0.26), (Cas2, 0.19), (Cas10, 0.18), (Cas1, 0.17), (Cas7, 0.08), (Cas6, 0.07), (Csm2, 0.05)
Csm2	(Cas6, 0.47), (Cas10, 0.33), (Cas7, 0.12), (Cas5, 0.04), (Csm6, 0.03)

Table S17: Rules generated for subtype IV-A.

Target protein	Most important proteins
Cas7	(Ding, 0.34), (Csf1, 0.28), (Cas5, 0.20), (Cas6, 0.18)
Cas5	(Csf1, 0.46), (Cas7, 0.30), (Ding, 0.19), (Cas6, 0.04)
Cas6	(Cas7, 0.68), (Csf1, 0.20), (Cas5, 0.12)
Ding	(Cas7, 0.64), (Csf1, 0.26), (Cas5, 0.10)
Csf1	(Ding, 0.33), (Cas7, 0.30), (Cas5, 0.25), (Cas6, 0.11)

Table S18: Rules generated for subtype V-A.

Target protein	Most important proteins
Cas1	(Cas2, 0.64), (Cas4, 0.25), (Cas12, 0.11)
Cas2	(Cas1, 0.54), (Cas4, 0.38), (Cas12, 0.08)
Cas4	(Cas1, 0.63), (Cas2, 0.33), (Cas12, 0.04)
Cas12	(Cas2, 0.50), (Cas1, 0.30), (Cas4, 0.20)

Table S19. Summary of the compared tools.

	Identification of Cas proteins	Classification of complete cassettes	Identification of missing Cas proteins	Classification of incomplete cassettes	Learns association rules	Handles unseen cassettes	Method	Input
CRISPRone	Yes	Yes	No	No	No	No	HMM and HMMER tool	DNA only
HmmCas	Yes	No	No	No	No	No	HMM and HMMER tool	Cassette of proteins
CRISPRminer	Yes	Yes	No	No	No	No	HMM and CRISPRcasFinder	DNA only
CRISPRminer2	Yes	Yes	No	No	No	No	HMM, PSI-Blast and CRISPRcasFinder	DNA only
Macsyfinder	Yes	Yes	No	No	No	No	HMM and HMMER tool	Proteins
CRISPRcasFinder	Yes	Yes	No	No	No	No	HMM and Macsyfinder	DNA only
CRISPRdisco	Yes	Yes	No	No	No	No	PSI-Blast	DNA and Proteins
CRISPRcasIdentifier	Yes	Yes	Yes	Yes	Yes	Yes	HMM and 3 different ML approaches	DNA or Proteins

Table S20: Example for using non-custom HMM models such as PFAM or TIGRFAM

I-A proteins	# of proteins[2]	Our tool	TIGRFAMs	PFam.
cas1	36	36	36	36
cas2	39	39	39	18
cas3	85	85	52	25
cas4	70	70	62	45
cas5	49	49	31	25
cas6	38	38	30	17
cas7	117	117	53	53
cas8	59	55	19	14

References

Cherkassky, V. and Dhar, S. (2010). Simple method for interpretation of high-dimensional nonlinear svm classification models. In *6th International Conference on Data Mining*, pages 267–272.

Makarova, K. S., Wolf, Y. I., Alkhnbashi, O. S., Costa, F., Shah, S. A., Saunders, S. J., Barrangou, R., Brouns, S. J. J., Charpentier, E., Haft, D. H., Horvath, P., Moineau, S., Mojica, F. J. M., Terns, R. M., Terns, M. P., White, M. F., Yakunin, A. F., Garrett, R. A., van der Oost, J., Backofen, R., and Koonin, E. V. (2015). An updated evolutionary classification of CRISPR-Cas systems. *Nature Reviews Microbiology*, **13**(11), 722–736.
