# Supplementary material for: CRISPRcasIdentifier: Machine learning for accurate identification and classification of CRISPR-Cas systems
Source: Gigascience. 2020 Jun 17;9(6):giaa062. doi: 10.1093/gigascience/giaa062 (PMC7298778; doi:10.1093/gigascience/giaa062)
Supplement: giaa062_Response_to_Reviewer_Comments_Original_Submission [file giaa062_response_to_reviewer_comments_original_submission.pdf]

Author's Response To Reviewer Comments

Close

GigaScience

Dear Dr. Hans Zauner

Herewith we submit the revised version of our manuscript

CRISPRcasIdentifier: Machine learning for accurate identification and classification of CRISPR-Cas systems

We have answered each issue and have modified our submission where necessary. We have taken careful consideration of the reviewers' comments and have integrated them into a thoroughly revised version of the manuscript. Additional to the reviewers comments, we took your suggestion into consideration. We are grateful for the constructive comments that have significantly improved the presentation of our work.

Please find a point-by-point response to the reviews below.

I am looking forward to hearing from you.

Sincerely yours,
Prof. Dr. Rolf Backofen

Point-to-point response to reviewer comments:

Editor suggestion

1- I particularly agree with reviewer 1 that clarifying all data sources and improving the "readme" are two important points to address. Please also consider the suggestion of reviewer 2 to provide a dockerized version for end users' convenience. In addition, please register any new software application in the bio.tools and SciCrunch.org databases to receive RRID (Research Resource Identification Initiative ID) and biotoolsID identifiers, and include these in your manuscript. This will facilitate tracking, reproducibility and re-use of your tool

Authors' response: First, we better clarified the data sources by providing one extra table in our GitHub repository (https://github.com/BackofenLab/CRISPRcasIdentifier, see Supplementary_files folder under

the GitHub) which lists all CRISPR cassettes used and from which source each one of them came from. In addition, our repository includes a detailed README.md file (which describes the installation process and usage) and we also created a dockerized version (https://hub.docker.com/repository/docker/padilha/crispr-cas-identifier), as suggested by reviewer #2. Finally, we also registered our tool in SciCrunch.org (RRID: SCR_018296) and bio.tools (biotoolsID: crisprcasidentifier) and included the respective IDs in the “Availability of Source Code and Requirements” section.

Reviewer #1: The computational tool that the authors present for identifying CRISPR-Cas systems can potentially save researchers time and effort as well as assisting in identifying novel Cas proteins. Although not the first tool in this space, CRISPRCasIdentifier does appear to outperform pre-existing tools by a large margin. Modelling is based on a good-sized dataset and the ML component appropriately features cross-validation and tuning. The paper is well-written with minimal grammatical errors.

Manuscript comments:
1- I understand that the dataset appears to be from multiple sources with a total of 68594 samples. Is this the data from [2,3,4]? A table (perhaps in supplementary) outlining how much and from where all of the data comes from would help clarify this.

Authors’ response: Done. Please see the supplementary file named CRISPR_cassette_source.tab in our GitHub repository (https://github.com/BackofenLab/CRISPRcasIdentifier).

2 - Although both terms are appropriate, "trained" seems to be more common vocabulary than "induced" based on a quick literature search of recent manuscripts. I don't believe all instances of induced in the manuscript must be replaced with trained, but I do believe trained sounds more natural so should perhaps be considered. I.e. in the abstract "models induced by using machine learning" compared with "models trained using machine learning".

Authors’ response: Thank you for the suggestion, we changed all occurrences of the word “induce” to “train” in the text.

3 - Please check the grammar of the following phrase in "Data collecting and preprocessing" section: "is a pair of the genomic sequence containing all genes of the cassette".

Authors’ response: We checked the grammar and corrected the sentence.

4 - Should the title of that section be "Data collection and preprocessing"?

Authors’ response: We agree with the reviewer and changed the title of the section.

5 - Is hyphenation intended in this sentence? "and as a result re-assure key components of subtypes like"

Authors’ response: The correct term is reassure. We corrected it in the text.

6- I notice the project web page is password-protected, although the password is provided in the manuscript. Is password-protection a temporary measure until the paper is accepted?
I believe it would be more user-friendly to have the project available as a GitHub repository with README.md available upfront, rather then requiring the user to follow one set of instructions before being able to view it.
Also, as I'm sure you know, this has the benefit of tagged releases for better reproducibility, given the inevitable bugfixes/improvements of bioinformatics tools.

Authors’ response: Yes, the password protection was a temporary measure for the review process. We moved the code to a public GitHub repository. It is now available with a proper README.md file in: https://github.com/BackofenLab/CRISPRcasIdentifier. We also created a tagged release for the version

we used in our paper.

7- Program comments:
* Biopython should be listed as a requirement in README.md
* The examples in README.md on lines 78 and 79 contain "fasta" extensions but the respective files in the "examples" directory have a "fa" extension.
* The program looks for "models" directory or "models.tar.gz" archive. If I follow the instructions I end up with a "trained_models_2015" directory. Therefore the program fails unless this directory is renamed.

Authors’ response: We thank the reviewer for all the above comments. We corrected them as suggested.

Reviewer #2:

The manuscript is well written and the conclusion is backed up by a comprehensive banchmark and data data collection. The tools are available and the code is documented.

My only concern is that my Mac had a hard time resolving the conda environment. More testing of the boiler plate code to allow others to deploy your methods is warranted prior to publication. (a docker container based solution through docker hub might be quick to implement and make things easier for end-users)

Despite this, I think the manuscript describes an elegant solution to a timely problem that may likely help many researches to find and understand CRISPR systems in more organisms.

Authors’ response: We would like to thank the reviewer for this comment. We now provide the users with three options to install our tool:
They may use a DockerHub image, available in https://hub.docker.com/repository/docker/padilha/crispr-cas-identifier;
They may build our docker image from the Dockerfile available in our GitHub repository (https://github.com/BackofenLab/CRISPRcasIdentifier); or
They may set a conda environment up from our crispr-env.yml file, also available in our Github repository.

In the README.md file we explain each of the aforementioned options in detail.

Close
