# Supplementary material for: CRISPRcasIdentifier: Machine learning for accurate identification and classification of CRISPR-Cas systems
Source: Gigascience. 2020 Jun 17;9(6):giaa062. doi: 10.1093/gigascience/giaa062 (PMC7298778; doi:10.1093/gigascience/giaa062)
Supplement: giaa062_Response_to_Reviewer_Comments_Revision_1 [file giaa062_response_to_reviewer_comments_revision_1.pdf]

Author's Response To Reviewer Comments

Close

We included the GigaDB information as requested by the editor.
We also added a reference to the GigaDB in the paper in the Data Availability section.
We removed all highlights in yellow from the paper.

Close
