# Supplementary material for: CRISPRcasIdentifier: Machine learning for accurate identification and classification of CRISPR-Cas systems
Source: Gigascience. 2020 Jun 17;9(6):giaa062. doi: 10.1093/gigascience/giaa062 (PMC7298778; doi:10.1093/gigascience/giaa062)
Supplement: giaa062_Reviewer_1_Report_Original_Submission — Aidan O'Brien -- 4/1/2020 Reviewed [file giaa062_reviewer_1_report_original_submission.pdf]

Reviewer Report

Title: CRISPRcasIdentifier: Machine learning for accurate identification and classification of CRISPR-Cas systems

Version: Original Submission **Date:** 4/1/2020

Reviewer name: Aidan O'Brien

Reviewer Comments to Author:

The computational tool that the authors present for identifying CRISPR-Cas systems can potentially save researchers time and effort as well as assisting in identifying novel Cas proteins. Although not the first tool in this space, CRISPRCasIdentifier does appear to outperform pre-existing tools by a large margin. Modelling is based on a good-sized dataset and the ML component appropriately features cross-validation and tuning. The paper is well-written with minimal grammatical errors.
Manuscript comments:

- I understand that the dataset appears to be from multiple sources with a total of 68594 samples. Is this the data from [2,3,4]? A table (perhaps in supplementary) outlining how much and from where all of the data comes from would help clarify this.
- Although both terms are appropriate, "trained" seems to be more common vocabulary than "induced" based on a quick literature search of recent manuscripts. I don't believe all instances of induced in the manuscript must be replaced with trained, but I do believe trained sounds more natural so should perhaps be considered. I.e. in the abstract "models induced by using machine learning" compared with "models trained using machine learning".
- Please check the grammar of the following phrase in "Data collecting and preprocessing" section: "is a pair of the genomic sequence containing all genes of the cassette".
- Should the title of that section be "Data collection and preprocessing"?
- Is hyphenation intended in this sentence? "and as a result re-assure key components of subtypes like"

I notice the project web page is password-protected, although the password is provided in the manuscript. Is password-protection a temporary measure until the paper is accepted?
I believe it would be more user-friendly to have the project available as a GitHub repository with README.md available upfront, rather then requiring the user to follow one set of instructions before being able to view it.
Also, as I'm sure you know, this has the benefit of tagged releases for better reproducibility, given the inevitable bugfixes/improvements of bioinformatics tools.
Program comments:

- Biopython should be listed as a requirement in README.md
- The examples in README.md on lines 78 and 79 contain "fasta" extensions but the respective files in the "examples" directory have a "fa" extension.
- The program looks for "models" directory or "models.tar.gz" archive. If I follow the instructions I end up with a "trained_models_2015" directory. Therefore the program fails unless this directory is

renamed.

Level of Interest

Please indicate how interesting you found the manuscript: Choose an item.

Quality of Written English

Please indicate the quality of language in the manuscript: Choose an item.

Declaration of Competing Interests

Please complete a declaration of competing interests, considering the following questions:

- Have you in the past five years received reimbursements, fees, funding, or salary from an organisation that may in any way gain or lose financially from the publication of this manuscript, either now or in the future?
- Do you hold any stocks or shares in an organisation that may in any way gain or lose financially from the publication of this manuscript, either now or in the future?
- Do you hold or are you currently applying for any patents relating to the content of the manuscript?
- Have you received reimbursements, fees, funding, or salary from an organization that holds or has applied for patents relating to the content of the manuscript?
- Do you have any other financial competing interests?
- Do you have any non-financial competing interests in relation to this paper?

If you can answer no to all of the above, write 'I declare that I have no competing interests' below. If your reply is yes to any, please give details below.

I declare that I have no competing interests

I agree to the open peer review policy of the journal. I understand that my name will be included on my report to the authors and, if the manuscript is accepted for publication, my named report including any attachments I upload will be posted on the website along with the authors' responses. I agree for my report to be made available under an Open Access Creative Commons CC-BY license (http://creativecommons.org/licenses/by/4.0/). I understand that any comments which I do not wish to be included in my named report can be included as confidential comments to the editors, which will not be published.

Choose an item.

To further support our reviewers, we have joined with Publons, where you can gain additional credit to further highlight your hard work (see: https://publons.com/journal/530/gigascience). On publication of this paper, your review will be automatically added to Publons, you can then choose whether or not to claim your Publons credit. I understand this statement.

Yes Choose an item.
